# Exploring causal pathways between hypertension, lipid levels, and gout: Insights from Mendelian randomization and NHANES observations

**DOI:** 10.1097/MD.0000000000043638

**Published:** 2025-08-01

**Authors:** Mingyang Li, Qilong Nie, Kangle Lv, Jiaying Liu, Zeping Jiang

**Affiliations:** a The Eighth Clinical Medical College, Guangzhou University of Chinese Medicine, Foshan, Guangdong Province, China; b Gaoyao District People’s Hospital, Zhaoqing, Guangdong Province, China; c Foshan Hospital of Traditional Chinese Medicine, Guangzhou University of Chinese Medicine, Foshan, Guangdong Province, China.

**Keywords:** gout, hypertension, lipid levels, Mendelian randomization, NHANES

## Abstract

Gout, a common form of inflammatory arthritis, is caused by the deposition of monosodium urate crystals in joints and soft tissues. While associations between hypertension, lipid levels, and gout have been explored, their causal relationships remain unclear. This study aimed to examine the causal effects of hypertension and lipid levels on gout using multivariable Mendelian randomization (MR) and observational data from the National Health and Nutrition Examination Survey. We combined data from National Health and Nutrition Examination Survey (2006–2016) and genetic information from genome-wide association studies to investigate the associations between hypertension, lipid levels (high-density lipoprotein cholesterol, low-density lipoprotein cholesterol, triglycerides (TG), and total cholesterol), and gout. A 2-sample MR analysis was conducted using genetic instruments from genome-wide association studies and FinnGen datasets to determine the causal effects of hypertension and lipid levels on gout. In addition, multivariate Mendelian randomization was employed to simultaneously assess the causal impacts of multiple lipid traits and hypertension on gout. Sensitivity analyses were performed to assess the robustness of the findings. Observational analyses demonstrated a strong positive association between hypertension and gout risk (odds ratio [OR] = 5.35, 95% confidence interval [CI]: 4.45–6.37, *P* < .001). MR analysis further confirmed a causal relationship between hypertension (inverse variance weighting [IVW] OR = 2.68, 95% CI: 1.60–4.49, *P* < .001), TG (IVW OR = 1.37, 95% CI: 1.20–1.56, *P* < .001), low-density lipoprotein cholesterol (IVW OR = 1.34, 95% CI: 1.18–1.54, *P* < .001), and gout risk. Multivariate Mendelian randomization analysis indicated that while TG showed a significant causal effect on gout, hypertension and other lipid traits did not exhibit significant causal relationships in the multivariate framework. Sensitivity analyses affirmed the consistency and reliability of these findings. This study provides robust evidence for a significant causal relationship between hypertension, lipid levels, and gout, with TG emerging as a key factor in gout pathogenesis. These findings suggest that targeting hypertension and dyslipidemia, particularly TG, may serve as important strategies for the prevention and management of gout. Further research into the underlying biological pathways could offer new insights into gout pathogenesis and potential therapeutic interventions.

## Graphical abstract

**Figure:**
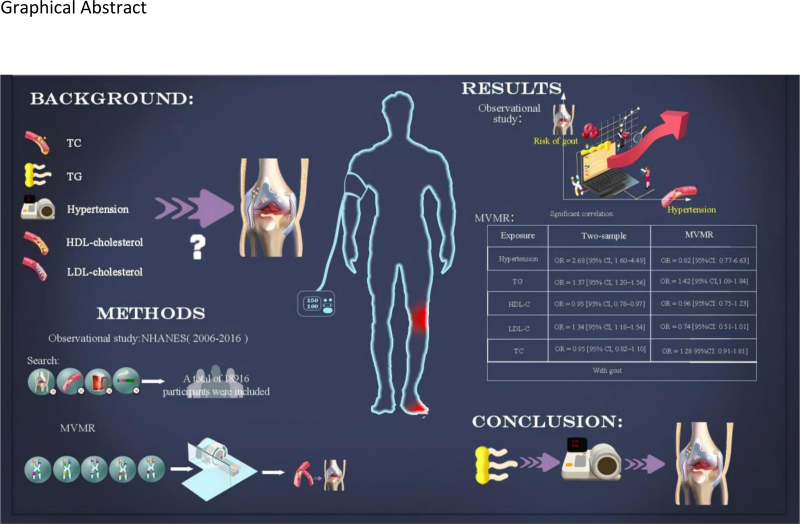

## 1. Introduction

Gout is a disorder resulting from abnormal metabolism of uric acid (UA), characterized by the deposition of UA crystals in joints or soft tissues, leading to acute arthritis and pain. Uric acid is a byproduct of purine metabolism, and elevated levels of UA in the body promote the accumulation of UA crystals within the joints, thereby precipitating gout attacks. Consequently, heightened levels of UA represent one of the primary risk factors for gout development.^[1,2]^

The pathogenesis underlying gout involves the deposition of monosodium urate crystals within joints and soft tissues, leading to acute and/or chronic inflammatory responses.^[3]^ In individuals with gout, an excessive production or inadequate excretion of UA results in hyperuricemia, which can precipitate the formation of urate crystals. These crystals precipitate within joints and surrounding tissues, eliciting inflammation and pain.^[4]^ The prevalence of gout demonstrates significant regional and ethnic disparities, with its incidence showing an upward trend, ranging from <1% to as high as 6.8% across diverse populations.^[5]^ Furthermore, the prevalence of gout varies significantly by race, age, and sex, with certain racial groups experiencing higher prevalence rates. The incidence of gout climbs with age and is more prevalent among men than women. However, postmenopause, women see an increase in prevalence, potentially due to the uricosuric effect of estrogen.^[6–8]^

A prospective study revealed a bidirectional association between hypertension and gout, suggesting that patients with hypertension had an 88% higher risk of developing gout, while those with gout had an 18% increased risk of developing hypertension.^[9]^ The findings of a longitudinal cohort study indicate that obesity, excessive weight gain during youth, and hypertension are significant risk factors for the development of gout. Implementing preventive measures to address obesity and hypertension can effectively reduce the incidence and prevalence of gout.^[10]^

Gout and lipid metabolism disorders are closely related, involving several lipid parameters, including high-density lipoprotein cholesterol (HDL-C), low-density lipoprotein cholesterol (LDL-C), triglycerides (TG), and total cholesterol (TC). First, gout is primarily associated with hyperuricemia, which leads to the accumulation of UA crystals in joints and other tissues, triggering inflammation. Research has shown that gout patients often have abnormal lipid profiles, particularly elevated TG and LDL-C levels, and reduced HDL-C levels.^[11]^ HDL-C, commonly referred to as “good cholesterol,” typically exhibits antioxidant and anti-inflammatory properties.^[12]^ Low HDL-C levels in gout patients may impair UA excretion, exacerbating the disease.^[13]^ On the other hand, LDL-C, known as “bad cholesterol,” tends to be elevated in gout patients and contributes to the inflammatory processes that are central to gout pathogenesis. Elevated LDL-C may also disrupt UA metabolism, further aggravating hyperuricemia.^[14]^ Moreover, high TG levels are common in gout patients and are closely linked to insulin resistance and metabolic syndrome, both of which are prevalent in gout. Elevated TG levels can inhibit the renal excretion of UA, promoting its accumulation in the body.^[15,16]^ As for TC, which represents the total amount of cholesterol in the blood, high TC levels are often observed in gout patients, especially those with accompanying metabolic disorders or cardiovascular diseases.^[17]^ The underlying mechanisms connecting gout and lipid metabolism disturbances likely involve shared metabolic pathways, such as insulin resistance and chronic low-grade inflammation. Insulin resistance not only reduces UA excretion but also leads to the accumulation of lipids in the bloodstream, further contributing to the lipid abnormalities seen in gout patients.

The National Health and Nutrition Examination Survey (NHANES), conducted by the Centers for Disease Control and Prevention’s National Center for Health Statistics, provides vital health and nutrition data for the United States. Its advanced multistage probability sampling ensures representative data across diverse demographics. Through comprehensive interviews, physical exams, and lab assessments by skilled professionals, NHANES offers critical insights into national health trends.^[6,18,19]^ Consequently, NHANES supplies high-quality, broad, and nationally indicative data, essential for analyzing the link between hypertension and gout risk.

Mendelian randomization (MR) uses genetic variants as instrumental variables (IVs) to identify causal relationships between risk factors and health outcomes, similar to a randomized controlled trial but within observational data.^[20]^ Since genetic variants are fixed from birth and randomly allocated at conception, MR minimizes confounding and bias that typically plague observational studies. This feature of MR allows it to provide more robust causal inferences than conventional observational methods, which are prone to confounding factors such as reverse causality and measurement error. By linking specific genetic instruments to risk factors, MR enables reliable determination of causal effects on diseases, thereby helping to distinguish true causal relationships from mere associations. In this study, MR plays a crucial role by uncovering biological mechanisms linking hypertension, lipid levels, and gout, and guiding targeted interventions in public health.^[21,22]^ Given that hypertension, lipid levels (HDL-C, LDL-C, TG, and TC), and gout follow identifiable Mendelian inheritance models, MR provides a powerful tool for understanding their causal relationships and potential therapeutic targets.

In this study, we integrated data from a large-scale observational study using NHANES 2006 to 2016 with MR analysis to thoroughly investigate the relationship between hypertension, lipid levels, and gout.

## 2. Methods

### 2.1. Reasearch on hypertension and gout in NHANES

The data analyzed in this study is available via the NHANES database (https://www.cdc.gov/nchs/nhanes/?CDC_AAref_Val=https://www.cdc.gov/nchs/nhanes/index.htm). NHANES procedures were approved by the National Center for Health Statistics Research Ethics Review Board, and all participants provided informed consent. This research was exempt from institutional review board approval as it used anonymized, publicly accessible data. Participants who responded affirmatively to the question “Ever told you had high blood pressure?” are categorized as having hypertension. Participants were assessed for gout based on the following criterion: “Has your doctor ever told you that you have gout?” Those who answered “yes” were classified as having gout.

To mitigate potential confounding effects, adjustments were made for the following demographic characteristics: age, gender, race, education level, ratio of family income to poverty, smoking, drinking and diabetes status, body mass index, waist circumference, aspartate aminotransferase, alanine aminotransferase, TG, gamma-glutamyl transferase, TC, HDL-C, LDL-C, creatinine, UA, and C-reactive protein (CRP). The smoking status of the participants was ascertained by evaluating whether they had a lifetime history of smoking at least 100 cigarettes. The participants’ drinking status was determined based on their consumption of a minimum of 12 alcohol drinks per annum. For the diagnosis of diabetes, participants were asked by their doctor, “Do you have diabetes?” Those who answered “yes” were classified as diabetic. The levels of aspartate aminotransferase, gamma-glutamyl transferase, and alanine aminotransferase are measured in units per liter (U/L), the measurements of TG, TC, HDH-C, and LDL-C are all reported in mmol/L units, the concentration of creatinine is measured in µmol/L units, and the measurements of both UA and CRP are expressed in units of mg/dL.

### 2.2. Reasearch on hypertension, lipid levels, and gout in genome-wide association study (GWAS) sources

Hypertension, lipid levels (HDL-C, LDL-C, TG, and TC), and gout all exhibit Mendelian inheritance patterns, which makes them suitable for analysis using MR. Specifically, high blood pressure and lipid traits, such as TG and LDL-C, are polygenic traits, where multiple genetic variants influence the phenotype in a complex manner. A variety of single nucleotide polymorphisms (SNPs) associated with hypertension and lipid levels have been identified through large-scale GWAS, providing strong evidence that these SNPs directly influence these traits.^[23–27]^ Similarly, gout is influenced by genetic variants involved in urate metabolism, which follow Mendelian inheritance patterns, as demonstrated by studies in familial cases and large genetic consortia, supporting the application of MR to assess causal effects between these traits. Since genetic variants are randomly assigned at conception, MR methods are particularly well-suited for investigating causal relationships between hypertension, lipid levels, and gout. This is because MR helps minimize confounding factors and reverse causality, which are common concerns in traditional observational studies.

For each exposure (TG, TC, HDL-C, and LDL-C), we selected SNPs that were strongly associated with the respective trait in the GWAS data. We did not limit our analysis to SNPs associated with multiple traits; rather, we included SNPs that were most strongly correlated with each individual exposure. This approach ensured that the SNPs used in the analysis were specifically relevant to the trait being studied, minimizing any potential confounding effects that might arise from SNPs shared across different traits. Therefore, the SNPs selected for hypertension, lipid levels (TG, TC, HDL-C, and LDL-C), and gout were not restricted to those linked to multiple traits, but to those demonstrating the strongest associations with each specific exposure.

Genetic variants strongly associated with hypertension were obtained from the MRC Integrated Epidemiology Unit consortium, which includes data from 462,933 Europeans (119,731 cases and 343,202 controls). Gout-related genetic data came from a FinnGen GWAS with 3576 cases and 147,221 controls, all of European descent. Genetic association data for TC involved 437,878 Europeans, while GWAS data for TG and LDL-C were sourced from 441,016 and 201,678 European participants, respectively, via the UK Biobank. HDL-C data from 403,943 Europeans were also sourced from the UK Biobank. GWAS data were accessed through the MRC Integrated Epidemiology Unit’s OpenGWAS database.^[28,29]^ All studies had ethical approvals, with informed consent obtained from participants.

**Figure 1.**
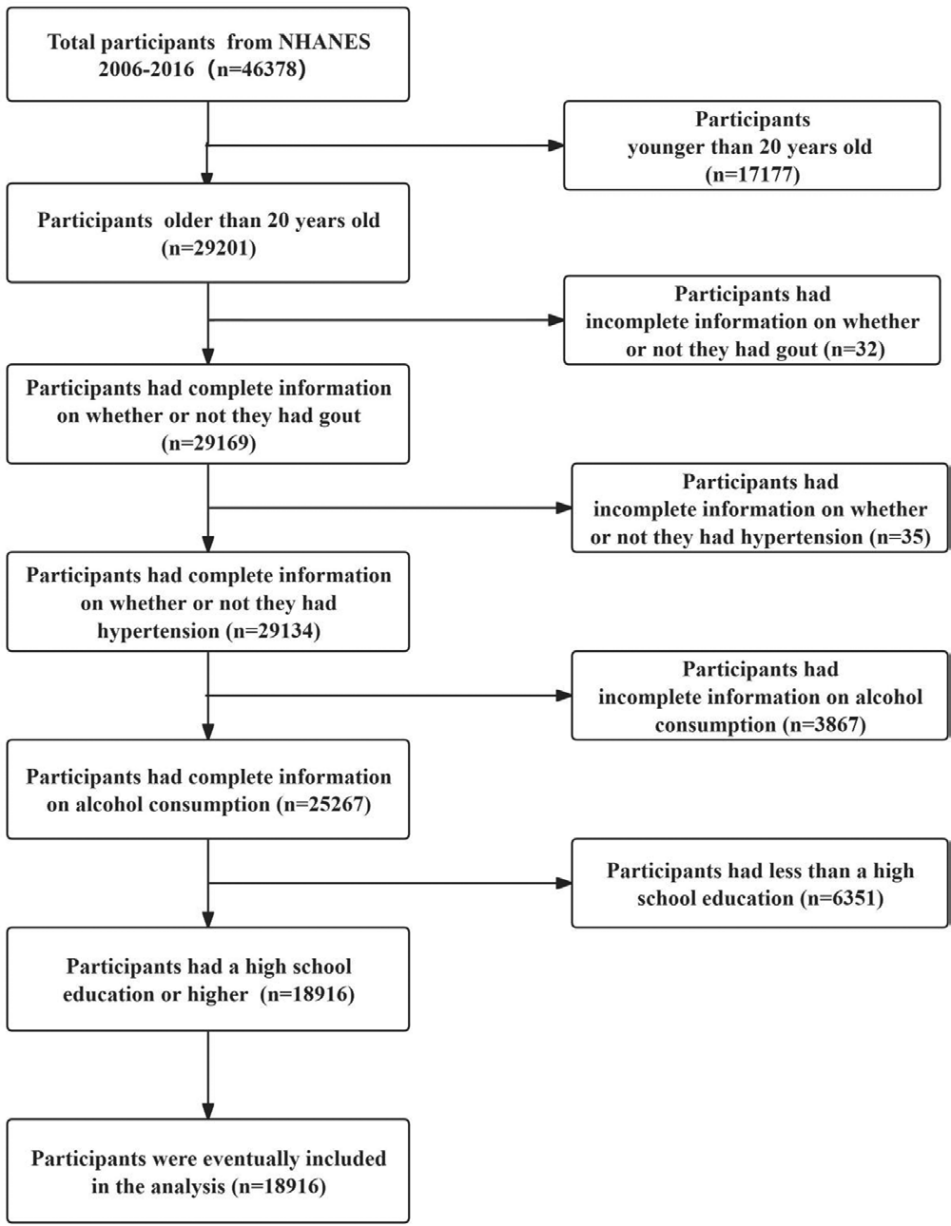
Flowchart of participants’ inclusion.

To discern genetic variants for estimating the causal links between gout and certain exposures (hypertension, TC, TG, HDL-C and LDL-C), SNPs were selected as IVs for the analyses. This study adhered to the 3 critical assumptions of 2-sample MR: (1) All chosen IVs demonstrated a strong correlation with the exposure (*P* < 5 × 10^−8^, *F* statistic >10), and the *F*-statistic is calculated using the formula: $F=[R^{2}\times (N-2)]/(1-R^{2})$; (2) The IVs were not associated with confounding factors between the exposure and outcome; we excluded SNPs that showed linkage disequilibrium with an *R*^2^ < 0.001 across a span of 10,000 kb; *R*^2^ represents the proportion of exposure variance each IV explains. *R*^2^ is determined by 2 × EAF × (1 − EAF) × *β*^2^, with *β* representing the allele effect size and EAF the effect allele frequency. Subsequently, we employed the PhenoScanner database or LDlink (https://ldlink.nci.nih.gov/?tab=ldtrait) to scrutinize each SNP, with the goal of removing any that were significantly linked to potential confounders or traits associated with gout. The IVs influenced the outcome exclusively through the exposure, without other intervening pathways (Fig. 1).

The number of SNPs selected for each exposure was based on their statistical significance and relevance to the trait. We included only those SNPs that were significantly associated with each exposure (*P* < 5 × 10^−8^) and met the *F*-statistic threshold (*F*-statistic >10) to ensure robust and reliable genetic instruments. These SNPs were chosen to represent the most significant genetic variants associated with each exposure, ensuring adequate statistical power for the MR analysis. The final number of SNPs selected for each trait were 297 for hypertension, 464 for TG, 303 for TC, 632 for HDL-C, and 460 for LDL-C. This selection process balanced the need for robust genetic instruments while avoiding the inclusion of too many weakly associated SNPs that could compromise the results.

Regarding SNPs associated with multiple traits, we specifically focused on SNPs that were strongly associated with the exposure being studied. While some SNPs may be related to multiple traits, we ensured that the selected SNPs were primarily associated with the trait of interest. For example, SNPs associated with both hypertension and lipid levels were included only if they met the strong association criteria for each specific exposure. This strategy allowed us to focus on the most relevant genetic variants for each exposure while avoiding the introduction of confounding factors that might arise from SNPs influencing multiple traits.

In the end, we extracted 297, 464, 303, 632, and 460 SNPs for hypertension, TG, TC, HDL-C, and LDL-C, respectively, for further causal analysis. Estimates of the effects of the association between gene predicted exposure and gout risk are detailed in Table S1, Supplemental Digital Content, https://links.lww.com/MD/P550. The detailed overview of the data downloading and filtering process is presented in Table S3, Supplemental Digital Content, https://links.lww.com/MD/P550.

### 2.3. Statistical analysis

This study analyzed the relationship between hypertension and gout using data from the NHANES through a weighted logistic regression framework across 3 models. Model 1 assessed hypertension and gout relationship; Model 2 built upon Model 1 by further evaluating the relationship between hypertension and gout, incorporating demographic and socioeconomic factors such as age, gender, race, education level, and the ratio of family income to poverty. Model 3 extended the analysis of the relationship between hypertension and gout by including lifestyle and clinical variables, such as smoking and alcohol consumption status, diabetes status, and UA levels, in addition to the factors considered in Models 1 and 2. Preprocessing addressed missing data and ensured compliance with logistic regression assumptions. Specifically, missing data were handled by deleting records with incomplete information. This approach was chosen to maintain the integrity of the dataset and ensure the robustness of the analysis. Additionally, preprocessing involved variable transformation and standardization to align with logistic regression requirements, detection and handling of outliers, and verification of model assumptions. Associations were determined using the Wald test (*P* < .05 indicating significance), and model fit was assessed with the Akaike information criterion (AIC), with results presented as odds ratio (OR) or *β* (95% confidence intervals [CI]). Considering the complex probability clustering design of NHANES, the study accounted for weights in the statistical analysis.

In this study, we developed a series of regression models to investigate the relationship between hypertension, lipid levels, and gout, while accounting for demographic, socioeconomic, lifestyle, and clinical variables. To address potential multicollinearity among the lipid variables (HDL-C, TG, LDL-C, and TC), we applied principal component analysis to transform these correlated lipid variables into uncorrelated principal components. We retained the first 2 principal components (PC1 and PC2), as they explained the majority of the variance in the lipid data. These components were then used in place of the original lipid variables in the regression models. PC1 captures the largest amount of variation in the lipid data and represents the overall lipid profile, effectively reflecting the combined effects of all lipid variables. PC2, which is orthogonal to PC1, represents distinct patterns of lipid levels, capturing the second-largest variation in the data. Specifically, PC2 is characterized by high values when HDL and TG levels are elevated, while TC is low. Model 1 assessed the association between lipid levels (represented by PC1 and PC2) and gout, providing a baseline understanding of how these lipid patterns individually influence gout risk. In Model 2, we extended Model 1 by adding hypertension status as an independent variable. This model evaluated the combined effects of hypertension and lipid levels on gout and explored how hypertension modifies the relationship between lipid levels and gout. Model 3 built upon Model 2 by including additional demographic and socioeconomic factors such as age, gender, race, education level, and the ratio of family income to poverty. This model aimed to control for these potential confounders and evaluate the independent effects of hypertension and lipid levels on gout, after adjusting for these variables. Model 4 further expanded Model 3 by incorporating lifestyle and clinical variables, including smoking status, alcohol consumption, diabetes status, and UA levels. This comprehensive model allowed for a more detailed assessment of the relationship between hypertension, lipid levels, and gout by adjusting for lifestyle and clinical factors. To check for multicollinearity among the independent variables, we calculated the variance inflation factor (VIF). Variables with a VIF >10 were considered to exhibit high multicollinearity and were subject to further examination or removal. The VIF analysis indicated severe multicollinearity among the lipid variables, which prompted the use of principal component analysis to reduce the correlation between these variables and prevent multicollinearity issues in the modeling process. Given the potential presence of outliers and heteroscedasticity in the data, we employed robust linear models using Huber T norm. Robust regression methods are less sensitive to outliers and provide more reliable estimates when data exhibit nonconstant variance, improving the robustness and validity of our findings.

In our 2-sample MR analysis, we used inverse variance weighting (IVW) to evaluate the causal relationships between hypertension, TG, TC, HDL-C, LDL-C, and gout risk. We validated IVW results with complementary methods like MR-Egger and weighted median. To ensure robustness, we performed sensitivity analyses, including Cochran *Q* test^[30]^ for heterogeneity and the MR-Egger intercept for pleiotropy. Significant heterogeneity (*P* < .05) was addressed using the random-effects IVW method. The MR-PRESSO method corrected pleiotropy by identifying and removing pleiotropic SNPs, with consistent causal estimates. We also employed weighted median and MR-Egger for robust estimates, even with up to 50% invalid instruments. A leave-one-out analysis^[31]^ confirmed no single SNP significantly influenced the results. These sensitivity analyses, including heterogeneity and pleiotropy assessments, ensured the reliability of our findings, supporting the identified causal relationships between hypertension, lipid levels, and gout.

In our multivariate Mendelian randomization (MVMR) analysis, we specifically adjusted for the 4 lipid variables (TG, TC, HDL-C, and LDL-C) as confounding factors to obtain accurate and unbiased estimates of the causal effects of hypertension on gout. These lipid variables were chosen due to their well-documented associations with both hypertension and gout. TG and cholesterol levels are known to influence cardiovascular health and metabolic processes, which are also related to hypertension, while dyslipidemia is a common feature observed in individuals with gout.^[32,33]^ By adjusting for these lipid variables, we control for potential confounding and isolate the direct effect of hypertension on gout, ensuring our observed associations reflect true causal relationships. This approach also allows us to assess the independent contributions of each lipid variable and hypertension to gout risk, enhancing the validity of our inferences. This comprehensive adjustment highlights the complex biological pathways involved and underscores the robustness of our findings, providing a clearer understanding of the relationships between hypertension, lipid levels, and gout.

The primary analysis method was IVW, our main tool for assessing causality. To ensure robustness, we also applied MR-Egger and weighted median techniques. MVMR analyses allowed us to isolate the independent effects of multiple risk factors on gout while adjusting for confounders. This approach provided a refined understanding of each factor’s unique contribution to the disease, accounting for shared pathways. The use of MVMR enhanced causal inference by offering a clearer view of the direct effects of each risk factor on gout, independent of other pathways.

The advantages and disadvantages of MR method are summarized in Table S4, Supplemental Digital Content, https://links.lww.com/MD/P550. The statistical analyses were conducted using R software, version 4.3.1 (R Foundation, Vienna, Austria), and EmpowerStats software (X&Y Solutions Inc., Boston).

## 3. Results

### 3.1. Demographic and clinical characteristics in the NHANES study were statistically based on gout

**Figure 2.**
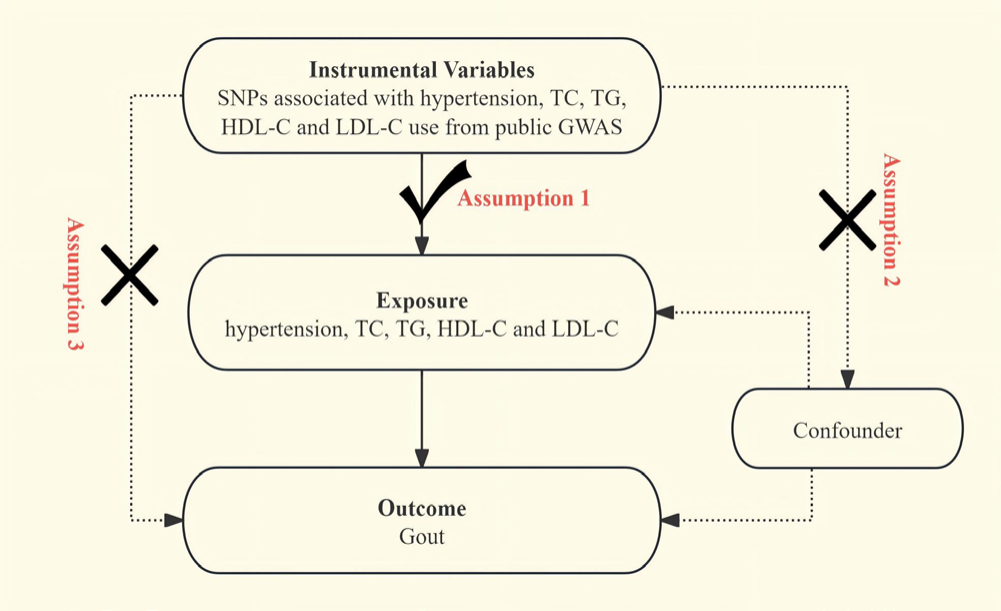
Foundations of Mendelian genetics; the 3 assumptions of randomization.

The study included 29,201 participants aged 20 or older who visited the NHANES Mobile Examination Center between 2006 and 2016. After excluding individuals with incomplete data on gout (n = 32), hypertension (n = 35), alcohol consumption (n = 3867), and education level (n = 6351), the final sample consisted of 18,916 participants. Details are shown in Figure 2.

According to demographic and clinical characteristics from the NHANES data in Table 1, it shows that among 850 participants with gout and 18,066 without, those with gout were generally older, predominantly male, and more likely to be non-Hispanic White. Gout prevalence was lower among individuals with higher education levels. Smoking and alcohol consumption significantly increased gout risk, with smokers and drinkers showing higher incidence rates. Gout was nearly 3 times more common among alcohol consumers and more prevalent in individuals with hypertension, emphasizing the link between lifestyle factors, health conditions, and gout risk.

**Table 1:** Demographic and clinical characteristics of the participants with and without gout.

Items	Gout (n = 850)	No gout (n = 18066)	*P*-value
Age (yr)	60.7 (59.6–61.8)	46.6 (46.1–47.2)	<.0001
Gender			<.001
Male	604 (71.1%)	8602 (47.6%)	
Female	246 (28.9%)	9464 (52.4%)	
Race			<.001
Mexican American	37 (4.4%)	1826 (10.1%)	
Other Hispanic	44 (5.2%)	1657 (9.2%)	
Non-Hispanic White	468 (55.1%)	8608 (47.6%)	
Non-Hispanic Black	219 (25.8%)	3850 (21.3%)	
Other race	82 (9.6%)	2125 (11.8%)	
Education level			.037
High school	295 (34.7%)	5451 (30.2%)	
Some college or AA degree	324 (38.1%)	7035 (38.9%)	
College graduate or above	231 (27.2%)	5555 (30.7%)	
Refused	0 (0.0%)	6 (0.0%)	
Don’t know	0 (0.0%)	19 (0.1%)	
Ratio of family income to poverty	3.2 (3.1–3.3)	3.2 (3.1–3.4)	.9589
Smoker status			<.001
Yes	482 (56.7%)	7620 (42.2%)	
No	368 (43.3%)	10–434 (57.8%)	
Refused	0 (0.0%)	1 (0.0%)	
Don’t know	0 (0.0%)	11 (0.1%)	
Drinking state			.066
Yes	655 (77.1%)	13–293 (73.6%)	
No	195 (22.9%)	4763 (26.4%)	
Don’t know	0 (0.0%)	10 (0.1%)	
BMI	32.0 (31.2–32.7)	28.8 (28.6–29.0)	<.0001
Waist circumference (cm)	109.8 (108.3–111.4)	98.6 (98.1–99.1)	<.0001
Diabetes			<.001
Yes	262 (30.8%)	1868 (10.3%)	
No	557 (65.5%)	15,796 (87.4%)	
Refused	31 (3.6%)	391 (2.2%)	
Don’t know	0 (0.0%)	11 (0.1%)	
Hypertension			<.001
Yes	633 (74.5%)	6024 (33.3%)	
No	217 (25.5%)	12,042 (66.7%)	
Laboratory features			
AST (U/L)	28.1 (27.1–29.1)	25.5 (25.3–25.8)	<.0001
GGT (IU/L)	40.1 (35.9–44.4)	26.3 (25.6–27.0)	<.0001
ALT (U/L)	27.3 (26.0–28.7)	25.3 (25.0–25.6)	.005
TG (mmol/L)	1.9 (1.7–2.0)	1.4 (1.3–1.4)	<.0001
TC (mmol/L)	4.9 (4.8–5.0)	5.0 (5.0–5.1)	.0047
HDL-C (mmol/L)	1.2 (1.2–1.3)	1.4 (1.4–1.4)	<.0001
LDL-C (mmol/L)	2.7 (2.6–2.9)	3.0 (2.9–3.0)	.0012
Cr (µmol/L)	98.4 (92.4–104.4)	77.7 (77.1–78.3)	<.0001
UA (mg/dL)	320.6 (318.7–322.6)	390.8 (381.2–400.4)	<.0001
CRP (mg/dL)	0.5 (0.3–0.7)	0.4 (0.3–0.4)	.168

Data were presented as median (interquartile range) or n (%). Mann–Whitney *U* test for continuous variables. Pearson chi-squared test for categorical variables.

AA = Associate of Arts, ALT = alanine aminotransferase, AST = aspartate transaminase, BMI = body mass index, Cr = creatinine, CRP = C-reactive protein, GGT = gamma-glutamyl transferase, HDL-C = high-density lipoprotein cholesterol, LDL-C = low-density lipoprotein cholesterol, TC = total cholesterol, TG = triglycerides, UA = uric acid.

### 3.2. Observational associations between hypertension, lipid levels, and gout in NHANES

Our weighted logistic regression analysis demonstrates a strong and significant link between hypertension and the risk of gout, consistent across a suite of models adjusting for various covariates. Initially, Model 1 reveals signifying that individuals with hypertension are over 5 times more likely to develop gout (OR = 5.35, 95% CI: 4.49–6.37). Model 2, which accounts for lipid profiles, yields a slightly lower, implying that lipid levels may partially mediate the hypertension-gout connection, although the association remains significant (OR = 5.14, 95% CI: 3.85–6.86). The introduction of demographic and socioeconomic factors (age, gender, race, education, and income relative to poverty [Model 3]) further reduces the OR to 3.28 (95% CI: 2.33–4.62), shedding light on their independent effects on the risk of gout. Model 4 maintains the OR at 3.28 even after adjusting for lifestyle and clinical variables, including smoking, alcohol consumption, diabetes, UA, and CRP levels, confirming the steady impact of these factors. Notwithstanding the diminished association after each adjustment, the enduring statistical significance reinforces hypertension’s role as an independent predictor of gout. This indicates a nuanced interplay among biological, demographic, and lifestyle elements in the etiology of gout.

**Figure 3.**
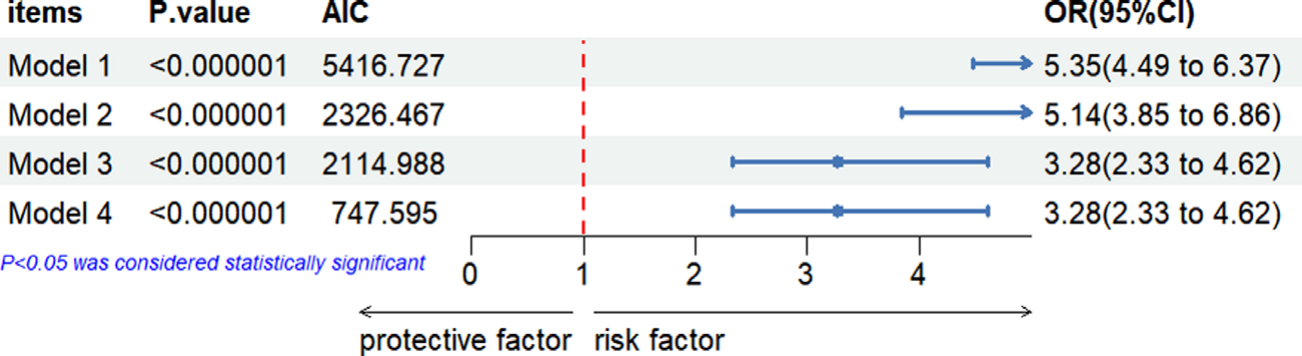
Odds ratios (ORs) for the association between hypertension and gout across multiple logistic regression models adjusted for various covariates. AIC = Akaike information criterion, CI = confidence interval.

In our investigation into the relationship between hypertension and gout, we employed the AIC to discern the most informative model amidst a series comprising progressively complex adjustments. Starting with an unadjusted model (Model 1) that presented an AIC of 5416, we systematically introduced adjustments for lipid profiles (Model 2, AIC = 2326), demographic and socioeconomic factors (Model 3, AIC = 2114), and, finally, lifestyle and clinical variables (Model 4, AIC = 747). The substantial decrease in AIC values with each subsequent model underscored the vital importance of these variables in accurately depicting the relationship at hand. Notably, Model 4 emerged as the superior model, characterized by its comprehensive inclusion of variables and the lowest AIC, signifying an optimal balance between model complexity and fit. This rigorous approach illuminates the multifaceted nature of the hypertension-gout nexus, highlighting the utility of an encompassing model that integrates lipid profiles, demographic and socioeconomic factors, alongside lifestyle and laboratory indicators, to provide a nuanced understanding of gout’s pathogenesis and its association with hypertension.The detailed values are displayed in Figure 3.

**Figure 4.**
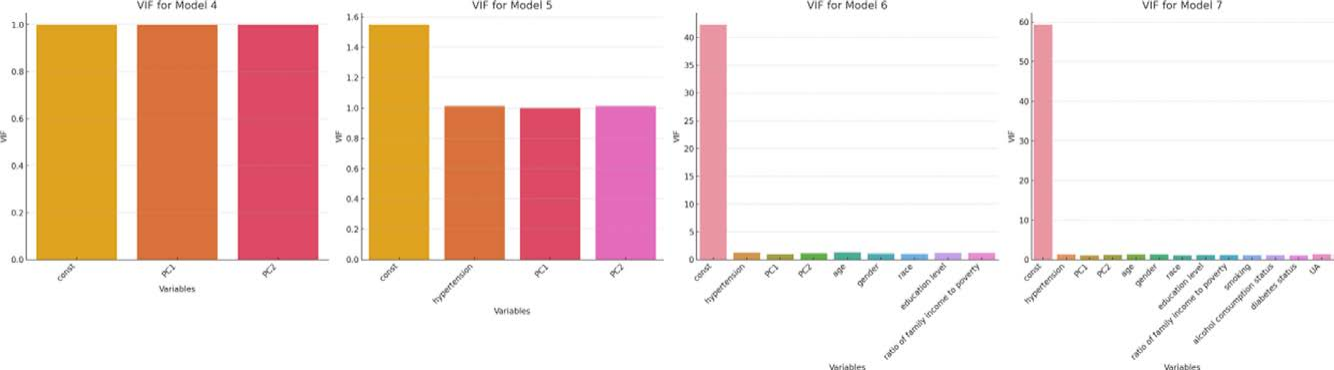
The VIF values for Models 4 to 7. VIF = variance inflation factor.

The results of the regression analysis for Models 1 to 7 are shown in Table 2, including the *β* coefficients, standard errors, 95% CI, *z*-values, and *P*-values. Model 1 examined the relationship between lipid levels (represented by PC1 and PC2) and gout. The *β* for PC1 and PC2 were −7.462 × 10^−54^ and −4.804 × 10^−53^, respectively, both statistically significant (*P* < .001). Model 2 extended Model 1 by including hypertension status. The *β* for hypertension was 1.253 × 10^−46^, with a *z*-value of 20.403 (*P* < .001). The *β* for PC1 and PC2 remained similar to Model 1, both statistically significant. Model 3 added demographic and socioeconomic factors. The *β* for hypertension was 2.235 × 10^−48^, with a *z*-value of 27.576 (*P* < .001). Age, gender, and race were also significant predictors, with *z*-values of 28.196, 19.880, and 5.903, respectively. VIF values indicated no significant multicollinearity issues. Model 4 incorporated lifestyle and clinical variables. The *β* for hypertension was 1.251 × 10^−46^, with a *z*-value of 20.416 (*P* < .001). Other significant predictors included smoking, alcohol consumption, diabetes status, and serum UA, with *z*-values of 3.463, −1.192, 2.233, and 4.233, respectively. VIF values again showed no significant multicollinearity. We assessed multicollinearity using the VIF, with all values below 10, indicating no issues. Model diagnostics included residuals vs fitted values plots and Q-Q plots, which confirmed homoscedasticity and approximate normality of the residuals (Fig. 4).

**Table 2:** The results of the regression analysis for Models 1 to 4.

Variable	*β*	Standard error	OR	95% CI	*z*-value	*P*-value
Model 1						
PC1	−7.462 × 10^−54^	1.16 × 10^−54^	NA	(−9.74 × 10^−54^, −5.18 × 10^−54^)	−6.413	<.001
PC2	−4.804 × 10^−53^	1.41 × 10^−54^	NA	(−5.08 × 10^−53^, −4.53 × 10^−53^)	−34.039	<.001
Model 2						
Hypertension	1.253 × 10^−46^	6.14 × 10^−48^	NA	(1.13 × 10^−46^, 1.37 × 10^−46^)	20.403	<.001
PC1	−7.462 × 10^−54^	1.16 × 10^−54^	NA	(−9.74 × 10^−54^, −5.18 × 10^−54^)	−6.413	<.001
PC2	−4.804 × 10^−53^	1.41 × 10^−54^	NA	(−5.08 × 10^−53^, −4.53 × 10^−53^)	−34.039	<.001
Model 3						
Hypertension	2.235 × 10^−48^	8.1 × 10^−50^	NA	(2.08 × 10^−48^, 2.39 × 10^−48^)	27.576	<.001
PC1	−2.762 × 10^−49^	2.42 × 10^−50^	NA	(−3.24 × 10^−49^, −2.29 × 10^−49^)	−11.425	<.001
PC2	−4.758 × 10^−49^	3.11 × 10^−50^	NA	(−5.37 × 10^−49^, −4.15 × 10^−49^)	−15.321	<.001
Model 4						
Hypertension	1.251 × 10^−46^	6.13 × 10^−48^	NA	(1.13 × 10^−46^, 1.37 × 10^−46^)	20.416	<.001
PC1	−2.608 × 10^−47^	1.81 × 10^−48^	NA	(−2.96 × 10^−47^, −2.25 × 10^−47^)	−14.376	<.001
PC2	−1.921 × 10^−47^	2.36 × 10^−48^	NA	(−2.38 × 10^−47^, −1.46 × 10^−47^)	−8.129	<.001

95% CI = 95% confidence interval, OR = odds ratio.

### 3.3. Causal relationships between hypertension, lipid levels, and gout in MR

Given that our multivariate regression analysis identified a significant positive association between hypertension and the risk of gout. For further verification, we embarked on a 2-sample MR study; this investigation aimed to causally analyze the connection between hypertension and 4 specific lipid metrics (TG, TC, LDL-C, and HDL-C) with respect to gout.

#### 3.3.1. Two-sample MR analysis

**Figure 5.**
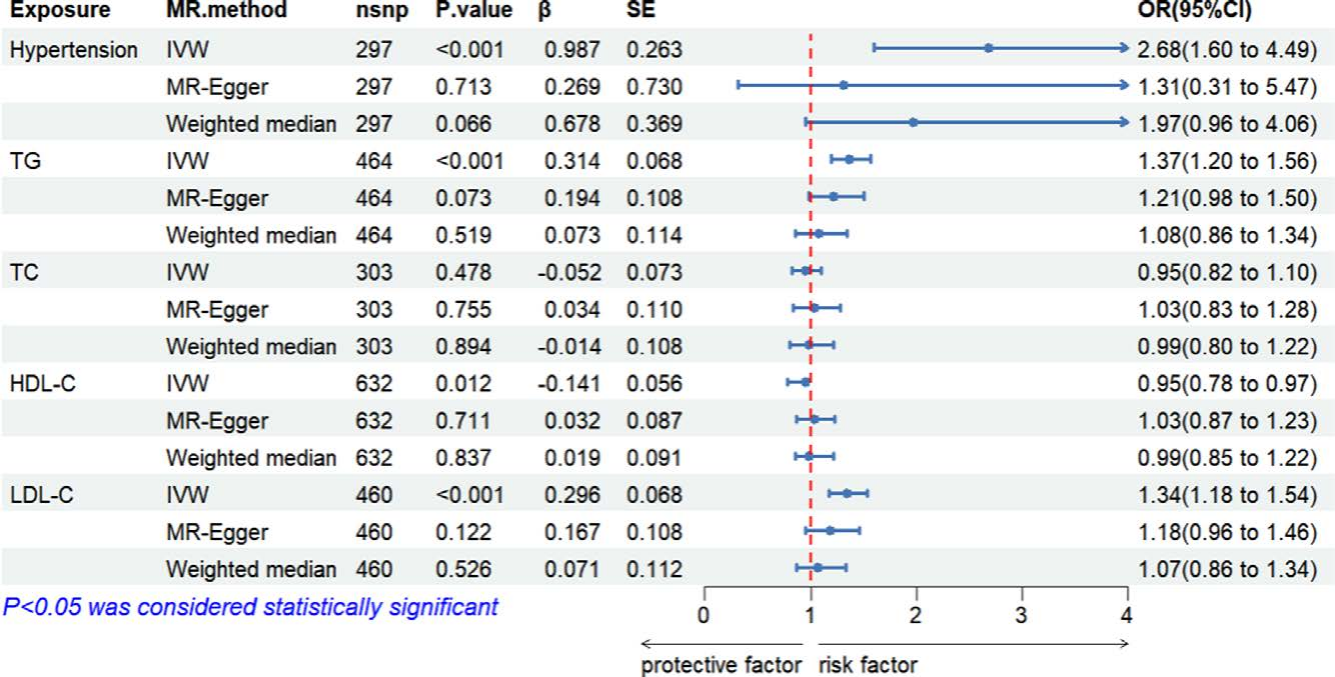
Two-sample Mendelian randomization analysis of gout exposure effects.

As illustrated in Figure 5, the findings reveal a clear and robust causal link between hypertension (IVW OR = 2.68, 95% CI: 1.60–4.49, *P* < .001), TG (IVW OR = 1.37, 95% CI: 1.20–1.56, *P* < .001), LDL-C (IVW OR = 1.34, 95% CI: 1.18–1.54, *P* < .001), HDL-C (IVW OR = 0.95, 95% CI: 0.78–0.97, *P* = .012), TC (IVW OR = 0.95, 95% CI: 0.82–1.10, *P* = .48), and gout, as determined through the IVW method. We observed a heterogeneity test *P*-value of <.05, indicating substantial heterogeneity across the genetic instruments employed to infer the causal impact of the exposure on the outcomes. To assess whether the 5 SNP-related exposures could potentially induce gout via alternate pathways, we conducted a horizontal pleiotropy analysis. The results of the MR-Egger test revealed no evidence of pleiotropy across these 4 exposures (*P* > .05); however, a significant Egger regression intercept (*P* = .009) suggests potential horizontal pleiotropy, indicating that genetic variants associated with HDL-C may influence gout risk through alternative pathways beyond HDL-C metabolism; Table S2, Supplemental Digital Content, https://links.lww.com/MD/P550 contain detailed values. Moreover, the symmetry of the funnel plot supports the absence of pleiotropy in the 5 exposures, as depicted in Figures S1–S5, Supplemental Digital Content, https://links.lww.com/MD/P550. Additionally, the leave-one-method analysis was performed to corroborate these results, as illustrated in Figures S6–S10, Supplemental Digital Content, https://links.lww.com/MD/P550. These methods provide a more robust assessment in the presence of pleiotropic genetic variants. Scatter plots illustrate the estimated effects of exposure on gout across different MR methods (Figures S11–S15, Supplemental Digital Content, https://links.lww.com/MD/P550). Forest plots are utilized to depict the impact of each SNP on gout (Figures S16–S20, Supplemental Digital Content, https://links.lww.com/MD/P550).

#### 3.3.2. Multivariate MR analysis

After conducting our initial 2-sample MR analysis, we proceeded to expand our investigation using MVMR framework. This methodology allows us to account for both potential confounding effects of related exposures. The MVMR analysis incorporates the IVW method, the median method, and MR-Egger regression to ensure robustness and comprehensiveness.

**Figure 6.**
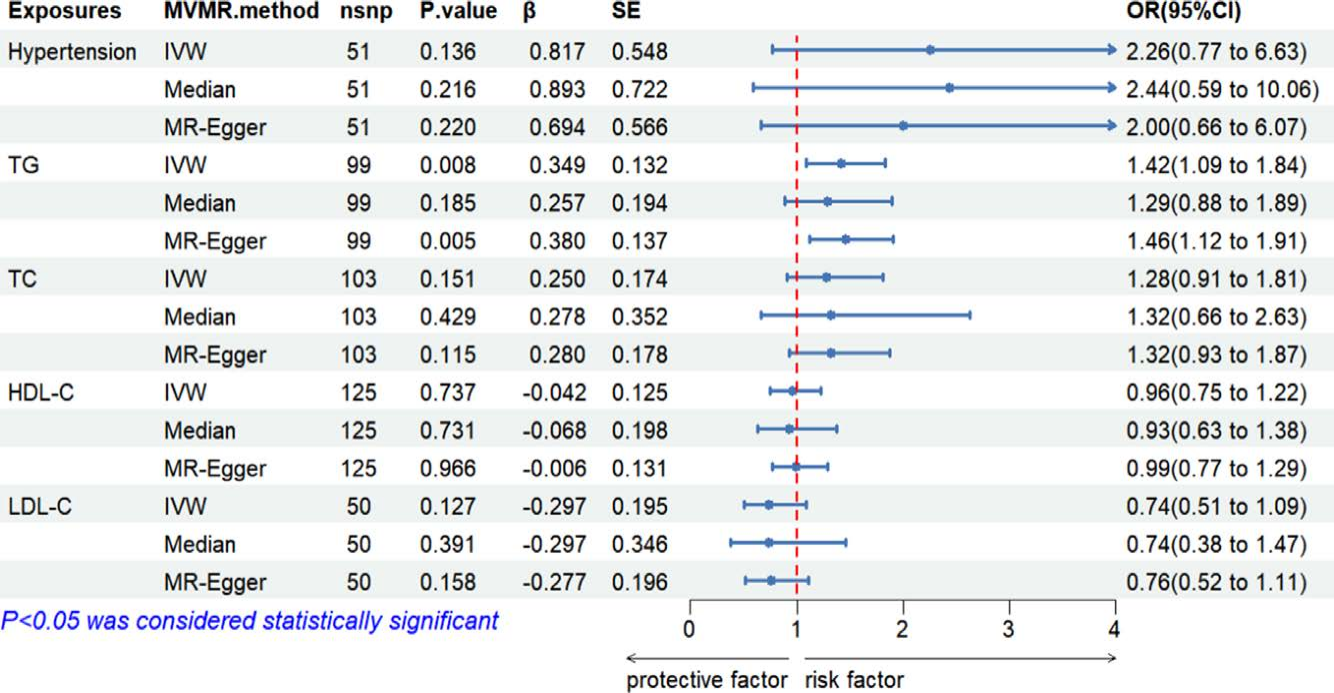
Multivariate Mendelian randomization analysis of gout exposure effects.

In the MVMR analysis, associations between various exposures and gout were explored. While TG showed a significant association with gout across IVW method (*P* = .008, OR = 1.42, 95% CI: 1.09–1.84), hypertension, TC, HDL-C, and LDL-C did not exhibit significant associations. Specifically, hypertension displayed nonsignificant associations across IVW method (*P* = .14, OR = 0.82, 95% CI: 0.77–6.63), as did TC (*P* = .25, OR = 1.28, 95% CI: 0.91–1.81), HDL-C (*P* = .74, OR = 0.96, 95% CI: 0.75–1.23), and LDL-C (*P* = .13, OR = 0.74, 95% CI: 0.51–1.01). These findings suggest a significant relationship between TG and gout, while other lipid and hypertension variables did not demonstrate notable associations with gout in this multivariate analysis (Fig. 6).

The causal relationship between hypertension and gout was found to be significant in the 2-sample MR analysis, but not in the MVMR analysis. This discrepancy may arise from potential unaccounted confounders in the 2-sample analysis, whereas MVMR accounted for these confounders by considering other relevant exposures and mediating effects. Moreover, MVMR is capable of identifying mediating effects where one variable influences the outcome through its influence on other variables. Additionally, as multiple exposures are considered in multivariate analyses, they can capture more complex interactions that might not have been fully captured in 2-sample analyses. Consequently, it is possible that the results obtained from the multivariate analysis appear inconsistent with those from the 2-sample analysis.

## 4. Discussion

To the best of our knowledge, this study is the first to utilize the MR approach to elucidate the causal relationships between hypertension, lipid levels, and gout, along with their potential underlying mechanisms. We successfully identified significant causal relationships and validated these findings within our cohort, thereby strengthening the evidence for these associations. The integration of GWAS data with NHANES data provided a comprehensive and robust framework to dissect these complex interactions. This finding underscores the multifaceted nature of gout pathogenesis, highlighting how metabolic and cardiovascular factors intricately interplay to influence disease development. The study’s strength lies in its rigorous methodology, combining large-scale NHANES observational data with detailed GWAS genetic data, enhancing the validity and reliability of our findings. Our research fills gaps in understanding the causal relationships and genomic context of these interactions. This study not only identifies direct effects but also explores mediation within the genomic landscape, offering insights that could inform targeted interventions. Future research should validate these findings across diverse populations and explore additional mediators and mechanisms to deepen understanding and improve personalized therapeutic approaches.

Hypertension and gout share a complex, multifactorial relationship driven by several interconnected biological mechanisms. One of the most significant mechanisms is the impact of hypertension on kidney function. Chronic high blood pressure leads to endothelial dysfunction, which impairs the blood flow to the kidneys and accelerates the development of renal microvascular damage.^[34]^ This damage reduces the kidneys’ ability to excrete UA efficiently, contributing to hyperuricemia. Additionally, hypertension-induced renal injury results in the upregulation of renal tubular reabsorption of UA, which further exacerbates elevated serum UA levels, a key risk factor for gout.^[35]^ Moreover, hypertension is frequently accompanied by insulin resistance, a condition that worsens UA retention. Insulin resistance reduces the efficiency of the renal clearance of UA by inhibiting the activity of urate transporters in the renal tubules.^[36]^ Insulin resistance also stimulates increased production of UA in the liver by enhancing purine synthesis pathways. This combination of reduced renal excretion and increased UA production leads to a state of hyperuricemia, setting the stage for the crystallization of monosodium urate in joints, a hallmark of gout.^[37]^ Another crucial pathway linking hypertension to gout involves oxidative stress and inflammation.^[38]^ Hypertension contributes to the activation of various inflammatory signaling pathways, including the NF-κB and IL-6 pathways, which increase the production of pro-inflammatory cytokines. These cytokines can stimulate the activation of immune cells like neutrophils, which are involved in the inflammatory response to urate crystals in gout. The influx of inflammatory cells into the joints, where urate crystals have deposited, leads to the acute inflammatory attacks characteristic of gout.^[39,40]^

A positive correlation was observed in an 8-year prospective cohort study between hypertension, hyperlipidemia, and hyperuricemia.^[41]^ A meta-analysis of 11 cohort studies has concluded that individuals with high blood pressure have a significantly increased risk of developing gout, more than doubling the likelihood compared to those without hypertension.^[42]^ A case-control study using data from the UK Biobank also showed that gout was independently correlated with hypertension and hyperlipidemia.^[43]^ The results of a case-control study involving 152,663 individuals with gout and 709,981 matched controls revealed that patients with gout exhibited a significantly elevated risk of dyslipidemia and hypertension compared to the matched controls.^[44]^ The epidemiological and comorbidity studies of gout indicate a notably high prevalence of hypertension among individuals with gout, with up to 3-quarters of patients exhibiting elevated blood pressure levels.^[45]^ Our observational findings from NHANES 2006 to 2016 also indicate a strong association between hypertension and an increased risk of gout, suggesting a significant role of hypertension as an independent predictor. Multivariate logistic regression analysis showed a significant positive association between hypertension and gout after adjusting for potential confounders, which was consistent with previous findings.

However, due to the intricate interplay among hypertension, blood lipid levels, and gout, establishing causal inference becomes challenging.^[46]^ Therefore, in addition to conducting an observational study using NHANES 2006 to 2016, we further employed the MR method to investigate the causal relationship between hypertension, blood lipid levels, and gout. MR analysis revealed a substantial causal association between hypertension, lipid levels, and gout. In addition to the primary IVW method, the supplementary MR approach yielded consistent findings. Sensitivity analysis further substantiated the positive association between robustness and reliability. Notably, the mediation analysis revealed that hypertension mediates approximately 23.32% of the effect of TG on gout risk. This confluence of data from both observational and genetic analyses underscores the multifactorial nature of gout, pinpointing hypertension and TG as critical factors in its pathogenesis and presenting them as potential targets for intervention to reduce gout incidence.

The study’s detailed exploration of the connections between hypertension, lipid levels, and gout highlights the significant strength of utilizing observational data from NHANES alongside MR analysis. This dual approach, combining NHANES data with genetic insights, enhances the reliability of our findings and illuminates novel metabolic pathways implicated in gout. Our study has several limitations. First, the reliance on NHANES data, which reflects U.S. demographics, and MR analysis based on European cohorts may limit the generalizability of our findings to other populations. Future research should include a broader ethnic and geographical range to ensure the universality of insights into gout pathogenesis. Additionally, while our methodology minimizes potential confounding, MR assumptions like the absence of pleiotropy and the focus on specific risk factors without considering broader influences, such as diet or renal function, may limit the scope of our conclusions. The use of FinnGen data, primarily from a Finnish population, could further restrict the applicability of our results to other ethnicities. Incorporating more diverse samples and combining data from additional databases would improve the robustness and generalizability of our findings. Finally, reliance on a single data source may introduce biases, suggesting the need for integrating multiple datasets in future research to enhance external validity. Addressing these limitations will improve the applicability and reliability of our conclusions.

## 5. Conclusions

In conclusion, this study using NHANES 2006 to 2016 data and MVMR reveals significant causal links between hypertension and gout. These findings highlight the importance of managing hypertension and TG in gout prevention. However, further validation and exploration of underlying mechanisms are essential, emphasizing the need for broader research across diverse populations and additional risk factors to better understand gout’s complex dynamics.

## Acknowledgments

The authors express their gratitude to the participants and coordinators of NHANES, MRC-IEU, the UK Biobank, and FinnGen Consortium for their pivotal role in facilitating this research through their exceptional dataset.

## Author contributions

**Conceptualization:** Mingyang Li, Qilong Nie.

**Data curation:** Qilong Nie.

**Formal analysis:** Mingyang Li, Kangle Lv.

**Funding acquisition:** Zeping Jiang.

**Investigation:** Mingyang Li, Jiaying Liu.

**Methodology:** Mingyang Li, Jiaying Liu.

**Resources:** Kangle Lv.

**Software:** Jiaying Liu.

**Visualization:** Qilong Nie.

**Writing – original draft:** Mingyang Li, Qilong Nie, Zeping Jiang.

**Writing – review & editing:** Zeping Jiang.

## Supplementary Material


