# Supplementary material for: Exploring causal pathways between hypertension, lipid levels, and gout: Insights from Mendelian randomization and NHANES observations
Source: Medicine (Baltimore). 2025 Aug 1;104(31):e43638. doi: 10.1097/MD.0000000000043638 (PMC12323965; doi:10.1097/MD.0000000000043638)
Supplement: Supplementary file 1 [file medi-104-e43638-s001.pdf]

Exploring Causal Pathways Between Hypertension, Lipid Levels, and Gout: Insights from Mendelian Randomization and NHANES Observations

Mingyang Li[1#], Qilong Nie[1#], , Kangle Lv[2], Jiaying Liu[2], Zeping Jiang[2, *]

Page 2: Supplementary materials Figure 1 Funnel diagram of hypertension

Page 3: Supplementary materials Figure 2 Funnel diagram of TG

Page 4: Supplementary materials Figure 3 Funnel diagram of TC

Page 5: Supplementary materials Figure 4 Funnel diagram of HDL-C

Page 6: Supplementary materials Figure 5 Funnel diagram of LDL-C

Page 7: Supplementary materials Figure 6 leave-one-method of hypertension on gout

Page 8: Supplementary materials Figure 7 leave-one-method of TG on gout

Page 9: Supplementary materials Figure 8 leave-one-method of TC on gout

Page 10: Supplementary materials Figure 9 leave-one-method of HDL-C on gout

Page 11: Supplementary materials Figure 10 leave-one-method of LDL-C on gout

Page 12: Supplementary materials Figure 11 Scatter plots of hypertension on gout

Page 13: Supplementary materials Figure 12 Scatter plots ofTG on gout

Page 14: Supplementary materials Figure 13 Scatter plots ofTC on gout

Page 15: Supplementary materials Figure 14 Scatter plots of HDL-C on gout

Page 16: Supplementary materials Figure 15 Scatter plots of LDL-C on gout

Page 17: Supplementary materials Figure 16 Forest plots of hypertension on gout

Page 18: Supplementary materials Figure 17 Forest plots ofTG on gout

Page 19: Supplementary materials Figure 18 Forest plots ofTC on gout

Page 20: Supplementary materials Figure 19 Forest plots of HDL-C on gout

Page 21: Supplementary materials Figure 20 Forest plots of LDL-C on gout

Page 22-99: Supplementary materials Table 1 Estimates of the effects of the association between gene predicted exposure and gout risk

Page 100: Supplementary materials Table 2 Evaluating Pleiotropy in Hypertension,TG, TC, LDL-C, HDL-C and Gout Link via Mendelian Randomization

Page101: Supplementary materials Table 3. Characteristics of GWAS participants included in the MR study.

Page 102-103: Supplementary materials Table 4. Summary of each Mendelian randomization method.

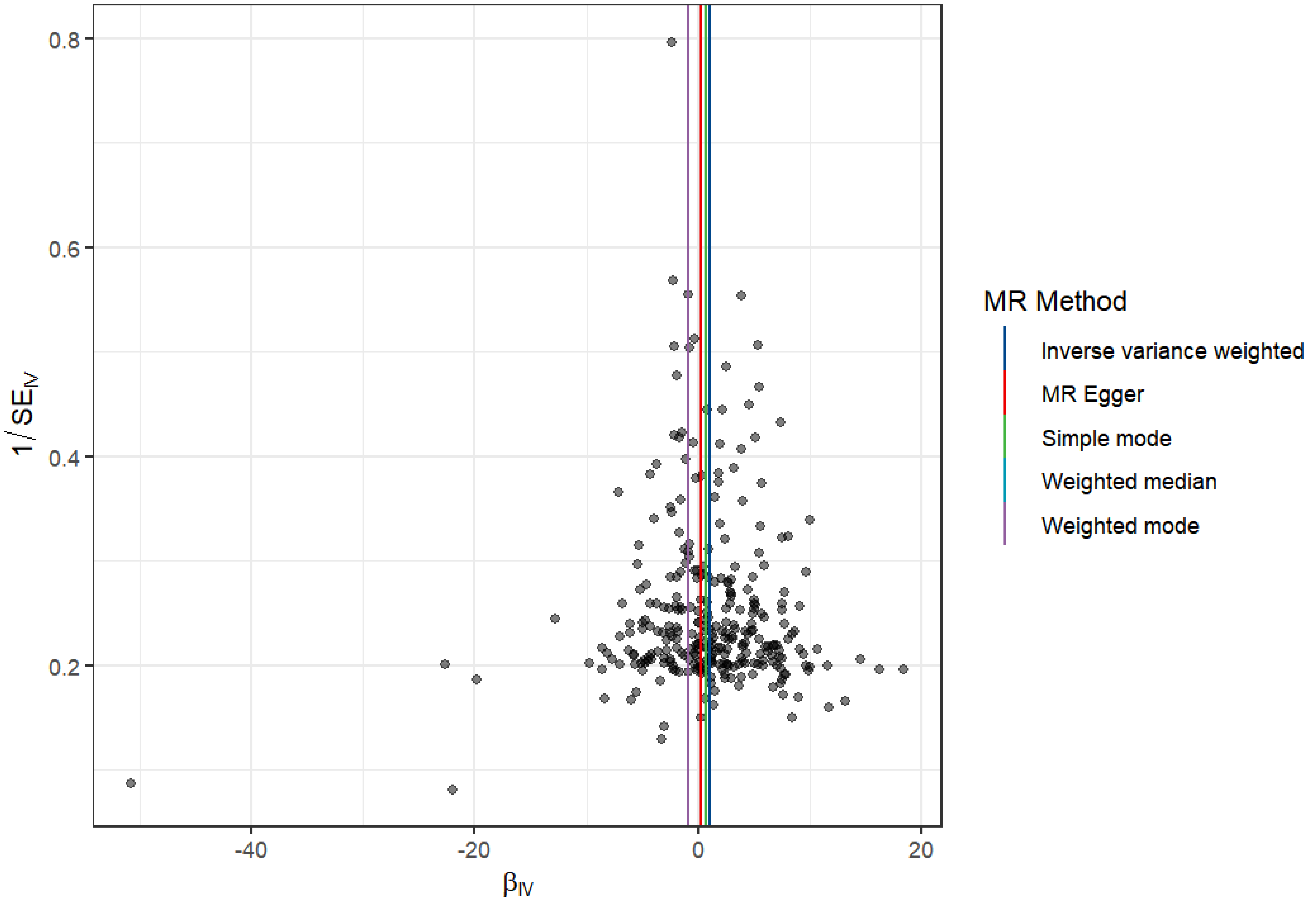

0.8
0.6
0.4
0.2
1 / SE_{IV}
-40
-20
0
20
β_{IV}
MR Method
Inverse variance weighted
MR Egger
Simple mode
Weighted median
Weighted mode

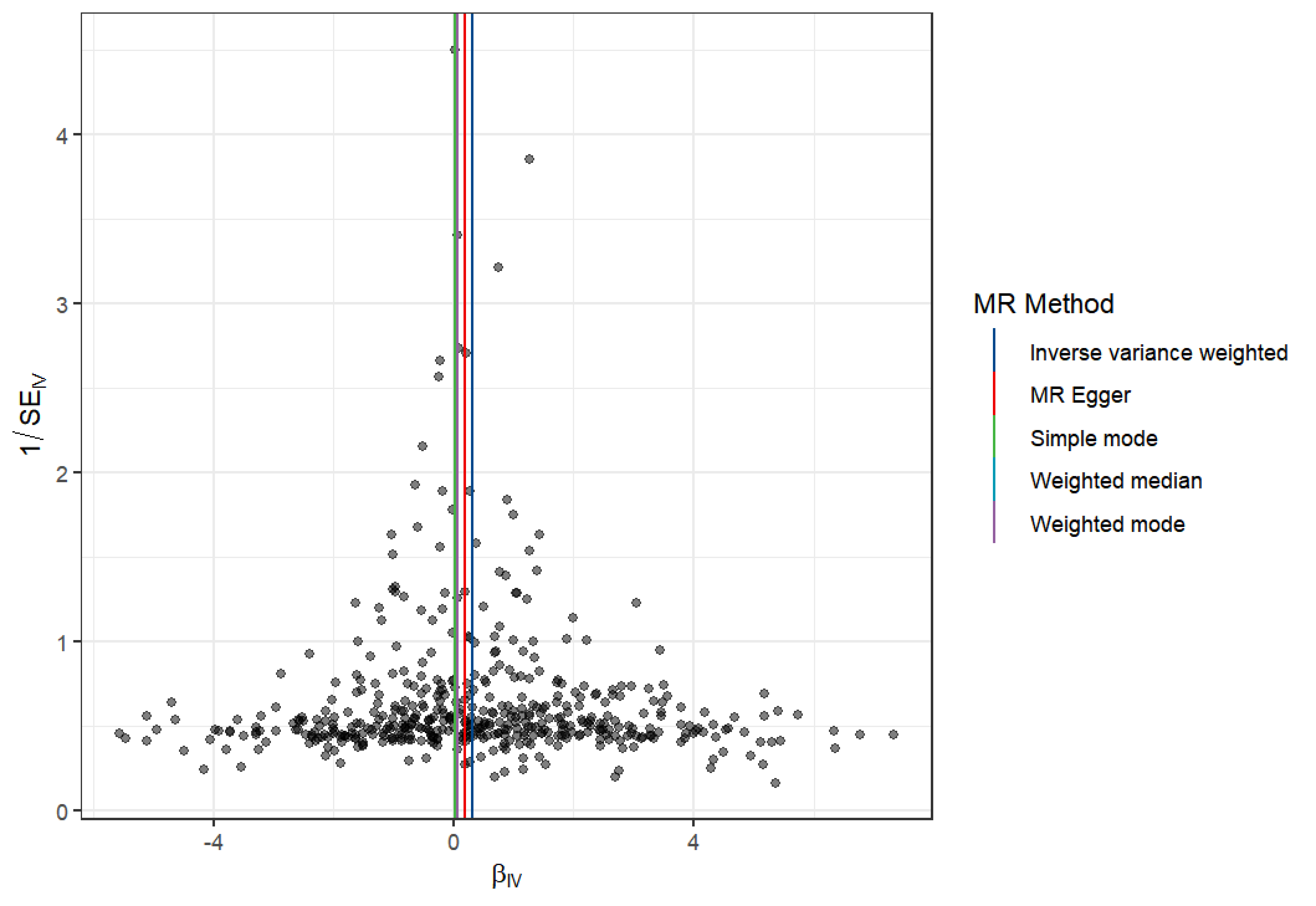

MR Method
Inverse variance weighted
MR Egger
Simple mode
Weighted median
Weighted mode
1/SE_IV
β_IV
0
1
2
3
4
-4
0
4

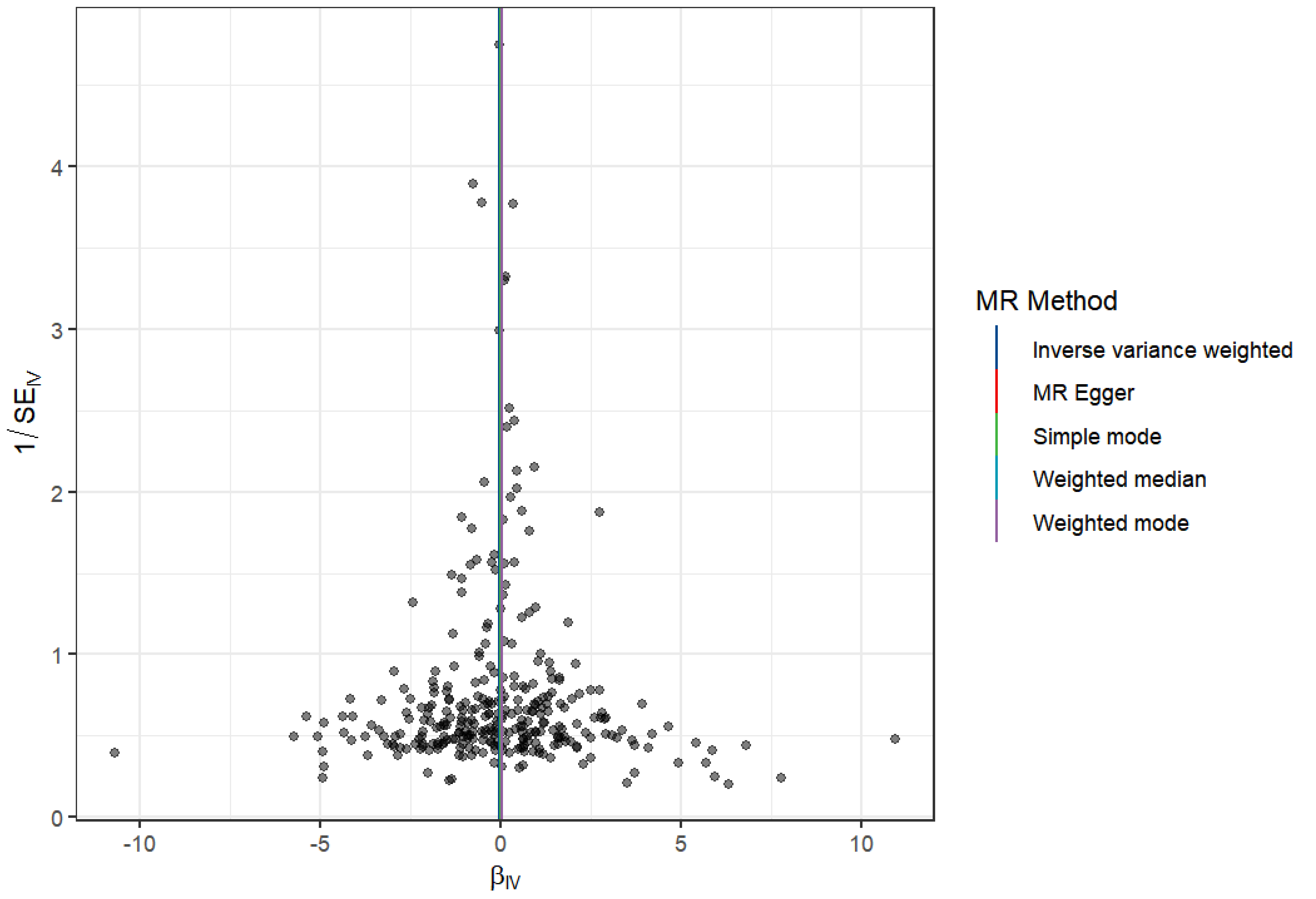

4
3
2
1
0
1 / SE_{IV}
-10
-5
0
5
10
β_{IV}
MR Method
Inverse variance weighted
MR Egger
Simple mode
Weighted median
Weighted mode

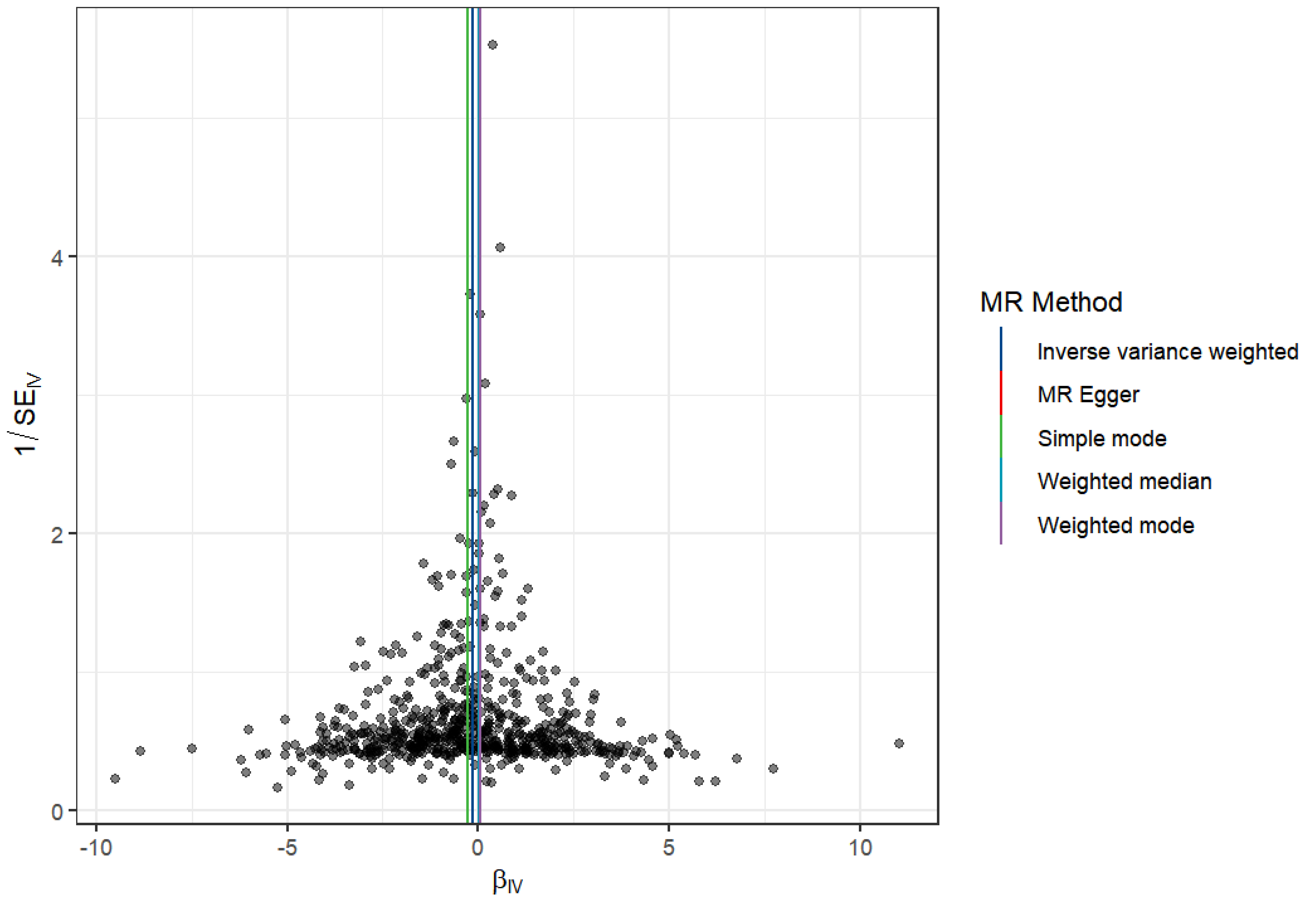

4
2
0
-10
-5
0
5
10
β_{IV}
$1/SE_{IV}$
MR Method
Inverse variance weighted
MR Egger
Simple mode
Weighted median
Weighted mode

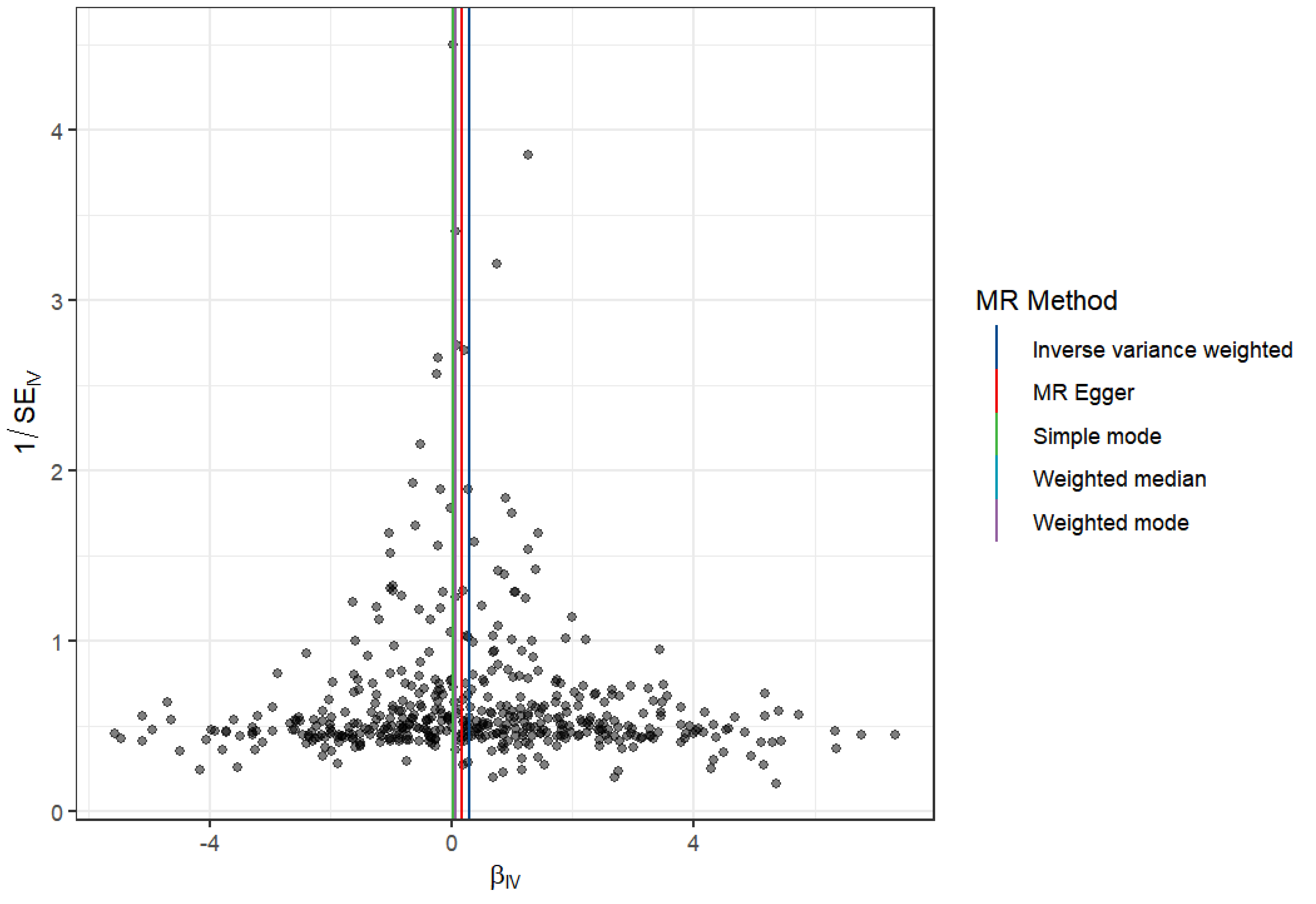

1/SE_IV
β_IV
4
3
2
1
0
-4
0
4
MR Method
Inverse variance weighted
MR Egger
Simple mode
Weighted median
Weighted mode

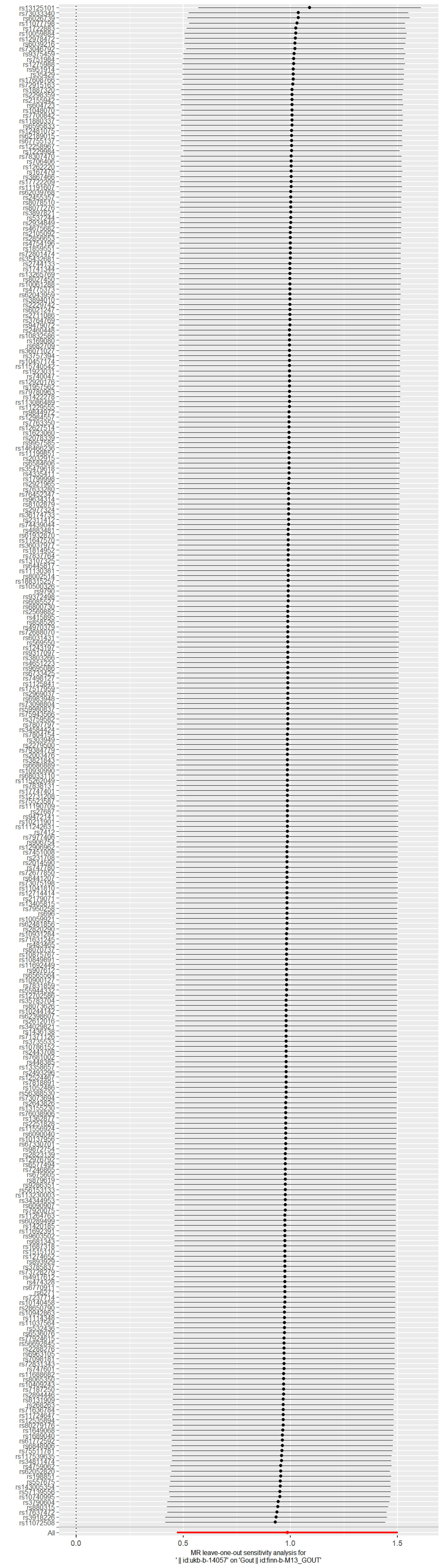
All
0.0
0.5
1.0
1.5
MR leave-one-out sensitivity analysis for
' || id:ukb-b-14057' on 'Gout || id:finn-b-M13_GOUT'

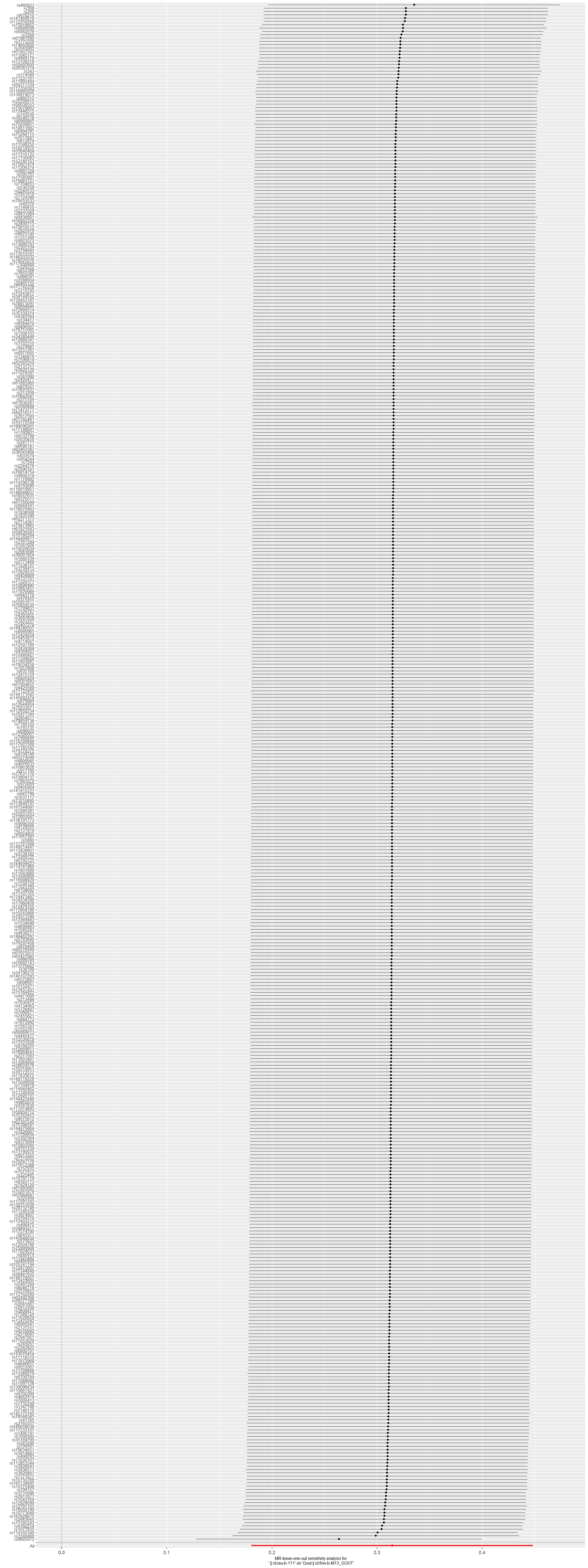
rs480823
All
0.0
0.1
0.2
0.3
0.4
MR leave-one-out sensitivity analysis for
' || id:ieu-b-111' on 'Gout || id:finn-b-M13_GOUT'

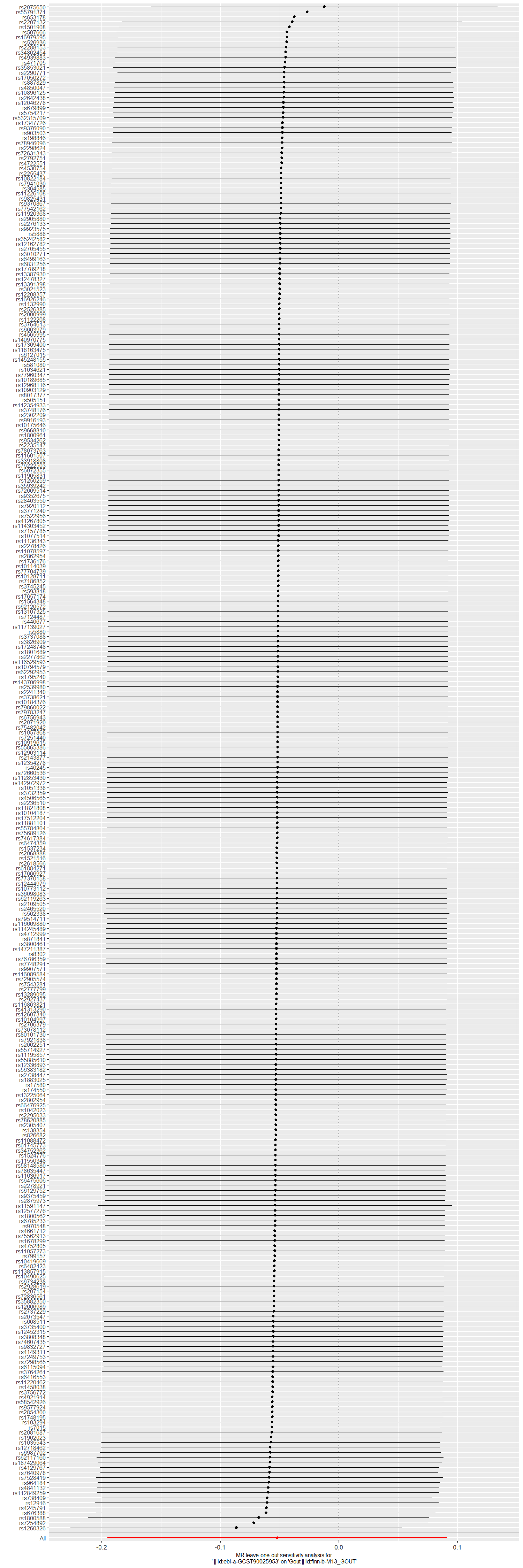
All
-0.2
-0.1
0.0
0.1
MR leave-one-out sensitivity analysis for
' || id:ebi-a-GCST90025953' on 'Gout || id:finn-b-M13_GOUT'

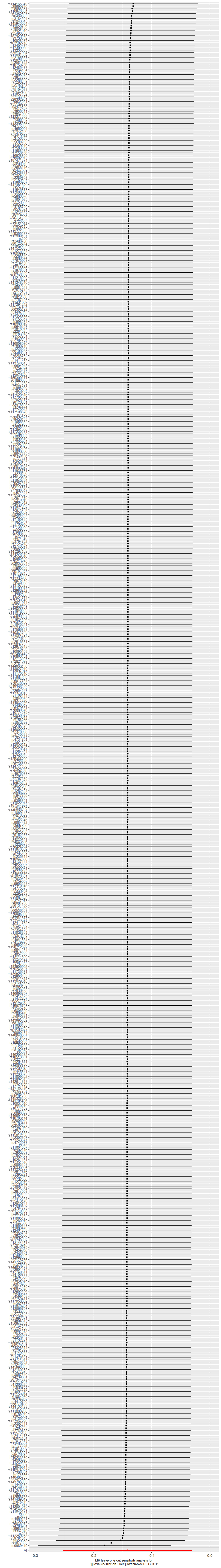
All
-0.3
-0.2
-0.1
0.0
MR leave-one-out sensitivity analysis for
' || id:ieu-b-109' on 'Gout || id:finn-b-M13_GOUT'

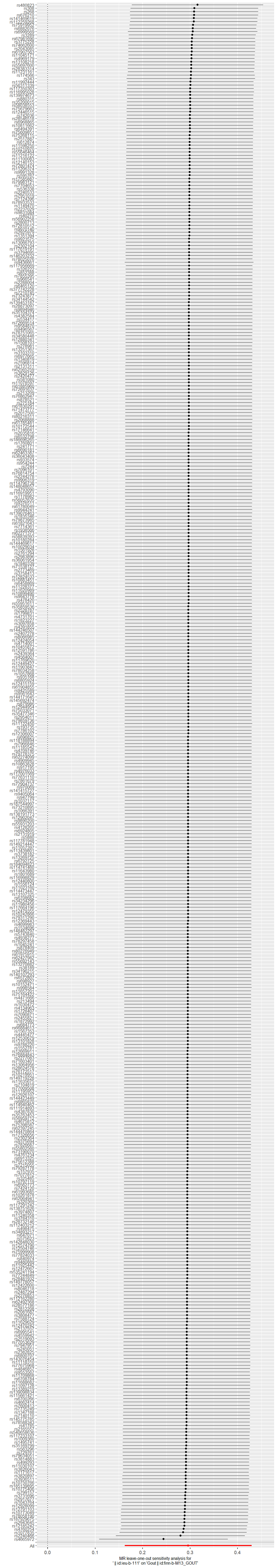

rs480823
rs3814883
rs499293
rs11030107
rs3826043
rs1171617
rs3820897
rs3936511
rs10750766
rs185139895
rs10775406
rs799157
rs3731696
rs2917677
rs1043764
rs12928099
rs12791103
rs10773049
rs78058190
rs187929675
rs2943645
rs12185242
rs4760254
rs1077835
rs2240466
rs4665972
All
0.0
0.1
0.2
0.3
0.4
MR leave-one-out sensitivity analysis for
' || id:ieu-b-111' on 'Gout || id:finn-b-M13_GOUT'

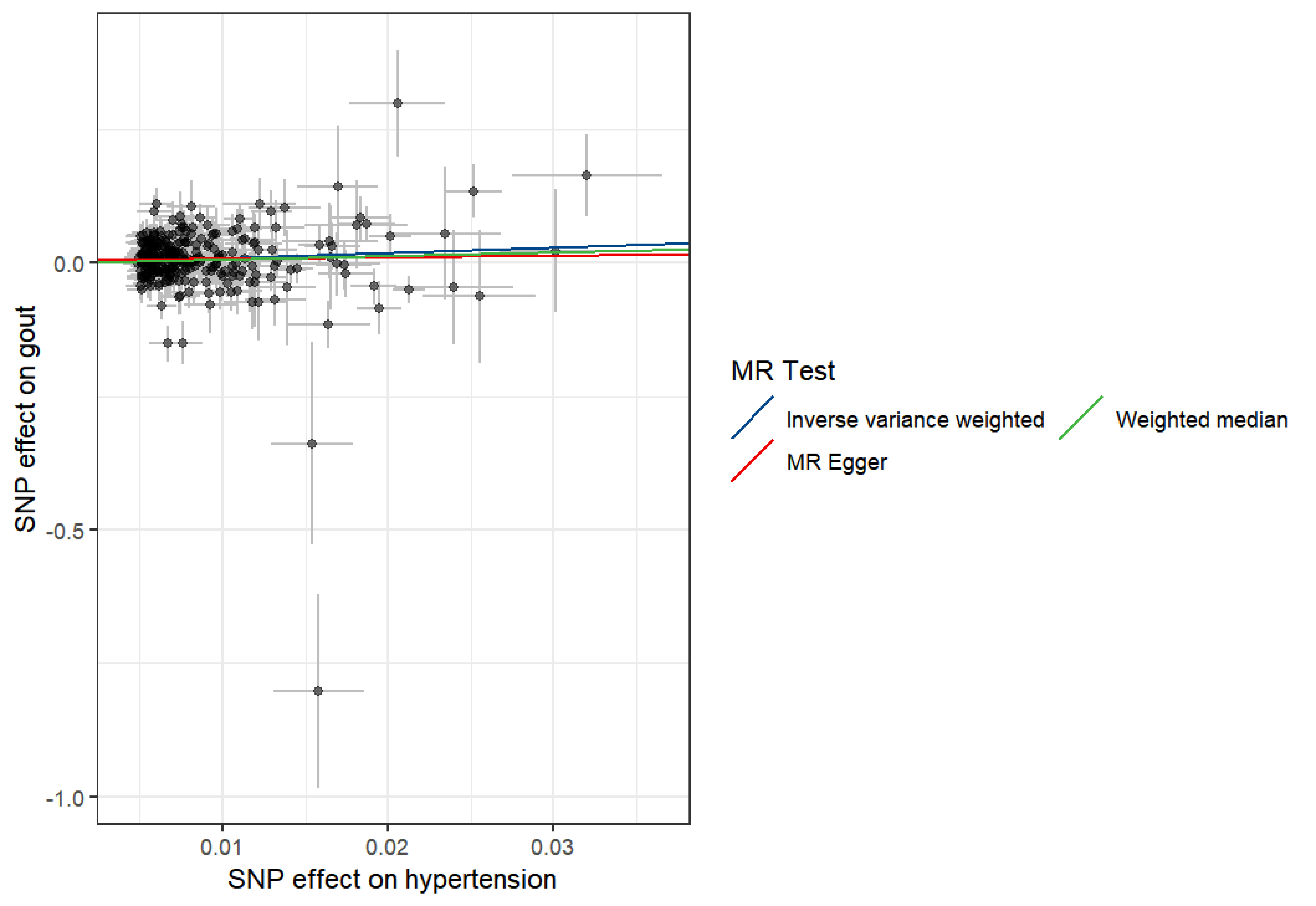

SNP effect on gout
0.0
-0.5
-1.0
0.01
0.02
0.03
SNP effect on hypertension
MR Test
Inverse variance weighted
Weighted median
MR Egger

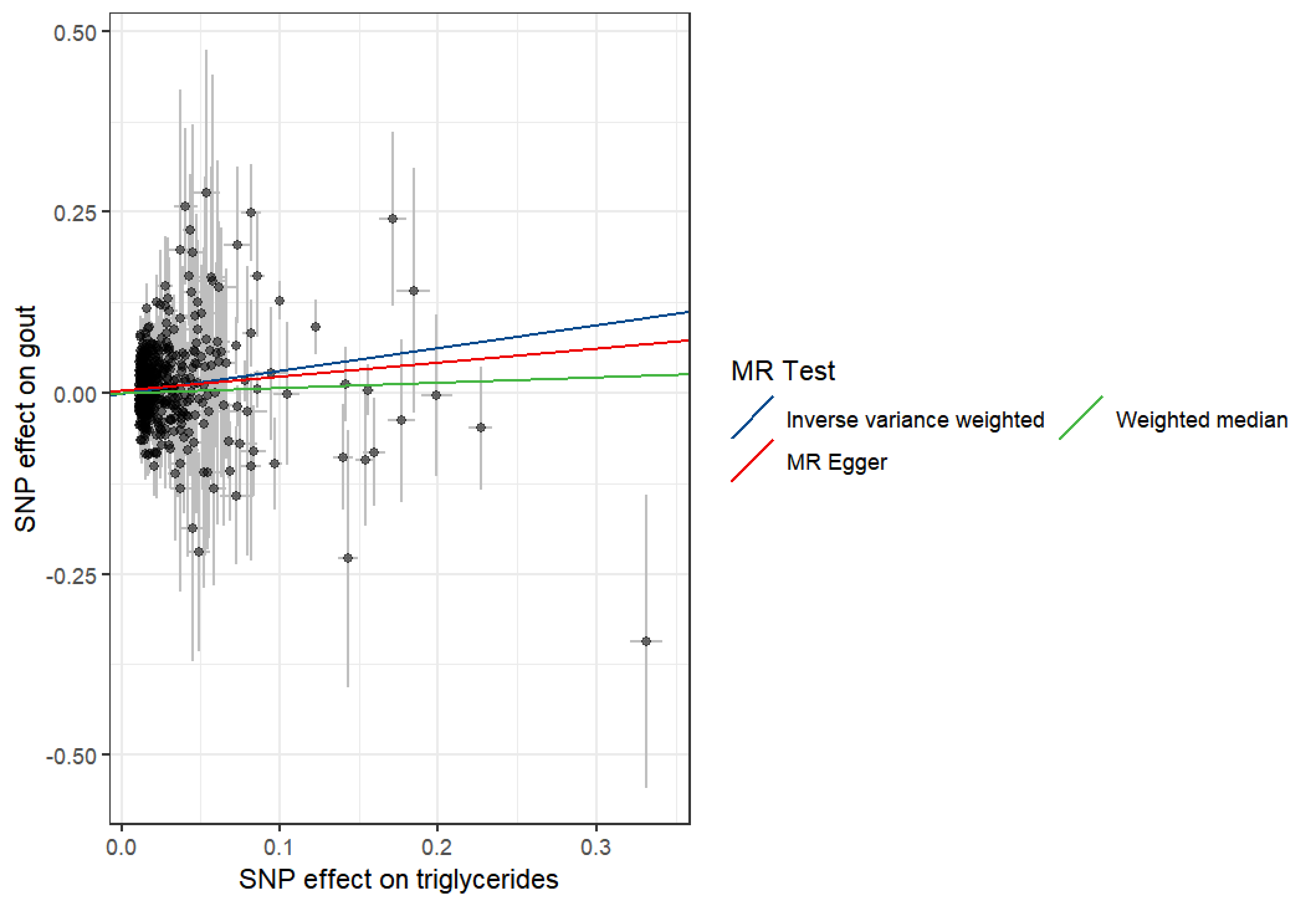

0.50
0.25
0.00
-0.25
-0.50
0.0
0.1
0.2
0.3
SNP effect on gout
SNP effect on triglycerides
MR Test
Inverse variance weighted
MR Egger
Weighted median

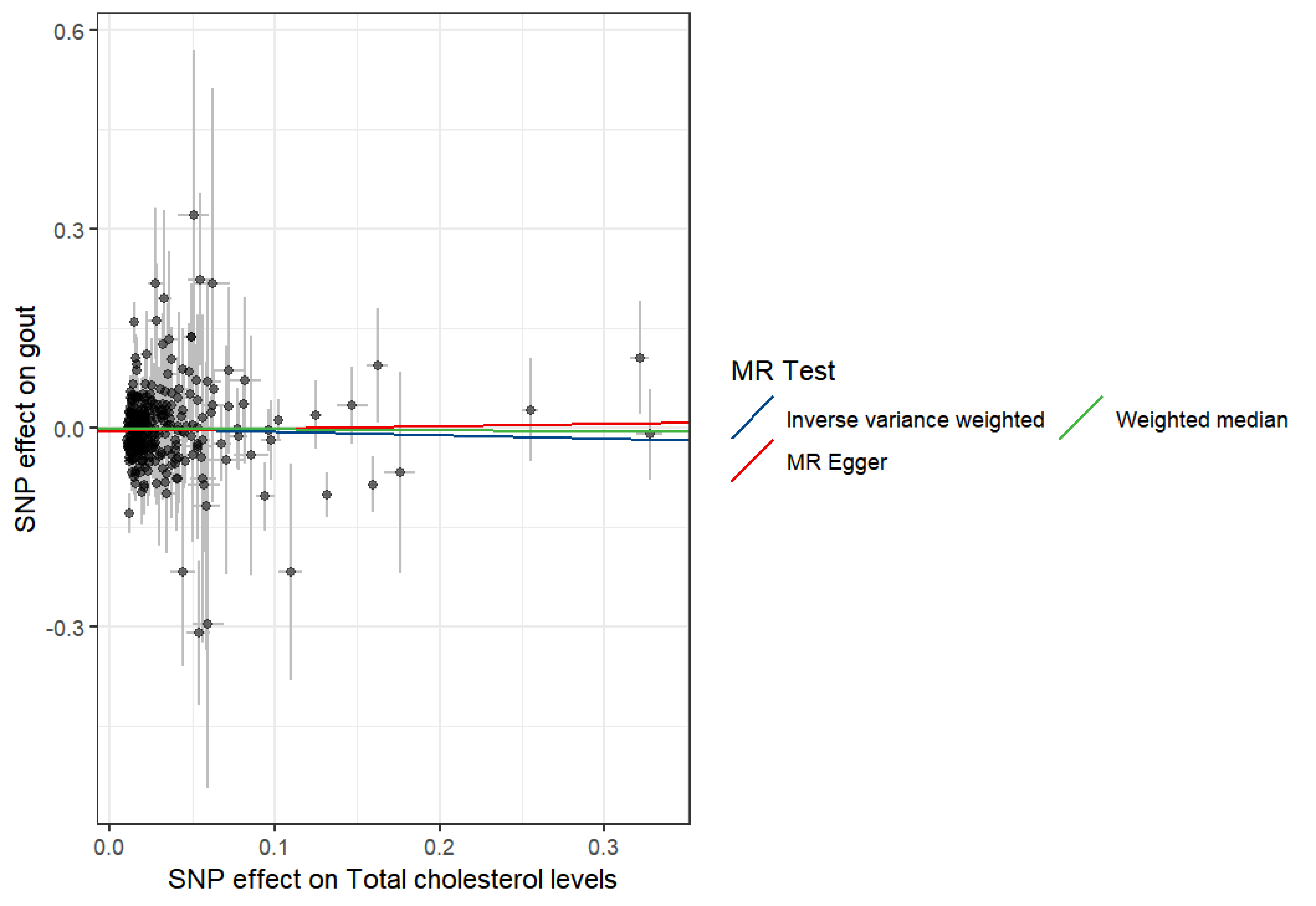

0.6
0.3
0.0
-0.3
SNP effect on gout
0.0
0.1
0.2
0.3
SNP effect on Total cholesterol levels
MR Test
Inverse variance weighted
MR Egger
Weighted median

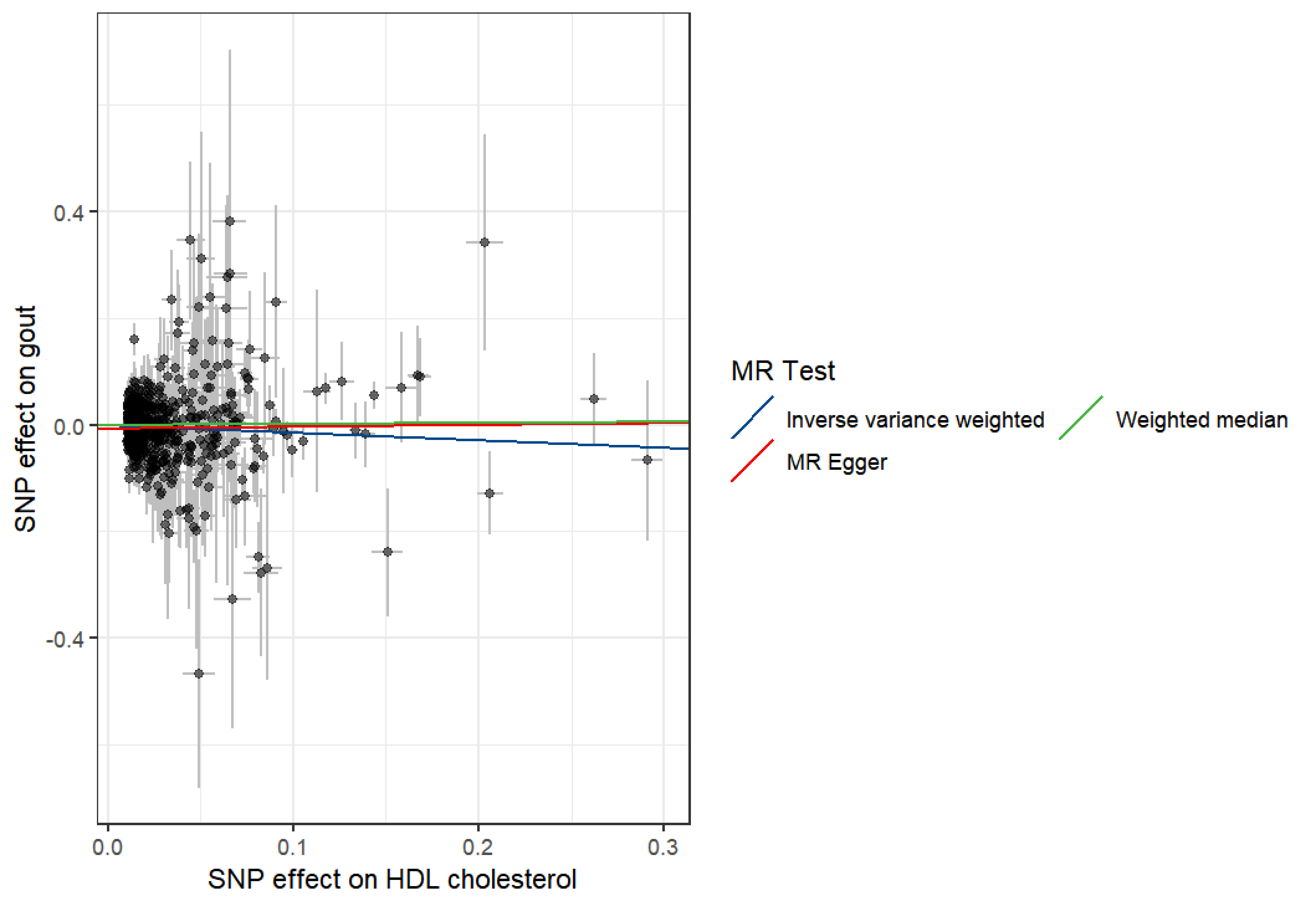

SNP effect on gout
0.4
0.0
-0.4
0.0
0.1
0.2
0.3
SNP effect on HDL cholesterol
MR Test
Inverse variance weighted
MR Egger
Weighted median

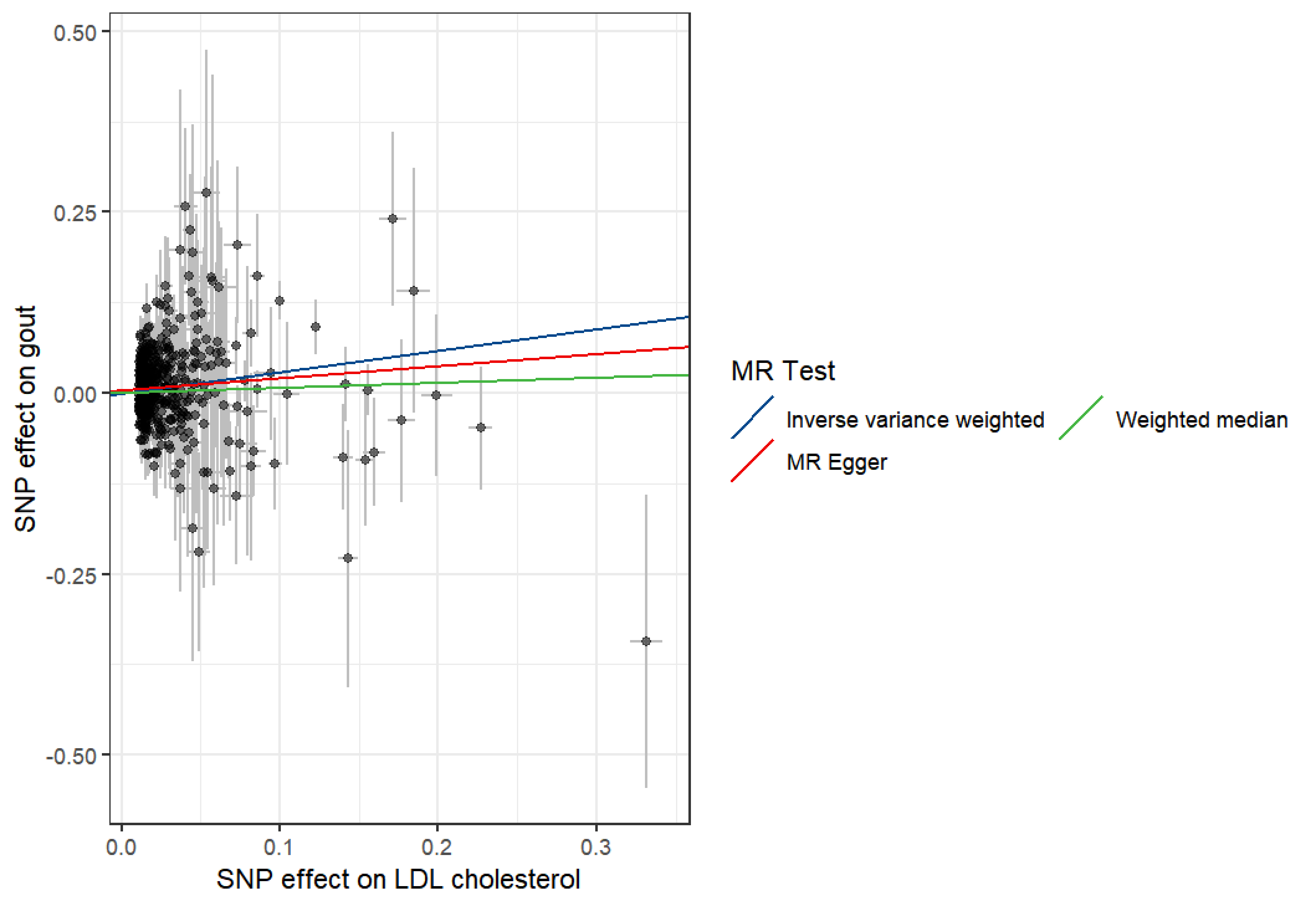
0.50
0.25
0.00
-0.25
-0.50
0.0
0.1
0.2
0.3
SNP effect on gout
SNP effect on LDL cholesterol
MR Test
Inverse variance weighted
MR Egger
Weighted median

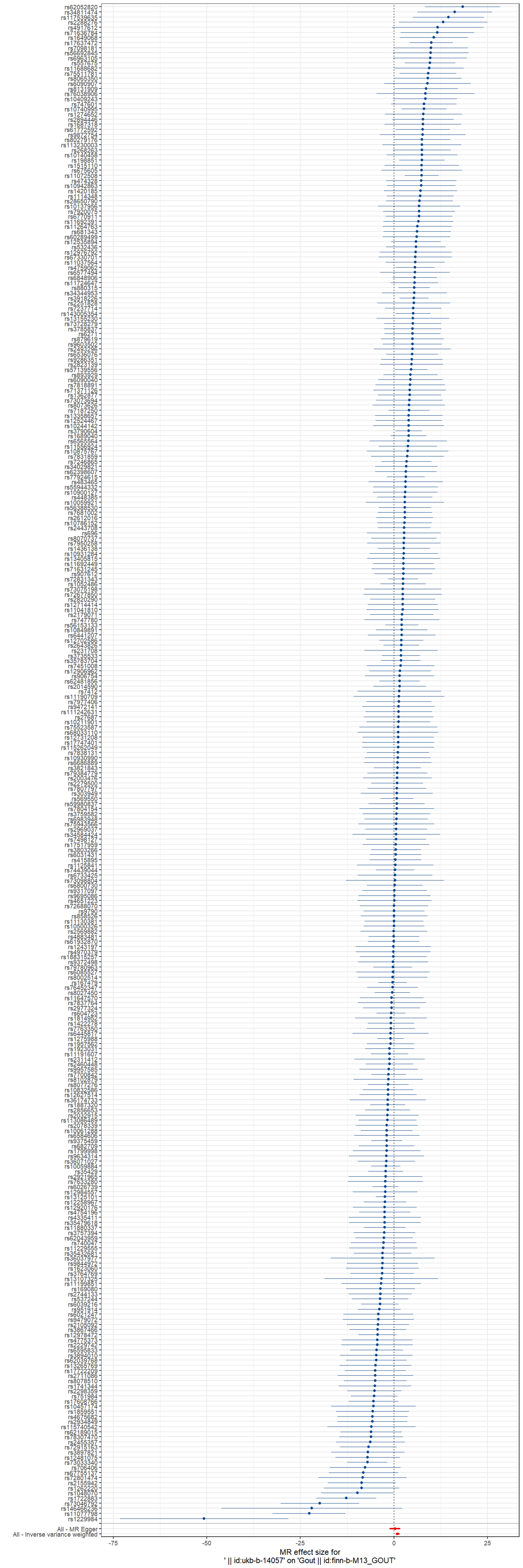
All - MR Egger
All - Inverse variance weighted
-75
-50
-25
0
25
MR effect size for
' || id:ukb-b-14057' on 'Gout || id:finn-b-M13_GOUT'

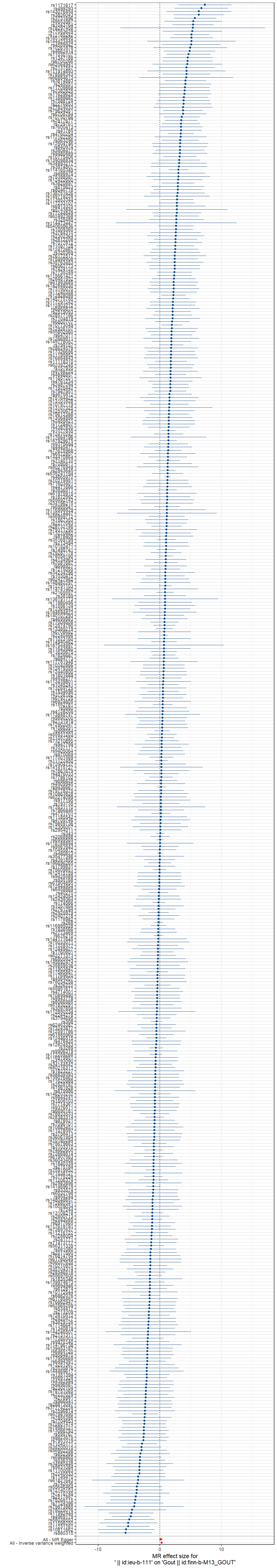

rs1171617
All - MR Egger
All - Inverse variance weighted
-10
0
10
MR effect size for
' || id:ieu-b-111' on 'Gout || id:finn-b-M13_GOUT'

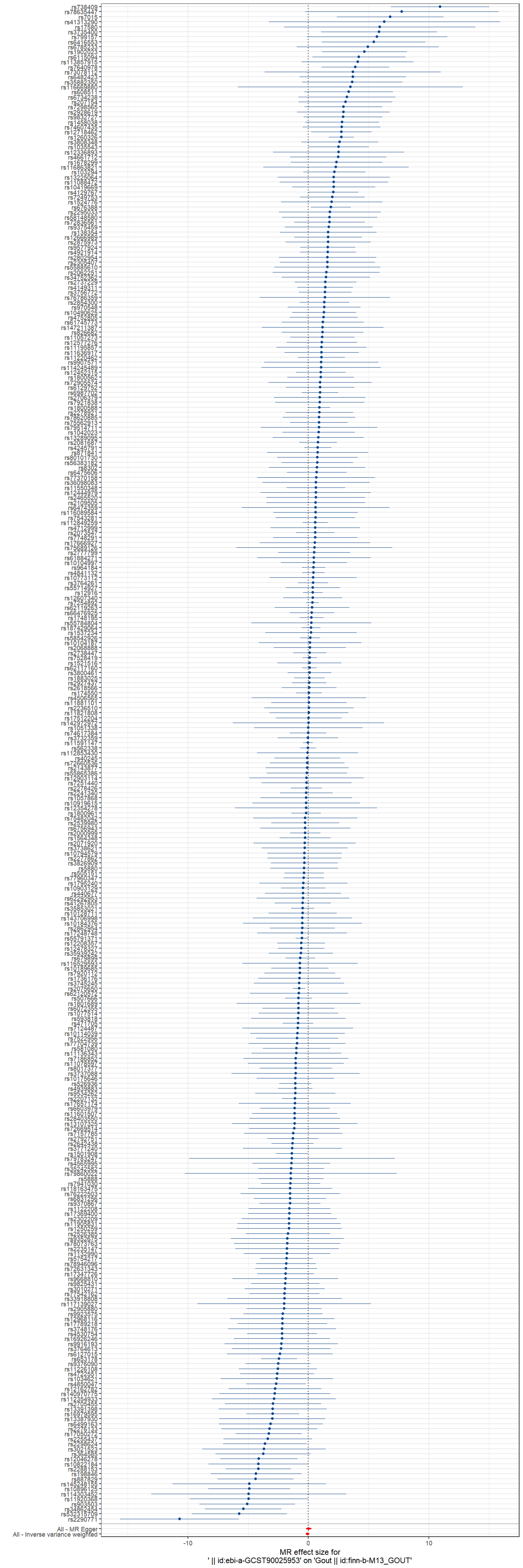

All - MR Egger
All - Inverse variance weighted
-10
0
10
MR effect size for
' || id:ebi-a-GCST90025953' on 'Gout || id:finn-b-M13_GOUT'

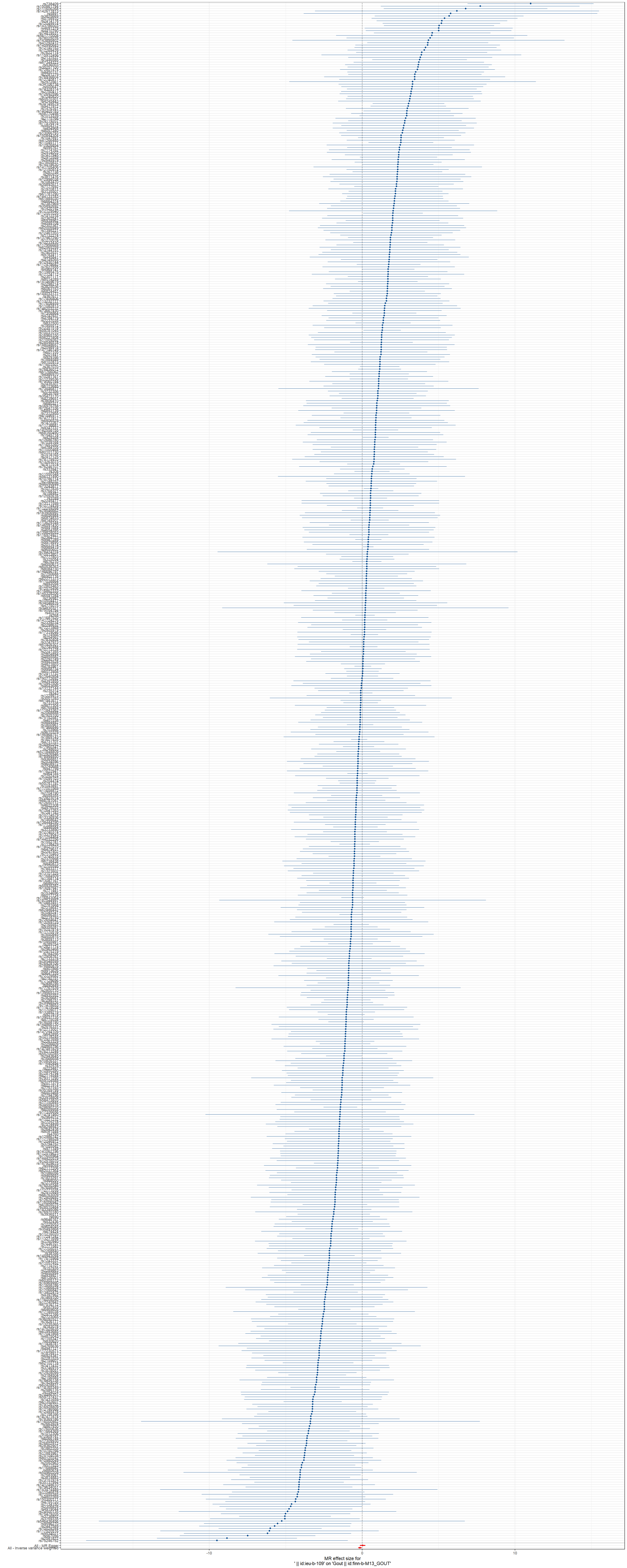
All - MR Egger
All - Inverse variance weighted
-10
0
10
MR effect size for
' || id:ieu-b-109' on 'Gout || id:finn-b-M13_GOUT'

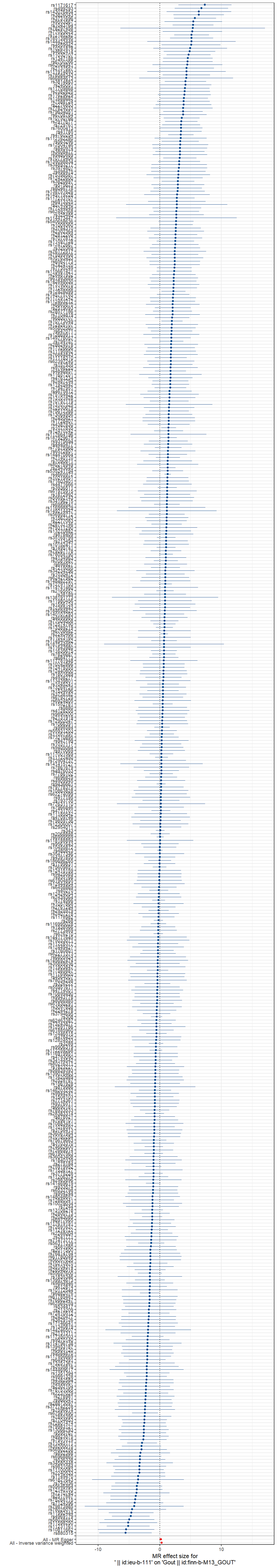
All - MR Egger
All - Inverse variance weighted
-10
0
10
MR effect size for
' || id:ieu-b-111' on 'Gout || id:finn-b-M13_GOUT'

supplementary materials Table 1 Estimates of the effects of the association between gene predicted exposure and gout risk

SNP	Chr	Position	Effect Allele	Other Allele	F	Association with exposure			Association with Gout		
						Beta	SE	*P*	Beta	SE	*P*
**Hypertension**											
rs10059884	5	32832474	A	C	200.461	0.013	0.001	1.70E-45	-0.028	0.026	0.2803
rs10059921	5	87514515	T	G	48.290	-0.012	0.002	3.70E-12	-0.035	0.063	0.5784
rs10061288	5	114389826	G	A	63.802	-0.007	0.001	1.40E-15	0.014	0.025	0.5881
rs10137956	14	39399946	G	A	30.132	-0.006	0.001	4.00E-08	-0.041	0.034	0.2279
rs10140458	14	51153811	T	C	34.221	-0.005	0.001	4.90E-09	-0.040	0.026	0.12
rs10211901	21	45122573	A	G	45.879	0.006	0.001	1.30E-11	0.008	0.027	0.7798
rs10244142	7	130427388	T	C	33.557	0.006	0.001	6.90E-09	0.022	0.027	0.4151
rs10409243	19	10332988	T	C	39.724	-0.006	0.001	2.90E-10	-0.049	0.026	0.0537502
rs10457174	6	97067047	T	C	33.099	0.007	0.001	8.80E-09	-0.036	0.038	0.34
rs1048070	9	14735053	C	T	31.486	0.005	0.001	2.00E-08	-0.050	0.026	0.0490603
rs10500326	16	4918326	T	G	52.947	-0.008	0.001	3.40E-13	0.000	0.032	0.9979
rs1052486	6	31610686	G	A	87.372	0.008	0.001	9.00E-21	0.021	0.026	0.4313
rs10740995	10	18445088	A	G	81.463	0.008	0.001	1.80E-19	0.066	0.025	0.00920195
rs10786152	10	95893514	G	A	58.968	0.007	0.001	1.60E-14	0.020	0.026	0.4458
rs10832586	11	16304089	C	A	82.466	0.010	0.001	1.10E-19	-0.015	0.035	0.6604
rs10849891	12	121911602	G	T	61.192	-0.007	0.001	5.20E-15	-0.015	0.025	0.5556
rs10875767	12	48599257	C	A	38.254	0.007	0.001	6.20E-10	0.025	0.038	0.5064
rs10900127	10	45390103	A	C	40.070	-0.006	0.001	2.50E-10	-0.018	0.026	0.4897
rs10930990	2	182996457	G	C	38.731	-0.006	0.001	4.90E-10	-0.006	0.027	0.8224
rs10931284	2	188211610	T	C	35.661	0.006	0.001	2.30E-09	0.015	0.026	0.57
rs10942863	5	77846554	T	C	36.670	-0.005	0.001	1.40E-09	-0.040	0.026	0.1185
rs11037564	11	43708725	T	C	48.766	0.006	0.001	2.90E-12	0.036	0.025	0.1548
rs11041810	11	8231618	T	G	30.664	-0.005	0.001	3.10E-08	-0.012	0.026	0.6372
rs11072508	15	75062397	T	C	132.968	-0.011	0.001	9.20E-31	-0.082	0.026	0.00133101
rs11077798	17	73953404	C	T	37.794	0.007	0.001	7.90E-10	-0.151	0.033	5.72E-06

rs111242631	19	11299108	A	G	31.319	-0.006	0.001	2.20E-08	-0.008	0.028	0.7805
rs11130381	3	53850005	T	C	49.698	0.006	0.001	1.80E-12	0.000	0.025	0.9951
rs1114348	6	51834280	A	G	38.576	0.006	0.001	5.30E-10	0.039	0.025	0.1237
rs11190709	10	102552663	A	G	35.764	0.009	0.001	2.20E-09	0.012	0.053	0.8274
rs11191607	10	104959188	T	G	108.469	-0.017	0.002	2.10E-25	0.020	0.044	0.645
rs11199851	10	122996928	C	A	37.494	0.007	0.001	9.20E-10	-0.023	0.037	0.5307
rs11229555	11	58408687	T	G	34.253	-0.006	0.001	4.80E-09	0.018	0.029	0.5396
rs1125841	8	30858054	T	C	30.602	-0.005	0.001	3.20E-08	-0.002	0.026	0.9432
rs11264763	1	153948705	T	C	32.660	-0.006	0.001	1.10E-08	-0.036	0.027	0.1829
rs113086489	17	7171356	T	C	51.908	0.007	0.001	5.80E-13	-0.012	0.026	0.652001
rs113230003	19	18460956	A	G	30.980	-0.006	0.001	2.60E-08	-0.043	0.031	0.1614
rs115262049	2	43196694	T	A	37.715	-0.010	0.002	8.20E-10	-0.011	0.048	0.8181
rs11556924	7	129663496	T	C	54.625	-0.007	0.001	1.50E-13	-0.025	0.027	0.3472
rs115740542	6	26123502	C	T	49.864	0.012	0.002	1.60E-12	-0.073	0.073	0.3184
rs11647570	16	4137262	G	A	51.756	-0.011	0.001	6.30E-13	0.006	0.046	0.8943
rs11688682	2	121347612	C	G	35.569	-0.006	0.001	2.50E-09	-0.059	0.030	0.04735
rs11692391	2	113695120	C	T	33.692	-0.006	0.001	6.50E-09	-0.042	0.030	0.1697
rs11692449	2	61704451	C	T	43.818	-0.006	0.001	3.60E-11	-0.016	0.026	0.5378
rs11724647	4	156646340	A	T	65.899	-0.009	0.001	4.70E-16	-0.052	0.031	0.0914197
rs117539635	15	69682916	G	A	51.254	-0.021	0.003	8.10E-13	-0.300	0.100	0.00267997
rs11880337	19	7296452	T	C	60.169	-0.010	0.001	8.70E-15	0.025	0.029	0.378
rs12258967	10	18727959	G	C	117.355	-0.011	0.001	2.40E-27	0.025	0.031	0.4101
rs1229984	4	100239319	C	T	33.784	0.016	0.003	6.20E-09	-0.802	0.181	9.78E-06
rs1243197	10	21970077	C	T	30.143	0.005	0.001	4.00E-08	-0.001	0.025	0.9813
rs12481075	20	10736551	C	T	37.275	-0.006	0.001	1.00E-09	0.043	0.027	0.1085
rs12524467	6	31684790	A	G	33.654	-0.018	0.003	6.60E-09	-0.072	0.082	0.3815
rs12535894	7	27329173	G	C	53.530	0.011	0.001	2.50E-13	0.064	0.037	0.0814798
rs1262220	11	95385613	C	T	31.259	0.005	0.001	2.30E-08	-0.043	0.026	0.0914998
rs12627514	21	44759440	G	C	48.617	0.007	0.001	3.10E-12	-0.011	0.027	0.691301
rs12702586	7	7211940	A	G	73.419	0.012	0.001	1.00E-17	0.024	0.036	0.5043
rs12714414	2	651407	C	T	30.602	-0.007	0.001	3.20E-08	-0.017	0.034	0.6201

rs12731208	1	6676485	G	T	34.102	0.005	0.001	5.20E-09	0.006	0.027	0.8149
rs1274652	14	68676520	G	C	33.410	-0.007	0.001	7.50E-09	-0.058	0.039	0.1315
rs1275988	2	26914364	T	C	233.264	-0.014	0.001	1.20E-52	0.013	0.025	0.611699
rs12906962	15	95312071	C	T	40.329	0.006	0.001	2.10E-10	0.010	0.026	0.6998
rs12920176	16	51761084	C	A	42.821	0.006	0.001	6.00E-11	-0.015	0.026	0.5724
rs12976792	19	7513070	G	A	39.138	-0.007	0.001	3.90E-10	-0.039	0.032	0.234
rs12978472	19	7257990	G	C	208.831	-0.019	0.001	2.50E-47	0.084	0.051	0.09846
rs12984557	19	12519897	A	G	41.537	-0.006	0.001	1.20E-10	0.014	0.026	0.5931
rs13107325	4	103188709	T	C	66.836	-0.014	0.002	3.00E-16	0.046	0.108	0.668399
rs13125101	4	81174592	A	G	462.708	0.021	0.001	1.20E-102	-0.050	0.027	0.0605202
rs13155230	5	57174891	T	C	33.224	0.005	0.001	8.20E-09	0.027	0.027	0.3071
rs13265769	8	82824263	C	T	38.768	0.006	0.001	4.80E-10	-0.028	0.028	0.3158
rs13358657	5	157938070	G	A	40.736	0.008	0.001	1.70E-10	0.034	0.039	0.3852
rs13405815	2	28629343	T	C	32.231	-0.005	0.001	1.40E-08	-0.013	0.026	0.604
rs1362877	1	42485483	T	C	41.847	-0.006	0.001	9.90E-11	-0.025	0.025	0.3295
rs1420185	2	65315903	A	G	33.005	0.005	0.001	9.20E-09	0.039	0.027	0.1563
rs1422278	5	122467417	T	G	50.960	0.009	0.001	9.40E-13	-0.008	0.030	0.8002
rs143005354	10	115741451	A	G	49.329	0.032	0.005	2.20E-12	0.164	0.077	0.0319198
rs1436138	17	75316880	G	A	62.117	-0.007	0.001	3.20E-15	-0.020	0.026	0.4568
rs146466236	17	46735946	G	T	38.784	0.015	0.002	4.70E-10	-0.338	0.191	0.0760309
rs1515110	2	227122216	T	G	30.003	0.005	0.001	4.30E-08	0.038	0.026	0.1433
rs1623060	18	21143183	T	C	31.930	-0.005	0.001	1.60E-08	0.016	0.025	0.5345
rs1649068	10	60304864	C	A	36.425	0.005	0.001	1.60E-09	0.058	0.025	0.0214699
rs167479	19	11526765	T	G	214.386	-0.013	0.001	1.50E-48	0.005	0.026	0.8564
rs1687318	3	14891798	C	T	31.253	-0.006	0.001	2.30E-08	-0.044	0.030	0.1359
rs1689040	12	89978233	T	C	154.847	-0.011	0.001	1.50E-35	-0.044	0.028	0.1113
rs169080	19	4980864	C	T	39.017	-0.006	0.001	4.20E-10	0.021	0.028	0.4464
rs1722883	7	134215403	C	T	48.842	-0.006	0.001	2.80E-12	0.080	0.026	0.00181999
rs1741344	20	4101800	T	C	33.376	0.005	0.001	7.60E-09	-0.027	0.027	0.3049
rs17517959	1	221346974	C	T	40.087	0.006	0.001	2.40E-10	0.003	0.027	0.9036
rs17608766	17	45013271	C	T	60.022	0.010	0.001	9.40E-15	-0.054	0.033	0.1067

rs17637472	17	47461433	A	G	86.957	0.009	0.001	1.10E-20	0.086	0.025	0.000693298
rs17722209	5	148406386	C	T	33.812	-0.007	0.001	6.10E-09	0.033	0.028	0.2309
rs17747401	10	76400164	T	C	33.505	-0.005	0.001	7.10E-09	-0.006	0.027	0.8197
rs1799998	8	143999600	G	A	36.723	-0.005	0.001	1.40E-09	0.011	0.025	0.6718
rs1814952	4	52807245	T	C	33.075	0.006	0.001	8.90E-09	-0.005	0.028	0.8723
rs1859551	14	72165208	G	A	32.184	0.005	0.001	1.40E-08	-0.029	0.026	0.2545
rs188315257	2	25262928	G	T	47.952	-0.011	0.002	4.40E-12	0.002	0.049	0.9652
rs1887320	20	10965998	A	G	139.711	0.011	0.001	3.10E-32	-0.018	0.026	0.4882
rs1923031	20	62483911	C	T	68.197	-0.008	0.001	1.50E-16	0.009	0.026	0.734999
rs1957562	5	157478630	A	G	85.150	0.009	0.001	2.80E-20	-0.009	0.030	0.7757
rs198851	6	26104632	G	T	106.707	-0.013	0.001	5.20E-25	-0.097	0.040	0.0157899
rs2003476	19	18806668	C	T	34.228	-0.005	0.001	4.90E-09	-0.005	0.025	0.8477
rs2014590	3	169146497	T	C	62.279	-0.007	0.001	3.00E-15	-0.011	0.025	0.667899
rs2032915	16	31117413	T	C	40.974	-0.006	0.001	1.50E-10	0.011	0.026	0.6844
rs2078339	12	20161168	G	A	58.695	-0.008	0.001	1.80E-14	0.015	0.033	0.655399
rs2105092	6	134184972	A	G	51.522	-0.007	0.001	7.10E-13	0.030	0.030	0.3134
rs2155942	8	102817883	G	A	48.350	0.007	0.001	3.60E-12	-0.064	0.034	0.0633199
rs2179071	6	22114325	T	C	41.581	0.006	0.001	1.10E-10	0.013	0.025	0.6159
rs2229742	21	16339172	C	G	31.163	0.008	0.001	2.40E-08	-0.037	0.040	0.3586
rs2251828	6	15479363	A	G	32.319	0.006	0.001	1.30E-08	0.031	0.030	0.2885
rs2279500	1	113244433	C	T	65.353	0.010	0.001	6.30E-16	0.009	0.034	0.8019
rs2288276	17	46672154	C	G	30.327	0.008	0.001	3.70E-08	0.107	0.049	0.0286999
rs2298359	21	33671140	C	T	52.521	-0.013	0.002	4.30E-13	0.069	0.049	0.1563
rs2311412	2	69010557	C	T	34.820	-0.005	0.001	3.60E-09	0.006	0.025	0.8034
rs231708	4	2694773	C	G	35.142	-0.006	0.001	3.10E-09	-0.011	0.029	0.6996
rs2443708	3	11545719	C	T	56.873	0.007	0.001	4.60E-14	0.020	0.026	0.4409
rs2455357	5	692887	G	A	31.219	-0.006	0.001	2.30E-08	0.038	0.029	0.1805
rs2460448	16	89708096	A	G	79.419	-0.008	0.001	5.00E-19	0.010	0.026	0.713099
rs2493296	1	3327032	T	C	35.528	0.008	0.001	2.50E-09	0.038	0.041	0.3469
rs2569882	6	1620147	C	T	40.409	-0.006	0.001	2.10E-10	0.000	0.026	0.993
rs2612016	3	53734453	C	G	49.628	0.007	0.001	1.90E-12	0.020	0.026	0.4553

rs2643826	3	27562988	T	C	138.250	0.011	0.001	6.40E-32	0.021	0.026	0.4196
rs268263	2	164954174	A	T	56.431	0.008	0.001	5.80E-14	0.059	0.031	0.0577803
rs2711086	2	159588969	C	A	31.212	0.005	0.001	2.30E-08	-0.026	0.026	0.3346
rs2744133	6	22392260	G	A	42.511	-0.007	0.001	7.00E-11	0.024	0.028	0.3986
rs27687	5	68032422	C	T	36.309	0.006	0.001	1.70E-09	0.008	0.029	0.7925
rs2820290	1	201783682	G	A	40.455	0.006	0.001	2.00E-10	0.014	0.025	0.5927
rs2823139	21	16576783	A	G	39.816	0.006	0.001	2.80E-10	0.029	0.026	0.2729
rs2856653	11	47361084	C	T	92.145	-0.009	0.001	8.10E-22	0.015	0.028	0.5833
rs28650790	5	55861464	T	C	50.588	0.008	0.001	1.10E-12	0.056	0.037	0.1324
rs2894446	8	129410924	T	G	50.643	-0.007	0.001	1.10E-12	-0.052	0.028	0.0618102
rs2921965	10	118445251	C	T	32.453	-0.006	0.001	1.20E-08	0.013	0.029	0.654699
rs2934849	6	166162335	C	T	36.361	-0.006	0.001	1.60E-09	0.032	0.027	0.2237
rs2969037	7	2534392	C	A	53.135	-0.007	0.001	3.10E-13	-0.004	0.030	0.8813
rs2977324	8	76716737	G	T	52.938	0.007	0.001	3.40E-13	-0.005	0.028	0.8725
rs303949	13	72365326	C	A	31.753	0.009	0.002	1.80E-08	0.007	0.043	0.8724
rs34029821	12	53441990	G	A	43.171	0.006	0.001	5.00E-11	0.022	0.028	0.4359
rs34344953	7	156312714	C	T	37.365	0.006	0.001	9.80E-10	0.031	0.025	0.2206
rs34584424	5	68568858	T	C	32.043	0.017	0.003	1.50E-08	0.010	0.099	0.9179
rs34811474	4	25408838	A	G	30.625	-0.006	0.001	3.10E-08	-0.096	0.030	0.001483
rs35429	12	115555867	G	A	143.159	-0.011	0.001	5.40E-33	0.024	0.026	0.3565
rs35432681	3	133966291	C	T	49.378	0.006	0.001	2.10E-12	-0.020	0.025	0.4411
rs35479618	1	153662423	A	G	55.045	0.026	0.003	1.20E-13	-0.063	0.124	0.615
rs35783704	8	105966258	A	G	74.283	-0.013	0.002	6.80E-18	-0.024	0.035	0.487401
rs36037977	20	4426532	A	G	35.130	-0.012	0.002	3.10E-09	0.036	0.085	0.6671
rs36071027	5	158444274	T	C	50.329	-0.007	0.001	1.30E-12	0.013	0.026	0.606301
rs36174733	12	133781163	G	A	33.600	-0.007	0.001	6.80E-09	0.011	0.035	0.750601
rs3735533	7	27245893	C	T	93.666	0.017	0.002	3.70E-22	0.031	0.043	0.4703
rs3757394	7	131010429	T	C	44.105	0.006	0.001	3.10E-11	-0.016	0.026	0.5464
rs3759582	14	103989892	C	A	37.616	-0.008	0.001	8.60E-10	-0.006	0.038	0.8765
rs3764769	2	43732958	T	C	44.612	-0.007	0.001	2.40E-11	0.021	0.030	0.467599
rs3785837	17	59468942	A	G	48.353	0.007	0.001	3.60E-12	0.037	0.029	0.2042

rs3790604	1	113046879	A	C	117.671	0.019	0.002	2.00E-27	0.073	0.034	0.0301301
rs3803266	13	30154349	C	G	52.306	-0.008	0.001	4.70E-13	-0.004	0.026	0.8813
rs3821843	3	53558012	A	G	74.142	0.008	0.001	7.30E-18	0.008	0.027	0.765799
rs3867466	11	111668256	C	A	59.937	0.008	0.001	9.80E-15	-0.033	0.030	0.2652
rs3894010	18	48167237	T	G	32.317	-0.005	0.001	1.30E-08	0.025	0.026	0.3416
rs3897821	1	243420388	G	A	33.653	0.006	0.001	6.60E-09	-0.039	0.028	0.1609
rs3918226	7	150690176	T	C	228.105	0.025	0.002	1.50E-51	0.135	0.050	0.00670301
rs415895	11	9769562	G	C	76.116	0.008	0.001	2.70E-18	0.003	0.029	0.9083
rs4335411	1	249191706	A	G	29.761	0.006	0.001	4.90E-08	-0.015	0.030	0.6182
rs448385	1	25395133	A	G	56.888	0.007	0.001	4.60E-14	0.020	0.026	0.4303
rs4651223	1	184580250	T	C	29.787	0.005	0.001	4.80E-08	0.001	0.027	0.9808
rs4675682	2	208402750	C	T	34.249	0.005	0.001	4.80E-09	-0.030	0.025	0.2311
rs474328	1	193240205	T	C	31.348	0.005	0.001	2.20E-08	0.040	0.026	0.1284
rs4754196	11	107096777	G	A	64.419	0.007	0.001	1.00E-15	-0.018	0.025	0.486
rs4759062	12	54434908	T	C	93.199	-0.009	0.001	4.70E-22	-0.054	0.025	0.0337497
rs4775373	15	61476936	C	T	36.908	-0.006	0.001	1.20E-09	0.025	0.027	0.3586
rs483465	3	136047977	G	A	45.307	0.007	0.001	1.70E-11	0.022	0.035	0.5348
rs4883481	12	50574311	C	T	66.601	-0.008	0.001	3.30E-16	0.000	0.026	0.9867
rs4917612	10	96591284	G	C	31.102	-0.007	0.001	2.40E-08	-0.087	0.047	0.0612802
rs4970379	1	875770	G	A	30.281	0.005	0.001	3.70E-08	-0.001	0.026	0.9796
rs532436	9	136149830	A	G	44.546	-0.008	0.001	2.50E-11	-0.045	0.031	0.1505
rs537244	15	41469313	T	G	57.431	-0.007	0.001	3.50E-14	0.025	0.027	0.3367
rs557675	11	65566719	G	T	69.367	-0.007	0.001	8.20E-17	-0.073	0.026	0.00505103
rs55944332	2	145726621	G	A	38.096	0.007	0.001	6.70E-10	0.020	0.029	0.482
rs56153133	1	11885647	G	A	169.146	-0.016	0.001	1.10E-38	-0.034	0.036	0.3473
rs56388530	4	106910958	T	C	81.267	0.009	0.001	2.00E-19	0.028	0.034	0.4116
rs56692845	12	12887811	T	C	34.567	-0.006	0.001	4.10E-09	-0.058	0.030	0.0553006
rs569550	11	1887068	G	T	151.756	0.011	0.001	7.20E-35	0.009	0.026	0.73
rs57139556	6	150998511	G	A	110.818	-0.018	0.002	6.50E-26	-0.084	0.041	0.0383902
rs59980837	1	115827266	T	G	78.758	0.030	0.003	7.00E-19	0.023	0.116	0.8433
rs6021247	20	50108980	A	G	35.837	0.005	0.001	2.10E-09	-0.023	0.026	0.3791

rs6026739	20	57739469	T	A	186.895	0.019	0.001	1.50E-42	-0.044	0.034	0.1914
rs60289499	17	43218677	A	G	36.408	0.006	0.001	1.60E-09	0.037	0.028	0.184
rs6031431	20	42795152	G	A	64.165	0.007	0.001	1.10E-15	0.003	0.025	0.8921
rs6039216	20	8622480	C	T	121.991	-0.010	0.001	2.30E-28	0.039	0.026	0.1437
rs604723	11	100610546	C	T	205.636	0.015	0.001	1.20E-46	-0.011	0.029	0.711001
rs6085527	20	6366990	G	A	32.811	0.005	0.001	1.00E-08	-0.002	0.026	0.9534
rs6090040	20	62692060	C	A	41.575	-0.006	0.001	1.10E-10	-0.026	0.025	0.3114
rs6090907	20	47410231	A	G	35.471	-0.008	0.001	2.60E-09	-0.067	0.044	0.1285
rs61772592	1	56979681	G	A	44.445	0.009	0.001	2.60E-11	0.070	0.034	0.03748
rs61932870	12	51502156	T	G	40.287	-0.017	0.003	2.20E-10	0.002	0.060	0.9796
rs62039768	16	51560761	A	C	50.500	0.011	0.002	1.20E-12	-0.052	0.045	0.2513
rs62043959	16	81525204	C	A	56.023	-0.008	0.001	7.20E-14	0.020	0.030	0.5025
rs62052820	16	69575238	A	G	31.542	0.006	0.001	2.00E-08	0.111	0.031	0.000336899
rs62189015	2	162583504	C	T	37.154	-0.009	0.002	1.10E-09	0.056	0.038	0.1418
rs62398607	6	39145523	C	T	39.515	-0.007	0.001	3.30E-10	-0.022	0.029	0.442
rs62481856	7	106414069	A	G	78.309	0.010	0.001	8.80E-19	0.015	0.028	0.581
rs6271	9	136522274	T	C	59.875	-0.013	0.002	1.00E-14	-0.066	0.051	0.1906
rs6441207	3	158282459	T	C	38.283	0.006	0.001	6.10E-10	0.012	0.026	0.6528
rs6445817	3	56748186	A	C	32.357	-0.005	0.001	1.30E-08	0.005	0.028	0.8622
rs6536076	4	156420605	T	A	56.252	-0.007	0.001	6.40E-14	-0.036	0.026	0.1648
rs6565564	17	79381917	C	G	32.729	-0.011	0.002	1.10E-08	-0.043	0.059	0.467599
rs6577494	1	8576925	T	C	38.953	0.006	0.001	4.30E-10	0.033	0.028	0.2378
rs6584606	10	106277309	A	C	43.789	0.024	0.004	3.70E-11	-0.046	0.107	0.6674
rs6595833	5	127843208	T	C	64.725	0.007	0.001	8.60E-16	-0.034	0.027	0.1999
rs6686889	1	25047459	T	C	34.844	0.006	0.001	3.60E-09	0.006	0.028	0.8275
rs67330701	11	69079707	T	C	36.004	-0.010	0.002	2.00E-09	-0.055	0.048	0.2506
rs6733425	2	43399561	T	A	29.910	-0.006	0.001	4.50E-08	-0.002	0.028	0.9484
rs675605	13	110873556	C	G	30.620	-0.005	0.001	3.10E-08	-0.041	0.030	0.1773
rs6770911	3	169295764	C	A	43.595	0.007	0.001	4.00E-11	0.044	0.030	0.1414
rs67755137	7	74108135	A	G	43.334	0.007	0.001	4.60E-11	-0.061	0.035	0.0854594
rs6800730	3	48174210	G	A	70.482	0.008	0.001	4.60E-17	0.002	0.030	0.945

rs68033110	18	57914679	A	G	31.599	0.006	0.001	1.90E-08	0.007	0.033	0.835
rs681343	19	49206462	T	C	39.933	0.006	0.001	2.60E-10	0.035	0.026	0.178
rs682709	13	22294082	G	C	50.850	0.007	0.001	1.00E-12	-0.013	0.026	0.6067
rs6848906	4	111386198	C	T	71.987	-0.011	0.001	2.20E-17	-0.059	0.032	0.0633797
rs696	14	35871093	T	C	30.815	0.005	0.001	2.80E-08	0.014	0.026	0.5902
rs6963105	7	75097488	G	A	31.302	0.005	0.001	2.20E-08	0.049	0.025	0.05319
rs6983948	8	95990283	T	C	42.810	-0.006	0.001	6.00E-11	-0.004	0.025	0.8738
rs706406	1	56627288	A	C	37.396	0.006	0.001	9.60E-10	-0.044	0.027	0.1122
rs7098181	10	65027143	T	G	32.131	-0.005	0.001	1.40E-08	-0.051	0.026	0.0481704
rs71371126	17	28150359	A	G	31.417	0.005	0.001	2.10E-08	0.022	0.026	0.3942
rs71631245	3	183957218	T	C	32.427	0.016	0.003	1.20E-08	0.042	0.071	0.558399
rs71636784	1	27169200	G	T	32.985	0.007	0.001	9.30E-09	0.081	0.035	0.0203301
rs7187250	16	53810686	A	C	100.174	0.009	0.001	1.40E-23	0.037	0.026	0.1499
rs7237714	18	1910859	G	A	43.052	0.009	0.001	5.30E-11	0.045	0.034	0.1826
rs7246865	19	17219105	A	G	77.753	0.009	0.001	1.20E-18	0.031	0.031	0.3234
rs72677850	14	50849397	A	G	47.886	-0.023	0.003	4.50E-12	-0.056	0.125	0.6568
rs72688070	8	81393697	T	C	51.701	-0.009	0.001	6.50E-13	-0.001	0.040	0.9892
rs72801474	5	132444128	A	G	35.373	-0.009	0.002	2.70E-09	0.077	0.055	0.1614
rs72831343	10	63515681	G	T	249.706	-0.020	0.001	3.00E-56	-0.050	0.041	0.2289
rs72915163	18	48792829	T	C	58.881	0.008	0.001	1.70E-14	-0.054	0.031	0.0791498
rs73033340	7	1195692	G	A	40.703	-0.016	0.003	1.80E-10	0.116	0.045	0.00964095
rs73046792	19	49605705	A	G	38.803	-0.008	0.001	4.70E-10	0.150	0.041	0.000218499
rs73073694	12	20360328	G	A	50.323	-0.007	0.001	1.30E-12	-0.028	0.030	0.3556
rs73075198	12	20705540	T	C	29.801	-0.005	0.001	4.80E-08	-0.013	0.027	0.6457
rs73098804	4	17865865	A	T	37.404	0.008	0.001	9.60E-10	0.002	0.055	0.9682
rs73728279	7	151411494	T	G	60.799	0.008	0.001	6.30E-15	0.039	0.030	0.1943
rs740047	7	92230162	T	C	34.407	0.007	0.001	4.50E-09	-0.018	0.029	0.5325
rs7412	19	45412079	T	C	34.851	-0.010	0.002	3.60E-09	-0.014	0.056	0.7999
rs74439044	17	7781019	C	T	77.317	0.013	0.002	1.50E-18	0.005	0.035	0.8963
rs7451008	6	20673880	C	T	32.267	0.006	0.001	1.30E-08	0.010	0.027	0.7006
rs747601	11	13274553	T	A	39.540	0.006	0.001	3.20E-10	0.051	0.028	0.0708598

rs747780	15	42170506	T	C	30.452	0.005	0.001	3.40E-08	0.011	0.025	0.6772
rs7498127	16	49774755	A	G	46.401	-0.006	0.001	9.60E-12	-0.004	0.025	0.8858
rs751984	11	61278246	C	T	55.419	-0.011	0.001	9.70E-14	0.056	0.034	0.0972501
rs75511781	7	131323710	G	A	30.998	0.012	0.002	2.60E-08	0.112	0.048	0.0193201
rs75523587	10	134413583	A	T	34.501	0.007	0.001	4.30E-09	0.008	0.036	0.8224
rs75943566	3	160265817	C	T	30.771	-0.006	0.001	2.90E-08	-0.004	0.030	0.904
rs76038906	9	113250200	T	G	47.762	0.017	0.002	4.80E-12	0.143	0.113	0.2056
rs7633280	3	168581491	G	A	30.849	0.005	0.001	2.80E-08	-0.012	0.027	0.6578
rs76452347	9	35906471	T	C	68.968	-0.010	0.001	1.00E-16	0.004	0.033	0.9151
rs7681002	4	103895223	G	A	57.715	-0.007	0.001	3.00E-14	-0.020	0.025	0.4336
rs7700842	5	157824183	C	T	145.779	-0.011	0.001	1.50E-33	0.016	0.027	0.5427
rs7763350	6	43349308	C	A	75.253	0.008	0.001	4.10E-18	-0.007	0.027	0.8017
rs77924615	16	20392332	A	G	109.597	-0.012	0.001	1.20E-25	-0.038	0.031	0.2171
rs7804154	7	131244055	A	G	30.527	-0.006	0.001	3.30E-08	-0.004	0.029	0.8823
rs7807797	7	139462493	G	A	45.854	-0.008	0.001	1.30E-11	-0.006	0.031	0.8403
rs7818891	8	110042172	A	G	34.968	-0.005	0.001	3.40E-09	-0.023	0.025	0.3555
rs78307470	3	168857633	G	A	46.050	-0.012	0.002	1.20E-11	0.073	0.051	0.1564
rs7831859	8	101676642	C	T	31.815	-0.005	0.001	1.70E-08	-0.019	0.026	0.4672
rs7837764	8	23375702	C	G	36.958	0.006	0.001	1.20E-09	-0.003	0.026	0.8975
rs7838131	8	11596163	A	G	71.796	-0.008	0.001	2.40E-17	-0.008	0.033	0.7973
rs7920075	10	18345137	T	C	32.889	-0.005	0.001	9.80E-09	-0.035	0.026	0.1696
rs79384779	20	31214944	T	C	37.602	0.008	0.001	8.70E-10	0.007	0.032	0.8238
rs7950258	11	48481492	A	C	45.847	-0.008	0.001	1.30E-11	-0.020	0.038	0.5947
rs7977406	12	115359424	A	G	48.319	-0.007	0.001	3.60E-12	-0.009	0.030	0.7625
rs79780963	10	104952499	T	C	106.947	-0.017	0.002	4.60E-25	0.005	0.046	0.9219
rs8002514	13	115002305	A	G	34.935	0.006	0.001	3.40E-09	-0.002	0.030	0.946
rs8027450	15	91418394	T	C	149.425	0.012	0.001	2.30E-34	-0.005	0.029	0.8545
rs80279176	6	26185403	G	T	40.760	-0.014	0.002	1.70E-10	-0.104	0.053	0.0509296
rs8065350	17	1957597	A	G	30.254	0.006	0.001	3.80E-08	0.053	0.027	0.0512802
rs8070737	17	3981066	T	G	38.208	0.007	0.001	6.40E-10	0.019	0.032	0.5434
rs8073626	17	76790279	T	C	34.378	-0.005	0.001	4.50E-09	-0.021	0.026	0.4136

rs8077276	17	61547562	A	G	99.695	-0.009	0.001	1.80E-23	0.014	0.026	0.584501
rs8078510	17	47045862	A	G	45.323	-0.007	0.001	1.70E-11	0.034	0.029	0.2417
rs8102879	19	1832317	A	G	34.701	-0.007	0.001	3.80E-09	0.010	0.031	0.7527
rs8131909	21	37689720	G	C	31.835	0.008	0.001	1.70E-08	0.066	0.033	0.0457204
rs858526	17	7499382	G	A	36.650	-0.006	0.001	1.40E-09	0.000	0.025	0.9948
rs879619	16	4015046	A	G	36.092	-0.008	0.001	1.90E-09	-0.039	0.034	0.2482
rs880315	1	10796866	C	T	158.572	0.012	0.001	2.30E-36	0.066	0.026	0.0108901
rs893929	4	144187380	A	G	57.663	-0.007	0.001	3.10E-14	-0.031	0.025	0.2236
rs906754	5	63820119	A	G	39.753	-0.007	0.001	2.90E-10	-0.010	0.031	0.7399
rs907612	11	1874221	T	C	50.503	0.007	0.001	1.20E-12	0.017	0.026	0.5218
rs9286351	4	138441530	G	A	44.092	0.006	0.001	3.10E-11	0.029	0.025	0.2524
rs9317097	13	23170374	T	C	37.135	0.007	0.001	1.10E-09	0.001	0.031	0.9684
rs9372498	6	118601980	A	T	32.750	0.010	0.002	1.00E-08	-0.002	0.046	0.9612
rs9375459	6	127147704	T	C	177.662	0.012	0.001	1.60E-40	-0.023	0.025	0.3603
rs9472141	6	43818724	C	T	30.205	-0.005	0.001	3.90E-08	-0.007	0.026	0.7948
rs9479072	6	151890789	T	C	34.043	-0.005	0.001	5.40E-09	0.022	0.026	0.3894
rs951914	8	25878995	C	G	81.394	0.009	0.001	1.80E-19	-0.035	0.027	0.1819
rs9603502	13	32245813	A	C	49.965	0.006	0.001	1.60E-12	0.032	0.026	0.2188
rs9634314	12	110406386	A	G	34.330	-0.010	0.002	4.70E-09	0.020	0.051	0.6955
rs9695086	9	140232124	A	G	30.699	-0.007	0.001	3.00E-08	-0.001	0.033	0.9726
rs9790	16	3586230	T	C	48.063	0.008	0.001	4.10E-12	0.000	0.032	0.9893
rs9844972	3	150097635	C	G	42.954	0.012	0.002	5.60E-11	-0.036	0.056	0.5264
rs9872754	3	138117985	T	C	32.448	0.007	0.001	1.20E-08	0.054	0.041	0.19
rs9957585	18	42071590	T	C	53.193	-0.007	0.001	3.00E-13	0.010	0.028	0.7252

SNP	Chr	Position	Effect Allele	Other Allele	F	Association with exposure			Association with Gout		
						Beta	SE	*P*	Beta	SE	*P*
**TG**											
rs10029034	4	15096680	T	C	31.031	0.012	0.002	2.50E-08	-0.014	0.029	0.628901
rs1009360	2	65276049	C	T	84.741	-0.018	0.002	3.40E-20	-0.049	0.026	0.0550402
rs10152471	15	101890913	A	G	43.596	-0.014	0.002	4.00E-11	-0.014	0.026	0.580801
rs10172544	2	85788270	A	C	31.040	-0.011	0.002	2.50E-08	0.018	0.026	0.4825
rs10180284	2	50716016	T	C	34.361	-0.012	0.002	4.60E-09	0.011	0.025	0.674599
rs10210970	2	28646847	T	C	63.089	0.023	0.003	2.00E-15	-0.035	0.033	0.2789
rs10219951	13	114522295	T	C	37.875	0.019	0.003	7.50E-10	0.024	0.033	0.4628
rs10242866	7	17920613	T	C	60.599	0.016	0.002	7.00E-15	0.010	0.028	0.7061
rs10261878	7	25950545	C	A	31.648	-0.025	0.004	1.80E-08	-0.122	0.075	0.1056
rs1030472	5	157994544	G	A	86.743	0.023	0.002	1.20E-20	0.021	0.036	0.5667
rs1037117	15	102068658	A	G	56.478	0.017	0.002	5.70E-14	0.006	0.028	0.8407
rs1043764	12	122630915	T	C	43.411	-0.017	0.003	4.40E-11	-0.090	0.028	0.00132501
rs1045241	5	118729286	T	C	84.857	-0.021	0.002	3.20E-20	-0.011	0.027	0.679599
rs10455872	6	161010118	G	A	145.056	-0.044	0.004	2.10E-33	-0.001	0.061	0.9832
rs1057868	7	75615006	T	C	31.852	0.012	0.002	1.70E-08	-0.002	0.026	0.9307
rs1066391	12	67716703	G	A	32.783	-0.012	0.002	1.00E-08	-0.005	0.026	0.8479
rs10750766	11	65473798	A	C	78.540	0.019	0.002	7.80E-19	0.069	0.029	0.01595
rs10773049	12	124506631	C	T	203.256	-0.029	0.002	4.10E-46	-0.058	0.026	0.02374
rs10775406	17	46197755	G	A	78.886	0.021	0.002	6.60E-19	0.067	0.029	0.0184599
rs1077835	15	58723426	G	A	388.053	0.047	0.002	2.20E-86	0.068	0.029	0.0184999
rs10797119	9	92202495	C	T	61.425	0.016	0.002	4.60E-15	0.024	0.025	0.3384
rs10811662	9	22134253	A	G	34.354	-0.015	0.003	4.60E-09	0.084	0.036	0.0196598
rs10863828	1	210592392	G	T	31.102	-0.013	0.002	2.40E-08	-0.003	0.029	0.9248
rs10883451	10	101924418	C	T	29.756	0.011	0.002	4.90E-08	-0.009	0.026	0.7199
rs10899490	11	78105879	T	C	39.874	-0.017	0.003	2.70E-10	0.007	0.032	0.8314
rs11023160	11	14264225	A	T	30.434	0.012	0.002	3.50E-08	0.009	0.026	0.734

rs11030107	11	27694835	G	A	50.521	0.016	0.002	1.20E-12	0.075	0.029	0.00962698
rs11045171	12	20470199	G	A	99.322	-0.025	0.003	2.10E-23	0.072	0.031	0.0193602
rs11057397	12	124419728	T	C	168.279	-0.027	0.002	1.80E-38	-0.010	0.028	0.7309
rs11078597	17	1618363	C	T	56.462	0.019	0.003	5.70E-14	-0.017	0.032	0.6084
rs11097128	4	88068837	A	G	40.028	-0.021	0.003	2.50E-10	-0.053	0.032	0.1026
rs11100083	4	157682598	C	T	45.493	-0.016	0.002	1.50E-11	0.053	0.032	0.1012
rs11118310	1	219637671	T	A	91.446	0.019	0.002	1.10E-21	0.035	0.026	0.1753
rs11122450	1	230301811	G	T	559.145	-0.048	0.002	1.30E-123	-0.014	0.026	0.591701
rs11185542	3	196187927	C	G	31.883	-0.013	0.002	1.60E-08	-0.002	0.028	0.9364
rs111914893	7	74002323	T	C	36.563	0.028	0.005	1.50E-09	0.120	0.090	0.1821
rs11195952	10	114173554	A	G	57.023	-0.023	0.003	4.30E-14	-0.043	0.049	0.3775
rs11206374	1	40048009	A	G	110.937	0.025	0.002	6.10E-26	-0.025	0.031	0.4104
rs11216122	11	116609540	T	G	89.898	-0.043	0.004	2.50E-21	0.055	0.057	0.3324
rs112162280	17	43194413	T	C	33.160	-0.013	0.002	8.50E-09	-0.043	0.027	0.1134
rs112259268	17	41874745	A	C	540.802	0.140	0.006	1.30E-119	-0.089	0.073	0.2218
rs11228377	11	68603346	C	T	58.226	-0.015	0.002	2.30E-14	0.004	0.026	0.876
rs11231161	11	62378221	G	A	65.951	0.017	0.002	4.60E-16	-0.085	0.030	0.00403097
rs112403212	12	125308682	T	C	37.206	0.018	0.003	1.10E-09	0.037	0.033	0.2515
rs11240358	1	205070573	A	G	44.674	0.014	0.002	2.30E-11	0.031	0.026	0.2453
rs112850234	4	3407863	A	G	32.942	-0.055	0.010	9.50E-09	0.025	0.175	0.8857
rs113439801	12	62838230	T	C	42.833	-0.017	0.003	6.00E-11	-0.010	0.039	0.7983
rs113848747	8	126534530	T	C	42.851	0.043	0.007	5.90E-11	0.020	0.135	0.8829
rs113953344	6	30774416	A	C	52.019	0.022	0.003	5.50E-13	0.062	0.030	0.0384999

rs114165349	1	27021913	C	G	151.997	0.082	0.007	6.30E-35	0.249	0.067	0.000189601
rs114473447	6	32518488	A	G	49.037	0.057	0.008	2.50E-12	0.154	0.286	0.589599
rs114741460	6	32596835	T	C	156.896	0.063	0.005	5.40E-36	0.056	0.173	0.7465
rs114796738	3	52757046	T	C	30.641	0.025	0.004	3.10E-08	-0.049	0.070	0.4813
rs114940462	2	27861618	T	G	159.620	-0.061	0.005	1.40E-36	-0.043	0.065	0.5046
rs1149470	8	64608119	A	T	35.589	-0.014	0.002	2.40E-09	0.049	0.031	0.1201
rs115661421	2	26945012	G	A	32.933	0.062	0.011	9.50E-09	0.146	0.090	0.1034
rs11598250	10	94416521	C	G	30.254	0.013	0.002	3.80E-08	-0.066	0.031	0.0331497
rs11620989	14	69746345	G	A	30.776	0.011	0.002	2.90E-08	-0.008	0.026	0.749701
rs11629629	15	43403415	A	G	35.763	0.012	0.002	2.20E-09	0.050	0.027	0.0686894
rs11635675	15	63793238	G	T	132.053	0.024	0.002	1.50E-30	0.017	0.026	0.5208
rs11643980	16	85275894	G	T	30.694	-0.014	0.003	3.00E-08	-0.010	0.031	0.7545
rs11657201	17	599924	G	A	31.258	0.013	0.002	2.30E-08	0.023	0.034	0.501
rs11688682	2	121347612	C	G	41.552	-0.015	0.002	1.10E-10	-0.059	0.030	0.04735
rs116919951	8	19933689	C	T	31.917	0.047	0.008	1.60E-08	-0.030	0.086	0.723801
rs116995028	11	116999072	T	C	400.245	0.177	0.009	4.90E-89	-0.039	0.113	0.733199
rs116998829	8	19520669	G	A	33.653	-0.029	0.005	6.60E-09	-0.034	0.093	0.715801
rs117001569	8	19574920	G	C	75.575	-0.094	0.011	3.50E-18	-0.027	0.092	0.7703
rs11709868	3	142648844	T	G	42.818	-0.014	0.002	6.00E-11	-0.058	0.029	0.0456405
rs1171617	10	61467182	T	G	45.073	0.016	0.002	1.90E-11	0.116	0.035	0.000865705
rs11723310	12	4328521	A	G	72.305	-0.073	0.009	1.80E-17	-0.204	0.108	0.0583795

7											
rs117291242	11	380315	T	C	31.795	0.030	0.005	1.70E-08	0.064	0.057	0.2585
rs117350303	8	126466408	G	A	46.939	-0.073	0.011	7.30E-12	0.142	0.095	0.1374
rs117619191	11	117248283	C	T	138.971	0.082	0.007	4.50E-32	-0.102	0.129	0.4286
rs117664196	8	126673909	C	T	39.445	-0.038	0.006	3.40E-10	-0.055	0.120	0.6498
rs11769620	7	72995749	G	A	33.764	0.023	0.004	6.20E-09	-0.008	0.054	0.8769
rs117781948	8	126351023	A	G	43.997	0.057	0.009	3.30E-11	0.038	0.159	0.809
rs1178982	7	72851167	C	T	131.139	-0.043	0.004	2.30E-30	0.009	0.057	0.882
rs117956669	8	19847645	G	T	34.585	0.054	0.009	4.10E-09	-0.109	0.107	0.3066
rs118188894	11	57142624	C	T	35.442	0.019	0.003	2.60E-09	0.001	0.053	0.9821
rs11903847	2	25592918	C	T	39.468	-0.013	0.002	3.30E-10	0.004	0.031	0.8872
rs11925442	3	170692496	C	T	62.994	0.024	0.003	2.10E-15	0.041	0.037	0.2668
rs11980456	7	29319249	A	G	31.366	0.012	0.002	2.10E-08	0.011	0.028	0.701799
rs11992444	8	25464690	T	G	49.869	-0.014	0.002	1.60E-12	0.065	0.026	0.0124099
rs12140153	1	62579891	T	G	40.163	-0.022	0.003	2.30E-10	0.082	0.047	0.0803804
rs12185242	17	47407071	C	A	76.873	0.018	0.002	1.80E-18	0.091	0.025	0.000316403
rs12306007	12	47184042	T	C	30.131	0.016	0.003	4.00E-08	0.002	0.036	0.9585
rs12369443	12	20582651	G	A	30.355	-0.014	0.003	3.60E-08	-0.012	0.029	0.6853
rs12415159	10	113978850	G	A	55.819	0.021	0.003	7.90E-14	-0.001	0.038	0.9822
rs12422600	12	54429385	A	G	36.668	-0.013	0.002	1.40E-09	-0.038	0.026	0.1431
rs12424054	12	107186747	A	G	66.011	0.019	0.002	4.50E-16	-0.003	0.033	0.925
rs12440800	15	61960302	T	A	49.302	0.016	0.002	2.20E-12	0.010	0.031	0.7491
rs12446515	16	56987015	T	C	244.835	-0.033	0.002	3.50E-55	0.018	0.028	0.5251
rs12449427	17	7173279	C	G	35.877	-0.014	0.002	2.10E-09	0.004	0.029	0.9013

rs12470242	2	28546023	A	G	78.325	0.023	0.003	8.70E-19	0.033	0.028	0.2355
rs12472667	2	171629063	G	C	44.517	0.014	0.002	2.50E-11	0.035	0.027	0.192
rs12479355	2	227226952	G	A	61.864	-0.019	0.002	3.70E-15	-0.012	0.033	0.708101
rs12504746	4	39646631	T	C	36.662	-0.015	0.003	1.40E-09	-0.051	0.035	0.1485
rs12530679	7	106632113	G	A	36.173	-0.012	0.002	1.80E-09	-0.018	0.025	0.4702
rs12574790	11	116575687	T	C	318.819	0.184	0.010	2.60E-71	0.142	0.169	0.4014
rs12591786	15	60902512	T	C	40.580	-0.018	0.003	1.90E-10	0.009	0.042	0.8253
rs12669911	7	32262377	C	A	33.303	-0.012	0.002	7.90E-09	-0.023	0.028	0.4122
rs12791103	11	116673315	T	G	465.377	-0.072	0.003	3.30E-103	-0.065	0.039	0.0999793
rs12824533	12	11791685	G	A	39.724	0.013	0.002	2.90E-10	-0.007	0.026	0.7758
rs12880341	14	64236191	C	T	58.722	0.021	0.003	1.80E-14	-0.025	0.037	0.5113
rs12902047	15	41915522	C	A	36.299	-0.013	0.002	1.70E-09	-0.005	0.027	0.8387
rs12926107	16	88004092	G	A	39.864	0.013	0.002	2.70E-10	0.025	0.027	0.3621
rs12928099	16	15150505	A	C	167.268	-0.028	0.002	2.90E-38	-0.063	0.028	0.02512
rs12944954	17	7485131	G	A	37.185	0.040	0.007	1.10E-09	-0.003	0.084	0.9694
rs13064956	3	12170251	C	T	40.096	-0.020	0.003	2.40E-10	-0.029	0.038	0.4452
rs13066793	3	87037543	G	A	41.821	-0.022	0.003	1.00E-10	0.058	0.047	0.2129
rs13101828	4	965720	G	A	35.000	-0.012	0.002	3.30E-09	-0.019	0.025	0.4637
rs13107325	4	103188709	T	C	63.389	0.030	0.004	1.70E-15	0.046	0.108	0.668399
rs13108218	4	3443931	G	A	219.859	-0.031	0.002	9.70E-50	0.037	0.027	0.1752
rs13251367	8	72001931	C	T	30.227	-0.011	0.002	3.80E-08	0.024	0.026	0.3666
rs13269725	8	72459889	G	A	90.374	0.035	0.004	2.00E-21	0.019	0.067	0.782
rs13270882	8	19960861	T	C	39.109	0.030	0.005	4.00E-10	0.032	0.062	0.6031
rs13389219	2	165528876	T	C	344.516	-0.038	0.002	6.60E-77	-0.029	0.027	0.2808
rs1340819	13	29145323	C	A	33.729	-0.012	0.002	6.30E-09	0.023	0.027	0.4005
rs1347188	4	124743259	G	A	36.099	0.014	0.002	1.90E-09	0.064	0.029	0.0257199
rs1351394	12	66351826	C	T	45.647	0.013	0.002	1.40E-11	-0.029	0.025	0.2604
rs138191773	6	72522321	A	G	35.485	-0.047	0.008	2.60E-09	-0.040	0.208	0.8458
rs138751626	15	57154952	T	A	57.569	0.028	0.004	3.30E-14	0.083	0.064	0.1982

rs139076463	1	63110217	C	T	35.326	0.040	0.007	2.80E-09	-0.028	0.077	0.7175
rs139098834	2	28180473	A	G	43.808	-0.044	0.007	3.60E-11	-0.139	0.076	0.0665595
rs139453187	7	72155834	C	T	35.704	0.023	0.004	2.30E-09	-0.046	0.049	0.3481
rs139974673	15	44027885	C	T	525.934	0.143	0.006	2.20E-116	-0.229	0.177	0.1968
rs140107293	10	5267191	G	A	67.993	-0.023	0.003	1.60E-16	-0.019	0.037	0.611299
rs141415727	2	28221157	A	G	32.861	0.046	0.008	9.90E-09	0.013	0.156	0.9339
rs141469619	11	116714293	G	A	980.133	0.332	0.011	1.00E-200	-0.343	0.203	0.0909997
rs142848030	11	116871959	T	C	41.589	-0.047	0.007	1.10E-10	-0.107	0.088	0.2266
rs143076454	16	921179	A	G	30.183	0.040	0.007	3.90E-08	0.257	0.108	0.01736
rs144171645	1	63310797	A	T	41.246	-0.051	0.008	1.30E-10	0.013	0.121	0.9148
rs144246507	2	28297334	T	A	30.157	-0.042	0.008	4.00E-08	0.078	0.148	0.5967
rs144423449	2	27289677	A	G	42.841	-0.054	0.008	5.90E-11	-0.277	0.197	0.1595
rs144469617	8	19903238	A	G	43.461	0.052	0.008	4.30E-11	-0.110	0.159	0.490399
rs144470864	2	21183334	C	A	51.010	0.031	0.004	9.20E-13	0.042	0.050	0.4082
rs145692474	2	27712638	A	G	38.816	-0.079	0.013	4.70E-10	0.025	0.200	0.8995
rs14577578	12	65902265	T	C	34.528	-0.050	0.009	4.20E-09	-0.110	0.068	0.1046

5										
rs146203232	6	160543148	T	C	115.375	0.041	0.004	6.50E-27	-0.031	0.054	0.5606
rs148048657	8	19774005	A	G	32.097	0.038	0.007	1.50E-08	-0.044	0.085	0.6102
rs148482297	2	28415598	T	C	90.163	0.060	0.006	2.20E-21	0.054	0.125	0.6648
rs149214447	2	27102554	G	C	57.849	0.061	0.008	2.80E-14	0.070	0.252	0.780001
rs1495741	8	18272881	A	G	249.062	-0.038	0.002	4.20E-56	-0.039	0.029	0.1824
rs149719228	8	126567164	A	G	39.218	0.057	0.009	3.80E-10	0.159	0.154	0.3
rs149778057	13	31007977	C	A	50.110	-0.016	0.002	1.50E-12	-0.030	0.025	0.2385
rs1508103	11	116379978	G	A	101.690	0.042	0.004	6.50E-24	-0.033	0.065	0.604901
rs1534696	7	26397239	A	C	98.535	-0.020	0.002	3.20E-23	-0.010	0.026	0.686099
rs1552781	8	19624132	G	C	191.749	-0.033	0.002	1.30E-43	-0.016	0.028	0.5514
rs1556124	13	110991189	A	G	32.646	0.014	0.002	1.10E-08	0.012	0.034	0.7345
rs1561928	8	129568061	G	A	35.420	0.018	0.003	2.70E-09	-0.014	0.038	0.7185
rs1567353	9	1033773	G	C	46.920	0.015	0.002	7.40E-12	0.017	0.027	0.5252
rs157935	7	130585553	G	T	43.309	-0.014	0.002	4.70E-11	-0.026	0.026	0.3228
rs17146641	4	69555968	A	G	31.623	0.016	0.003	1.90E-08	-0.030	0.040	0.450001
rs1728407	16	86422112	G	A	33.987	0.012	0.002	5.50E-09	0.017	0.026	0.500299
rs17326656	2	48962291	T	G	55.846	0.017	0.002	7.80E-14	0.032	0.034	0.3431
rs174566	11	61592362	G	A	543.834	0.049	0.002	2.80E-120	-0.009	0.026	0.7405
rs17585887	6	139835498	C	T	200.933	-0.029	0.002	1.30E-45	0.010	0.025	0.6977
rs1760801	1	154259650	A	G	86.345	-0.020	0.002	1.50E-20	0.005	0.026	0.8354
rs1799831	7	44199142	T	C	80.019	0.025	0.003	3.70E-19	0.000	0.032	0.9923
rs1801689	17	64210580	C	A	131.027	-0.066	0.006	2.40E-30	-0.041	0.131	0.757
rs1823227	3	172240215	G	T	34.062	-0.013	0.002	5.30E-09	0.009	0.031	0.7703
rs1835346	6	161162290	G	A	35.832	-0.039	0.007	2.20E-09	0.062	0.104	0.550201

rs1840339	10	56634942	T	G	29.959	-0.012	0.002	4.40E-08	0.012	0.027	0.6693
rs184694823	1	150642998	G	A	31.946	0.021	0.004	1.60E-08	0.018	0.056	0.7524
rs185139895	6	34185863	A	G	79.765	0.043	0.005	4.20E-19	0.224	0.078	0.00386002
rs186696265	6	161111700	T	C	158.530	-0.104	0.008	2.40E-36	0.001	0.099	0.9939
rs1872992	12	26457190	G	A	40.154	0.015	0.002	2.30E-10	0.018	0.028	0.525
rs187544997	8	19890641	G	C	44.288	0.053	0.008	2.80E-11	0.037	0.261	0.8881
rs187929675	11	117111368	T	C	382.682	-0.171	0.009	3.20E-85	-0.240	0.120	0.0462498
rs193735	5	130672958	A	G	38.734	0.033	0.005	4.90E-10	0.007	0.050	0.8959
rs1938566	1	98478981	T	C	63.095	-0.021	0.003	2.00E-15	0.005	0.030	0.8759
rs2017500	15	99196112	A	G	33.960	0.012	0.002	5.60E-09	-0.018	0.025	0.492
rs2035816	4	100508556	G	A	60.941	-0.028	0.004	5.90E-15	0.058	0.074	0.4286
rs2043085	15	58680954	C	T	227.642	-0.031	0.002	1.90E-51	0.038	0.026	0.1385
rs2068888	10	94839642	A	G	254.197	-0.032	0.002	3.20E-57	-0.002	0.025	0.9318
rs2081687	8	59388565	C	T	156.216	-0.026	0.002	7.60E-36	-0.026	0.026	0.3161
rs2131311	1	40435999	G	A	31.079	-0.012	0.002	2.50E-08	0.023	0.028	0.4082
rs2131919	9	95283887	G	A	41.793	0.017	0.003	1.00E-10	0.007	0.033	0.8196
rs213209	6	33176958	T	C	30.921	0.012	0.002	2.70E-08	-0.022	0.028	0.4403
rs213494	1	54877103	T	C	56.556	0.016	0.002	5.50E-14	0.016	0.028	0.559899
rs2137557	3	188439236	C	T	31.978	0.012	0.002	1.60E-08	0.051	0.027	0.0557699
rs2165557	8	19835050	T	A	336.025	-0.047	0.003	4.70E-75	-0.041	0.034	0.227
rs2237029	4	55535336	A	G	46.685	-0.014	0.002	8.30E-12	0.032	0.026	0.222
rs2240466	7	72856269	A	G	1654.249	-0.123	0.003	1.00E-200	-0.091	0.038	0.01661
rs2240533	14	71541026	C	T	36.423	-0.013	0.002	1.60E-09	0.043	0.028	0.1226
rs2244278	9	107661129	A	C	77.736	-0.027	0.003	1.20E-18	0.013	0.044	0.763199
rs2276853	3	47282303	A	G	30.243	0.011	0.002	3.80E-08	0.043	0.026	0.0963097
rs2277083	6	133827354	G	A	58.723	-0.015	0.002	1.80E-14	-0.017	0.025	0.490399

rs2288004	16	31054040	C	G	54.031	-0.015	0.002	2.00E-13	0.020	0.026	0.4411
rs2302104	5	140047600	T	C	36.148	-0.014	0.002	1.80E-09	0.032	0.029	0.264
rs2302364	11	62190700	C	T	34.641	0.016	0.003	4.00E-09	0.041	0.037	0.2704
rs2382825	2	219184275	T	C	43.256	-0.013	0.002	4.80E-11	-0.055	0.029	0.0556596
rs2407278	8	37399290	G	A	34.022	-0.034	0.006	5.40E-09	0.006	0.057	0.9106
rs241771	17	26592946	C	T	32.136	0.011	0.002	1.40E-08	-0.015	0.025	0.5449
rs2420477	10	120242187	C	T	31.339	-0.011	0.002	2.20E-08	0.020	0.025	0.4297
rs2439364	15	66876675	G	A	45.176	-0.013	0.002	1.80E-11	0.002	0.025	0.9279
rs245051	5	149345975	G	A	36.018	-0.012	0.002	2.00E-09	-0.051	0.026	0.0534404
rs2455821	3	15681940	A	C	36.283	0.013	0.002	1.70E-09	0.020	0.029	0.4977
rs2487294	10	113937941	T	G	68.041	0.018	0.002	1.60E-16	0.032	0.027	0.2393
rs2517887	6	29807393	C	G	36.368	0.015	0.002	1.60E-09	-0.058	0.031	0.0623993
rs2519093	9	136141870	T	C	68.207	-0.021	0.003	1.50E-16	-0.045	0.031	0.1554
rs2528787	13	112188236	T	C	30.241	-0.011	0.002	3.80E-08	0.008	0.027	0.754899
rs268	8	19813529	G	A	941.512	0.227	0.007	1.00E-200	-0.049	0.085	0.566601
rs275184	15	39444679	G	T	39.723	-0.017	0.003	2.90E-10	0.017	0.032	0.582599
rs2761294	10	95067043	A	G	82.739	0.019	0.002	9.40E-20	-0.003	0.026	0.8974
rs2773469	10	115798895	G	A	70.083	-0.019	0.002	5.70E-17	0.004	0.029	0.8845
rs278981	4	40428010	C	T	30.197	0.013	0.002	3.90E-08	-0.029	0.030	0.3373
rs2800713	6	127383980	A	G	50.010	0.031	0.004	1.50E-12	-0.039	0.046	0.3988
rs2812208	13	50707087	C	G	48.366	-0.048	0.007	3.50E-12	-0.125	0.087	0.1496
rs28383314	6	32587213	C	T	342.908	0.038	0.002	1.50E-76	-0.031	0.030	0.2936
rs28481932	6	31242900	G	A	54.018	0.022	0.003	2.00E-13	0.050	0.039	0.2041
rs28546518	8	19934043	G	A	69.423	-0.025	0.003	7.90E-17	0.039	0.032	0.2281
rs28577186	16	4488191	A	G	59.611	-0.016	0.002	1.20E-14	-0.034	0.026	0.1941
rs28624578	15	31637666	C	T	32.022	0.015	0.003	1.50E-08	0.028	0.033	0.4035
rs28732146	6	31561353	A	T	110.229	0.026	0.002	8.70E-26	0.030	0.033	0.3695
rs28873097	4	88408172	C	T	31.137	0.011	0.002	2.40E-08	-0.026	0.025	0.3027
rs2917677	16	69750849	T	C	87.876	-0.019	0.002	7.00E-21	-0.066	0.026	0.00911906
rs2925979	16	81534790	C	T	220.120	-0.032	0.002	8.50E-50	0.006	0.027	0.8261
rs2943645	2	227099180	T	C	378.320	0.040	0.002	2.90E-84	0.052	0.026	0.04917

rs2954011	8	126424379	T	C	54.882	-0.015	0.002	1.30E-13	-0.002	0.026	0.9532
rs2983896	6	97029871	A	G	32.429	0.014	0.002	1.20E-08	-0.014	0.032	0.658901
rs308	8	19817476	G	T	520.939	-0.159	0.007	2.60E-115	0.082	0.074	0.271
rs3097858	5	155571886	T	A	34.523	0.020	0.003	4.20E-09	-0.009	0.041	0.8276
rs3103310	3	12473045	G	A	73.255	0.020	0.002	1.10E-17	-0.019	0.034	0.5673
rs320369	1	118143517	G	A	34.116	-0.013	0.002	5.20E-09	-0.031	0.026	0.2309
rs325485	5	103995368	G	A	33.125	-0.012	0.002	8.60E-09	-0.032	0.027	0.2331
rs326222	11	47259668	C	T	136.713	0.025	0.002	1.40E-31	-0.009	0.027	0.7274
rs3289	8	19823192	C	T	645.385	0.154	0.006	2.30E-142	-0.092	0.092	0.3148
rs34144542	11	116774447	G	A	104.507	-0.039	0.004	1.60E-24	0.026	0.053	0.6265
rs34196215	5	55965964	C	T	39.385	-0.018	0.003	3.50E-10	-0.022	0.039	0.5799
rs34234296	2	175166636	A	G	30.954	-0.011	0.002	2.60E-08	-0.011	0.028	0.702301
rs343	8	19810787	A	C	1535.785	-0.141	0.004	1.00E-200	-0.012	0.052	0.8243
rs34580448	5	82810884	C	T	46.234	-0.034	0.005	1.00E-11	0.111	0.094	0.2381
rs34893217	8	16279180	T	G	31.820	-0.018	0.003	1.70E-08	-0.057	0.042	0.17
rs35104374	12	6739497	C	T	48.157	-0.016	0.002	3.90E-12	0.024	0.030	0.4246
rs35169799	11	64031241	T	C	187.866	0.056	0.004	9.30E-43	0.059	0.043	0.1752
rs35200015	11	117383215	A	G	47.029	0.017	0.003	7.00E-12	-0.051	0.028	0.0704904
rs35477346	15	40400195	C	T	38.944	0.014	0.002	4.40E-10	0.000	0.027	0.9967
rs35763453	12	46215895	C	T	42.748	0.028	0.004	6.20E-11	0.070	0.067	0.2979
rs35859536	8	118191475	T	C	44.891	-0.014	0.002	2.10E-11	0.005	0.026	0.8602
rs36043408	1	203511492	A	G	41.606	-0.013	0.002	1.10E-10	0.013	0.026	0.621901
rs36061954	8	38329650	T	C	33.709	0.012	0.002	6.40E-09	-0.010	0.026	0.695
rs3731696	2	203431804	G	A	52.150	0.022	0.003	5.10E-13	0.126	0.038	0.00107701
rs3738182	1	221057662	A	G	36.565	0.015	0.003	1.50E-09	0.008	0.030	0.7931
rs3758413	10	17268839	C	T	30.392	0.011	0.002	3.50E-08	-0.010	0.026	0.7082
rs3775228	4	87985166	T	C	275.915	0.034	0.002	5.80E-62	-0.034	0.026	0.1844
rs37774222<br>8	1	149844763	G	A	46.919	-0.025	0.004	7.40E-12	0.059	0.055	0.2827
rs3784310	15	72103427	C	T	34.321	-0.013	0.002	4.70E-09	-0.034	0.027	0.2142
rs3794695	16	72097827	T	C	120.748	0.028	0.003	4.30E-28	-0.014	0.032	0.657201

rs3808477	8	116670347	T	C	36.904	-0.013	0.002	1.20E-09	-0.044	0.027	0.1072
rs3814883	16	29994922	T	C	55.638	0.015	0.002	8.70E-14	0.062	0.026	0.0149101
rs38189	7	15903403	T	A	55.126	-0.015	0.002	1.10E-13	-0.013	0.027	0.617999
rs3820897	2	3642361	C	T	57.432	0.020	0.003	3.50E-14	0.059	0.027	0.0284702
rs3826043	15	73618238	T	C	36.335	-0.012	0.002	1.70E-09	-0.077	0.026	0.00249201
rs3829126	10	74714177	T	G	45.295	0.023	0.003	1.70E-11	-0.042	0.052	0.4211
rs3936511	5	55860781	G	A	334.623	0.046	0.003	9.50E-75	0.057	0.037	0.125
rs3974807	11	49161967	T	C	39.843	0.016	0.003	2.80E-10	0.050	0.037	0.1787
rs40270	5	55804552	C	A	227.216	0.036	0.002	2.40E-51	-0.005	0.028	0.852
rs4128205	2	165467068	C	A	33.244	0.012	0.002	8.10E-09	0.005	0.026	0.833
rs4134963	6	20486798	T	C	55.584	-0.019	0.003	9.00E-14	-0.019	0.032	0.5566
rs41785	7	116486020	A	C	55.749	-0.015	0.002	8.20E-14	-0.052	0.026	0.0441205
rs4382584	9	139386138	A	G	33.525	0.013	0.002	7.00E-09	-0.028	0.029	0.3347
rs4387830	2	21423281	A	G	68.531	0.027	0.003	1.20E-16	0.039	0.047	0.4014
rs4391855	11	116350751	A	G	133.262	0.059	0.005	7.90E-31	0.000	0.077	0.996
rs4445477	1	219759481	A	G	46.287	-0.016	0.002	1.00E-11	-0.021	0.031	0.4928
rs4471666	16	85198965	G	T	32.058	-0.022	0.004	1.50E-08	-0.028	0.045	0.539299
rs4559942	17	73309269	A	G	32.654	0.014	0.002	1.10E-08	0.069	0.033	0.0364502
rs4564007	9	134854280	C	T	33.336	-0.012	0.002	7.80E-09	0.004	0.028	0.8725
rs4662414	2	145818432	G	A	36.645	-0.012	0.002	1.40E-09	-0.058	0.026	0.0234801
rs4665972	2	27598097	C	T	2424.998	-0.100	0.002	1.00E-200	-0.128	0.026	9.03E-07
rs4675812	2	242395674	A	G	50.037	-0.014	0.002	1.50E-12	-0.023	0.026	0.379
rs4699983	5	55992718	G	A	43.754	-0.013	0.002	3.70E-11	-0.010	0.025	0.6822
rs4709746	6	164133001	T	C	42.508	-0.019	0.003	7.00E-11	-0.003	0.047	0.9497
rs4714001	6	36638175	A	G	35.810	0.012	0.002	2.20E-09	-0.005	0.027	0.8578
rs4731701	7	130430930	T	C	268.256	-0.033	0.002	2.70E-60	-0.006	0.025	0.807
rs4760254	12	57766392	C	G	146.518	-0.028	0.002	1.00E-33	-0.097	0.030	0.00115199
rs4761234	12	69732105	C	T	49.474	-0.014	0.002	2.00E-12	-0.024	0.026	0.3421
rs478420	8	17792853	A	G	38.886	0.013	0.002	4.50E-10	-0.007	0.026	0.793299
rs4793090	17	40686342	A	G	47.342	-0.014	0.002	6.00E-12	0.009	0.026	0.7229
rs480823	11	116525730	C	T	1752.523	0.156	0.004	1.00E-200	0.004	0.035	0.9002

rs4846857	1	230436175	G	A	63.507	-0.023	0.003	1.60E-15	-0.040	0.029	0.1754
rs4909945	11	10673739	C	T	39.199	0.013	0.002	3.80E-10	0.003	0.027	0.9093
rs4938277	11	116397532	T	C	67.313	0.018	0.002	2.30E-16	0.011	0.027	0.6822
rs4969179	17	76391454	G	T	75.993	-0.018	0.002	2.80E-18	0.083	0.028	0.00264198
rs4976033	5	67714246	G	A	75.186	0.018	0.002	4.30E-18	0.005	0.026	0.8528
rs498475	7	28256240	A	G	32.100	-0.012	0.002	1.50E-08	-0.036	0.027	0.1781
rs499293	11	30504660	A	G	31.859	-0.012	0.002	1.70E-08	-0.080	0.026	0.00217901
rs534417	1	23784965	G	A	42.692	0.020	0.003	6.40E-11	-0.033	0.040	0.4011
rs535241194	7	150299483	G	A	73.368	0.021	0.002	1.10E-17	0.027	0.027	0.3183
rs536338	13	51103461	G	C	39.888	0.014	0.002	2.70E-10	-0.046	0.030	0.1248
rs540658636	7	72792387	C	T	38.976	0.033	0.005	4.30E-10	0.088	0.048	0.0658506
rs55646464	5	173324971	T	G	31.559	0.012	0.002	1.90E-08	-0.045	0.026	0.0759994
rs55692143	8	19990450	C	T	30.319	0.017	0.003	3.70E-08	0.020	0.038	0.5949
rs55697600	6	32604166	G	A	174.425	0.068	0.005	8.00E-40	-0.108	0.068	0.1124
rs55802560	10	94558127	C	T	40.129	-0.021	0.003	2.40E-10	-0.041	0.041	0.3235
rs55931203	17	65857714	T	C	85.002	0.024	0.003	3.00E-20	0.008	0.030	0.7798
rs55951611	7	73126343	A	G	64.525	-0.039	0.005	9.50E-16	0.014	0.071	0.8398
rs56057635	11	117349401	G	A	32.952	0.017	0.003	9.40E-09	-0.026	0.043	0.5509
rs56311339	11	116566161	T	C	137.234	-0.037	0.003	1.10E-31	0.052	0.041	0.2034
rs563296	10	99772404	A	G	67.708	0.016	0.002	1.90E-16	0.055	0.025	0.0300497
rs56902258	14	23733114	A	T	36.316	-0.015	0.003	1.70E-09	0.045	0.032	0.1599
rs56959712	12	123188475	T	G	79.740	-0.022	0.002	4.30E-19	-0.025	0.032	0.436
rs5743840	3	52260212	A	T	34.540	0.037	0.006	4.20E-09	0.104	0.157	0.5094
rs575623	11	116278812	T	C	46.165	0.022	0.003	1.10E-11	0.066	0.048	0.176
rs58038553	15	58574324	G	A	47.096	-0.020	0.003	6.80E-12	0.101	0.042	0.0174699
rs581080	9	15305378	C	G	46.078	0.018	0.003	1.10E-11	-0.025	0.036	0.4895
rs5880	16	57015091	C	G	86.511	0.040	0.004	1.40E-20	0.019	0.081	0.8139
rs58839393	2	43490619	T	A	41.662	0.017	0.003	1.10E-10	-0.012	0.034	0.725
rs595767	17	46957987	G	A	46.686	0.014	0.002	8.30E-12	-0.035	0.025	0.1627

rs612874	2	219492924	C	T	80.286	0.018	0.002	3.20E-19	-0.029	0.026	0.2568
rs61780049	1	39363294	G	A	30.097	0.015	0.003	4.10E-08	-0.023	0.039	0.5576
rs61785481	1	110497808	A	G	30.233	0.011	0.002	3.80E-08	-0.018	0.026	0.480799
rs61821643	1	219268532	C	T	32.322	0.037	0.007	1.30E-08	-0.132	0.142	0.3538
rs61830291	1	221001142	C	A	72.380	0.029	0.003	1.80E-17	-0.013	0.040	0.750699
rs61885960	11	14819828	A	T	50.224	-0.031	0.004	1.40E-12	0.017	0.045	0.7094
rs61904855	11	116476668	A	C	83.507	0.054	0.006	6.40E-20	-0.003	0.101	0.9748
rs61907563	11	116733565	A	G	149.805	-0.068	0.006	1.90E-34	0.066	0.052	0.2044
rs61975915	14	58665283	T	C	29.964	-0.012	0.002	4.40E-08	-0.015	0.029	0.6167
rs61993685	14	100765823	C	T	39.176	-0.023	0.004	3.90E-10	-0.054	0.047	0.2538
rs62064941	16	58834402	C	A	30.849	-0.029	0.005	2.80E-08	-0.131	0.084	0.1196
rs62271373	3	150066540	A	T	96.769	0.042	0.004	7.80E-23	-0.011	0.062	0.8569
rs62274099	3	156810578	T	C	35.661	0.012	0.002	2.30E-09	0.003	0.026	0.9236
rs62397245	5	176750688	G	C	39.151	0.015	0.002	3.90E-10	0.027	0.029	0.3574
rs62427982	6	107437166	T	C	39.007	-0.013	0.002	4.20E-10	-0.012	0.026	0.6436
rs62463387	7	72349865	A	G	40.933	0.034	0.005	1.60E-10	-0.018	0.055	0.742699
rs62492368	7	150537635	A	G	42.662	0.014	0.002	6.50E-11	0.038	0.027	0.1509
rs62565259	9	102162570	T	C	41.281	-0.017	0.003	1.30E-10	0.029	0.037	0.437
rs6458869	6	52630269	A	C	73.714	-0.018	0.002	9.00E-18	0.002	0.026	0.929
rs6465120	7	76036364	G	A	40.670	-0.013	0.002	1.80E-10	0.025	0.026	0.3265
rs647071	11	111661455	G	C	55.721	-0.015	0.002	8.40E-14	-0.029	0.026	0.262
rs6494391	15	63406785	C	T	60.990	-0.019	0.002	5.70E-15	0.038	0.028	0.1793
rs6532798	4	100054827	T	C	40.638	0.014	0.002	1.80E-10	-0.015	0.028	0.5921
rs6562773	13	74188935	G	A	35.943	-0.012	0.002	2.00E-09	-0.030	0.025	0.2421
rs6572807	14	52480621	G	A	30.773	0.012	0.002	2.90E-08	0.017	0.028	0.5568
rs66884613	2	28046719	G	A	42.187	-0.045	0.007	8.30E-11	-0.194	0.176	0.2705
rs6690181	1	228056868	C	T	46.111	-0.014	0.002	1.10E-11	0.011	0.026	0.6576
rs6700266	1	178508930	A	G	35.378	-0.012	0.002	2.70E-09	-0.056	0.026	0.0289501
rs6708682	2	64976815	G	A	36.922	0.013	0.002	1.20E-09	0.010	0.028	0.727601
rs6708784	2	111927379	G	A	42.311	-0.013	0.002	7.80E-11	-0.049	0.025	0.0517297
rs676210	2	21231524	A	G	900.649	-0.074	0.002	7.10E-198	0.019	0.029	0.519

rs6792725	3	24520283	G	A	47.263	-0.015	0.002	6.20E-12	-0.008	0.026	0.7605
rs67981690	12	21343886	G	A	101.194	0.030	0.003	8.30E-24	-0.072	0.032	0.0259699
rs6800707	3	52516293	G	C	138.614	0.030	0.003	5.30E-32	0.061	0.043	0.1573
rs6805924	3	69879670	T	G	29.905	0.011	0.002	4.50E-08	-0.003	0.026	0.905
rs684773	3	135956305	C	A	153.847	0.029	0.002	2.50E-35	0.020	0.035	0.5752
rs6865870	5	144498018	G	A	31.762	0.012	0.002	1.70E-08	0.026	0.028	0.3424
rs6882076	5	156390297	C	T	257.891	0.033	0.002	4.90E-58	-0.054	0.027	0.04561
rs6913325	6	106378009	T	G	30.337	-0.011	0.002	3.60E-08	-0.031	0.026	0.2457
rs6924805	6	18747705	T	G	30.502	-0.011	0.002	3.30E-08	-0.006	0.026	0.8342
rs696825	9	86583076	T	C	78.619	-0.020	0.002	7.50E-19	-0.005	0.027	0.8571
rs6968865	7	17287269	T	A	54.190	0.015	0.002	1.80E-13	-0.048	0.027	0.0717596
rs6977665	7	116976830	G	A	45.256	0.014	0.002	1.70E-11	-0.017	0.025	0.506001
rs698927	1	93836218	C	A	51.013	-0.018	0.003	9.20E-13	-0.018	0.033	0.592
rs6995541	8	10671260	G	A	158.303	0.028	0.002	2.70E-36	0.032	0.029	0.2658
rs6999569	8	126475770	G	A	1889.263	-0.086	0.002	1.00E-200	-0.005	0.025	0.8316
rs7077812	10	81096071	C	T	32.234	0.014	0.003	1.40E-08	0.036	0.032	0.2487
rs7090871	10	63830286	C	T	41.781	-0.013	0.002	1.00E-10	-0.017	0.026	0.516799
rs7096101	10	94362928	A	G	42.837	-0.013	0.002	5.90E-11	0.011	0.026	0.6665
rs7104819	11	118769442	G	A	31.491	0.011	0.002	2.00E-08	0.024	0.026	0.3559
rs7124396	11	47814280	G	T	31.234	0.011	0.002	2.30E-08	-0.045	0.026	0.0861708
rs7140110	13	114544024	C	T	169.021	0.028	0.002	1.20E-38	0.037	0.028	0.1856
rs71473777	10	77217080	G	A	37.004	0.019	0.003	1.20E-09	-0.026	0.039	0.5136
rs71538127	7	1010801	G	C	33.769	0.018	0.003	6.20E-09	-0.018	0.043	0.6742
rs71603401	4	18034463	G	A	82.496	0.026	0.003	1.10E-19	0.034	0.047	0.4767
rs7210745	17	74230635	C	T	36.226	0.022	0.004	1.80E-09	-0.084	0.061	0.1681
rs7215055	17	17458353	G	A	90.099	0.039	0.004	2.30E-21	0.036	0.047	0.436
rs7244	5	153800513	A	G	33.697	0.015	0.003	6.40E-09	-0.018	0.033	0.589599
rs72669514	1	63194030	T	C	65.274	0.038	0.005	6.50E-16	-0.037	0.061	0.546
rs72691637	8	144306970	A	G	36.146	-0.015	0.003	1.80E-09	0.020	0.031	0.5218
rs72801474	5	132444128	A	G	80.061	-0.031	0.003	3.60E-19	0.077	0.055	0.1614
rs72810512	2	28192597	T	C	59.779	-0.028	0.004	1.10E-14	0.049	0.047	0.3024

rs72904737	1	51351846	A	G	59.539	-0.027	0.004	1.20E-14	-0.008	0.055	0.888
rs729761	6	43804571	G	T	64.521	0.018	0.002	9.50E-16	0.062	0.028	0.0261999
rs73190070	8	8700258	A	G	34.375	0.015	0.003	4.50E-09	0.034	0.032	0.2914
rs73210895	8	20021205	G	T	86.151	-0.019	0.002	1.70E-20	-0.006	0.027	0.8133
rs73243877	4	26047616	G	A	117.713	0.029	0.003	2.00E-27	-0.015	0.036	0.6762
rs742036	16	960725	A	G	49.037	-0.014	0.002	2.50E-12	0.052	0.027	0.0527096
rs7424120	2	59313974	T	C	37.025	-0.012	0.002	1.20E-09	-0.030	0.026	0.2418
rs74662600	11	116654959	A	G	103.449	0.083	0.008	2.70E-24	-0.080	0.063	0.1996
rs75033071	8	19652910	G	A	66.372	0.064	0.008	3.70E-16	-0.017	0.166	0.9207
rs75164422	8	126507098	A	G	30.132	-0.032	0.006	4.00E-08	-0.052	0.079	0.5121
rs75268115	11	18301915	G	A	33.577	-0.021	0.004	6.80E-09	0.082	0.043	0.0564196
rs75398587	10	103946480	G	C	41.022	-0.025	0.004	1.50E-10	-0.076	0.066	0.2516
rs75609851	8	19871080	A	G	393.311	-0.199	0.010	1.60E-87	0.004	0.112	0.972
rs75612388	2	28540127	G	A	32.483	-0.034	0.006	1.20E-08	-0.052	0.057	0.3621
rs75679663	17	4667972	A	C	47.826	-0.075	0.011	4.70E-12	0.071	0.077	0.3559
rs7588724	2	25923122	G	A	31.283	-0.012	0.002	2.20E-08	-0.046	0.026	0.0761605
rs75919952	11	116667545	T	C	452.127	-0.097	0.005	2.50E-100	0.098	0.064	0.1273
rs7596814	2	230128204	T	G	34.234	-0.013	0.002	4.90E-09	0.031	0.032	0.3371
rs75999908	1	63406978	A	G	49.653	0.024	0.003	1.80E-12	0.059	0.047	0.2068
rs7600413	2	37067137	G	A	50.028	-0.014	0.002	1.50E-12	-0.049	0.025	0.0515798
rs76172517	2	169514699	C	T	31.837	-0.018	0.003	1.70E-08	-0.044	0.046	0.3427
rs76814754	11	116557959	T	C	42.614	-0.046	0.007	6.70E-11	0.069	0.113	0.5404
rs76862947	16	79746461	C	T	34.072	0.013	0.002	5.30E-09	-0.021	0.028	0.4603
rs76884843	12	57349761	T	C	30.297	-0.048	0.009	3.70E-08	-0.087	0.107	0.4163
rs77009508	6	161004972	G	A	140.807	0.045	0.004	1.80E-32	0.035	0.052	0.5045
rs7704653	5	90255685	G	A	49.203	0.016	0.002	2.30E-12	-0.039	0.029	0.1814
rs77053629	11	61519815	T	C	37.486	-0.028	0.005	9.20E-10	-0.148	0.068	0.0306803
rs7714361	5	112490629	C	A	34.437	0.014	0.002	4.40E-09	-0.011	0.028	0.6951
rs77244849	17	74281391	C	T	44.798	-0.014	0.002	2.20E-11	-0.040	0.029	0.1714
rs7735249	5	53310139	G	C	72.270	0.027	0.003	1.90E-17	0.064	0.039	0.0988394
rs77631110	2	119752450	C	A	42.086	0.052	0.008	8.70E-11	0.010	0.191	0.9566

rs77675968	8	19959983	C	T	92.789	0.054	0.006	5.80E-22	0.073	0.060	0.2182
rs77824033	9	112241136	C	T	32.104	-0.030	0.005	1.50E-08	-0.114	0.074	0.1235
rs7786102	7	25965759	A	G	166.154	-0.028	0.002	5.10E-38	-0.007	0.027	0.7863
rs78025076	4	110569620	T	C	48.136	0.049	0.007	4.00E-12	-0.219	0.138	0.1112
rs78034258	8	19239161	C	T	34.319	-0.023	0.004	4.70E-09	0.008	0.056	0.8796
rs78058190	2	219699999	A	G	255.394	0.082	0.005	1.70E-57	0.082	0.047	0.0766408
rs7826687	8	126645689	G	C	178.645	0.029	0.002	9.60E-41	0.020	0.028	0.4772
rs78297458	11	50611510	C	T	29.787	-0.037	0.007	4.80E-08	-0.198	0.222	0.3707
rs7855395	9	13676484	G	A	33.298	-0.012	0.002	7.90E-09	0.028	0.026	0.2678
rs78588343	6	7249460	A	G	35.550	-0.016	0.003	2.50E-09	-0.067	0.031	0.02712
rs7861679	9	123375589	T	C	31.697	0.012	0.002	1.80E-08	0.003	0.028	0.9045
rs78751065	11	117171413	T	C	58.173	0.058	0.008	2.40E-14	-0.132	0.135	0.3305
rs78933533	6	32604198	A	G	95.276	-0.052	0.005	1.70E-22	0.043	0.063	0.4953
rs79287178	3	172294500	A	G	69.607	0.050	0.006	7.20E-17	0.051	0.063	0.4238
rs79311290	16	85150163	G	A	41.925	0.022	0.003	9.50E-11	0.024	0.056	0.668599
rs7947951	11	13356030	G	A	81.936	0.019	0.002	1.40E-19	0.033	0.025	0.1893
rs7951019	11	118358027	G	T	41.515	-0.037	0.006	1.20E-10	0.098	0.072	0.1698
rs79634051	11	14561945	C	G	50.975	-0.043	0.006	9.40E-13	-0.162	0.070	0.0202302
rs7966846	12	57653582	C	A	39.847	0.014	0.002	2.70E-10	0.003	0.026	0.9172
rs79778375	11	117545083	A	G	33.607	-0.029	0.005	6.70E-09	-0.006	0.053	0.9086
rs79859736	11	116269946	A	G	36.106	0.037	0.006	1.90E-09	0.005	0.063	0.9389
rs79873985	14	24560159	G	A	30.908	-0.045	0.008	2.70E-08	0.187	0.185	0.3113
rs799157	7	73020301	C	T	310.492	-0.086	0.005	1.70E-69	-0.162	0.085	0.0552599
rs79983121	3	127306462	T	C	37.119	0.015	0.002	1.10E-09	-0.039	0.029	0.1719
rs80216311	2	27301057	T	C	96.206	-0.034	0.003	1.00E-22	0.023	0.054	0.671001
rs80276949	10	52373245	A	G	46.778	0.046	0.007	8.00E-12	0.059	0.116	0.612999
rs8046718	16	2462739	T	C	38.600	-0.013	0.002	5.20E-10	-0.039	0.027	0.1423
rs8066985	17	68453345	G	A	40.246	-0.013	0.002	2.20E-10	0.005	0.026	0.8423
rs840974	2	65705581	A	G	35.090	-0.012	0.002	3.10E-09	-0.039	0.027	0.145
rs852388	7	5574239	C	G	40.928	0.016	0.002	1.60E-10	-0.049	0.039	0.2011
rs878409	10	122999550	A	G	31.825	-0.011	0.002	1.70E-08	-0.012	0.025	0.6401

rs878521	7	44255643	A	G	65.157	0.018	0.002	6.90E-16	-0.015	0.031	0.6206
rs879986	8	126569934	G	T	46.722	0.036	0.005	8.20E-12	-0.026	0.118	0.8234
rs880315	1	10796866	C	T	31.276	-0.012	0.002	2.20E-08	0.066	0.026	0.0108901
rs917195	7	30728452	T	C	30.293	-0.013	0.002	3.70E-08	-0.003	0.032	0.9325
rs933574	16	11792700	C	A	38.070	0.012	0.002	6.80E-10	-0.013	0.025	0.608501
rs935168	2	26914787	A	G	46.360	0.014	0.002	9.80E-12	-0.001	0.026	0.9741
rs9375694	6	130356608	A	G	67.355	0.018	0.002	2.30E-16	0.025	0.029	0.3893
rs9376511	6	140611419	G	A	39.532	-0.015	0.002	3.20E-10	0.013	0.031	0.687999
rs9405064	6	31302841	C	A	131.525	0.029	0.003	1.90E-30	0.009	0.052	0.8674
rs9425589	1	172355276	A	G	47.261	-0.014	0.002	6.20E-12	0.001	0.025	0.9776
rs9436661	1	62904575	G	T	1399.394	-0.078	0.002	1.00E-200	-0.017	0.029	0.558
rs947799	11	64851305	T	C	30.895	-0.012	0.002	2.70E-08	-0.004	0.026	0.882
rs9480889	6	109189021	G	C	45.779	0.016	0.002	1.30E-11	0.054	0.037	0.1444
rs9496567	6	100602753	A	G	35.343	-0.014	0.002	2.80E-09	0.030	0.031	0.3341
rs954244	2	121309231	G	C	45.396	0.015	0.002	1.60E-11	-0.017	0.032	0.597901
rs9553567	13	25820101	C	T	30.504	0.015	0.003	3.30E-08	0.082	0.036	0.02274
rs9561643	13	95253131	C	A	60.734	0.017	0.002	6.50E-15	0.001	0.026	0.9725
rs9584870	13	99245866	C	T	34.496	-0.012	0.002	4.30E-09	0.025	0.027	0.3568
rs966541	12	29491528	G	A	32.930	-0.013	0.002	9.60E-09	0.030	0.028	0.2846
rs970069	5	140888248	T	C	44.455	0.016	0.002	2.60E-11	0.005	0.032	0.8808
rs9788220	12	109699616	C	T	38.373	0.016	0.003	5.80E-10	0.028	0.036	0.4395
rs9831084	3	37025661	C	T	35.236	-0.012	0.002	2.90E-09	0.039	0.026	0.1364
rs9890200	17	48624523	C	A	40.155	-0.013	0.002	2.30E-10	-0.006	0.027	0.833
rs9894946	17	7571080	G	A	43.632	-0.018	0.003	4.00E-11	0.029	0.036	0.4078
rs9906319	17	64190994	T	C	36.268	-0.024	0.004	1.70E-09	0.015	0.042	0.7267
rs9923575	16	72230112	T	C	38.055	-0.015	0.003	6.90E-10	0.041	0.032	0.2069
rs9943778	12	22765864	G	A	42.649	-0.015	0.002	6.50E-11	0.006	0.029	0.8306
rs9944241	15	51900651	C	T	36.988	-0.013	0.002	1.20E-09	0.010	0.025	0.692201
rs9970140	1	61684288	G	A	46.395	-0.026	0.004	9.70E-12	0.051	0.047	0.2764
rs998584	6	43757896	A	C	406.426	0.040	0.002	2.20E-90	0.015	0.025	0.550301

rs9991328	4	89713121	T	C	56.691	0.015	0.002	5.10E-14	-0.032	0.025	0.2061

SNP	Chr	Position	Effect Allele	Other Allele	F	Association with exposure			Association with Gout		
						**Beta**	**SE**	***P***	**Beta**	**SE**	***P***
**TC**											
rs10104187	8	141978460	C	T	31.890	0.012	0.002	2.40E-08	0.002	0.025	0.9485
rs10104997	8	55440068	T	C	71.125	0.021	0.003	3.90E-18	0.010	0.030	0.7362
rs10114039	9	19352260	T	A	44.386	0.027	0.004	3.90E-12	-0.024	0.053	0.6556
rs10128711	11	18632984	C	T	57.671	0.018	0.002	5.90E-15	-0.008	0.026	0.7462
rs10175646	2	21145665	C	T	56.748	-0.042	0.006	1.00E-16	0.045	0.069	0.5141
rs10184376	2	158442005	T	C	30.665	-0.021	0.004	4.10E-09	0.011	0.054	0.8449
rs10189685	2	203488449	A	G	109.869	-0.023	0.002	3.30E-26	0.016	0.029	0.5682
rs103294	19	54797848	T	C	73.624	0.021	0.002	1.50E-18	0.046	0.028	0.0987211
rs1034621	17	67960949	C	T	27.542	0.011	0.002	3.20E-08	-0.030	0.027	0.2705
rs1035543	16	71983772	C	G	89.314	0.020	0.002	3.60E-23	0.051	0.026	0.0503698
rs10419669	19	45298069	A	G	30.073	0.022	0.004	1.30E-08	0.047	0.039	0.2322
rs1042023	2	21229446	C	G	68.784	0.082	0.010	1.50E-18	0.071	0.125	0.5722
rs10490625	2	118859556	A	G	45.983	0.027	0.004	3.80E-12	0.035	0.036	0.3334
rs1051338	10	91007360	G	T	26.271	-0.011	0.002	1.40E-08	0.000	0.026	0.9979
rs1057868	7	75615006	T	C	33.497	0.013	0.002	5.00E-09	-0.002	0.026	0.9307
rs10773112	12	125338529	T	C	48.189	0.016	0.002	4.10E-13	0.006	0.029	0.8263
rs1077514	1	23766233	T	C	61.337	0.023	0.003	1.30E-15	-0.019	0.039	0.6265
rs10794579	10	124686656	C	T	63.659	0.017	0.002	2.00E-16	-0.005	0.026	0.8415
rs10822184	10	65337153	C	T	33.994	0.012	0.002	2.00E-09	-0.050	0.025	0.0503802
rs10896125	11	66288733	C	G	38.921	-0.015	0.002	6.20E-10	0.074	0.026	0.00454496
rs10903129	1	25768937	G	A	172.931	0.027	0.002	8.00E-43	-0.012	0.025	0.6478
rs10919615	1	198974904	T	C	27.951	0.013	0.002	3.40E-08	-0.002	0.029	0.9398

rs11057273	12	123814466	C	T	68.051	-0.029	0.004	1.60E-16	-0.034	0.040	0.3962
rs11078597	17	1618363	C	T	36.495	0.016	0.003	1.10E-09	-0.017	0.032	0.6084
rs11088472	21	40699376	C	A	31.862	0.012	0.002	2.20E-09	0.025	0.027	0.3551
rs11136343	8	145058986	G	A	39.457	0.014	0.002	1.70E-10	-0.013	0.026	0.604
rs11195857	10	114022566	A	G	44.969	-0.018	0.003	1.70E-12	-0.020	0.034	0.5622
rs11220462	11	126243952	A	G	115.892	0.032	0.003	1.10E-28	0.036	0.032	0.2723
rs1122208	15	58623525	C	T	49.738	-0.015	0.002	3.40E-13	0.023	0.026	0.3675
rs11226108	11	103870755	C	G	56.080	-0.020	0.003	1.50E-14	0.050	0.033	0.1272
rs112354933	19	11446031	C	G	37.399	-0.035	0.006	1.50E-09	0.099	0.091	0.2764
rs112849259	19	45397307	T	C	635.389	0.162	0.006	6.30E-147	0.094	0.086	0.2778
rs112853430	9	107585213	T	C	59.095	0.041	0.005	2.60E-15	-0.003	0.088	0.9718
rs1132990	19	50028163	G	A	49.073	0.018	0.003	4.30E-12	-0.033	0.034	0.3346
rs113857915	2	43938274	T	C	51.069	-0.055	0.008	1.20E-14	-0.224	0.130	0.0840001
rs114245489	2	158485075	T	G	38.616	0.027	0.004	1.80E-10	0.029	0.068	0.6745
rs114303452	4	3449915	G	A	39.721	0.060	0.009	3.10E-12	-0.295	0.247	0.2317
rs11550348	19	46387344	A	G	135.653	-0.036	0.003	4.20E-34	-0.023	0.045	0.614899
rs11591147	1	55505647	T	G	1713.975	-0.327	0.008	1.00E-200	0.010	0.069	0.8843
rs11601507	11	5701074	A	C	39.089	0.026	0.004	9.10E-10	-0.029	0.047	0.5319
rs116089584	6	160962370	T	G	50.604	-0.045	0.006	1.40E-14	-0.027	0.080	0.7356
rs11636917	15	63793873	C	T	57.672	0.016	0.002	6.30E-16	0.018	0.026	0.4823
rs116529593	6	32409400	G	T	35.787	-0.071	0.012	4.50E-09	0.049	0.172	0.7784
rs116669880	1	109529092	T	C	32.722	-0.062	0.011	2.30E-08	-0.217	0.295	0.462701

rs116863821	15	57573483	G	T	28.727	0.035	0.007	3.20E-08	0.080	0.107	0.4564
rs117139027	8	29024943	A	G	54.323	-0.059	0.008	7.40E-15	0.118	0.217	0.5863
rs118163475	19	11035384	T	C	33.375	0.057	0.010	6.00E-09	-0.086	0.101	0.3953
rs11821808	11	75451187	T	C	49.174	-0.025	0.004	1.50E-12	-0.001	0.041	0.9791
rs11881101	19	45530552	G	A	70.772	-0.022	0.003	1.40E-17	-0.001	0.035	0.9734
rs11905831	20	62903550	G	A	43.626	0.017	0.003	2.90E-13	-0.027	0.037	0.4692
rs11920368	3	129275403	A	C	38.274	0.019	0.003	2.80E-11	-0.096	0.049	0.0478597
rs12046278	1	10799577	C	T	53.833	-0.016	0.002	1.40E-14	0.065	0.026	0.0116001
rs12162782	22	50853626	G	T	36.735	0.013	0.002	6.30E-10	-0.036	0.026	0.1559
rs12208357	6	160543148	T	C	172.218	0.053	0.004	1.50E-40	-0.031	0.054	0.5606
rs12336893	9	2632365	G	T	31.432	-0.025	0.005	1.90E-09	-0.064	0.071	0.366
rs12354278	1	154243457	T	A	23.831	0.015	0.003	4.00E-08	-0.003	0.045	0.9505
rs12444979	16	19933600	T	C	30.824	0.016	0.003	3.90E-08	0.010	0.038	0.7915
rs12452315	17	45479446	C	A	133.595	0.024	0.002	2.40E-32	0.025	0.025	0.3203
rs12478327	2	20374249	C	A	158.071	-0.026	0.002	2.80E-36	0.015	0.025	0.5469
rs1250259	2	216300482	A	T	36.030	0.014	0.002	1.20E-09	-0.022	0.031	0.4735
rs12577276	11	61632310	G	A	33.439	-0.041	0.007	1.90E-09	-0.046	0.061	0.4501
rs1260326	2	27730940	C	T	554.432	-0.050	0.002	9.10E-128	-0.137	0.027	2.48E-07
rs12607340	18	47151206	T	C	34.107	0.037	0.006	9.30E-10	0.013	0.046	0.772499
rs12666989	7	100486754	C	G	65.879	0.022	0.003	1.30E-16	0.036	0.031	0.2511
rs12718462	11	116705719	C	T	82.179	-0.037	0.004	9.80E-23	-0.103	0.048	0.0325702
rs12903114	15	64161184	T	C	28.322	0.011	0.002	1.50E-08	-0.002	0.027	0.9487
rs12916	5	74656539	C	T	865.517	0.062	0.002	5.11E-199	0.023	0.025	0.3713
rs12968116	18	55322502	T	C	31.147	0.018	0.003	1.00E-08	-0.038	0.039	0.33
rs13107325	4	103188709	T	C	105.303	-0.041	0.004	4.10E-24	0.046	0.108	0.668399
rs13225064	7	100717373	C	G	32.159	-0.015	0.003	3.20E-08	-0.031	0.035	0.3707
rs13289095	9	131466489	T	G	52.661	-0.021	0.003	7.10E-13	-0.018	0.041	0.6705
rs13387930	2	24414400	G	A	32.934	0.013	0.002	7.30E-09	-0.038	0.028	0.1832

rs13391398	2	219282429	A	G	29.015	-0.012	0.002	6.80E-09	0.034	0.027	0.1992
rs138354	22	41272143	C	T	35.990	-0.012	0.002	5.30E-09	-0.021	0.025	0.4173
rs140970775	1	161503013	G	A	33.119	0.023	0.004	7.20E-10	-0.064	0.054	0.236
rs142972972	17	7480325	C	A	41.932	0.053	0.008	7.00E-11	0.001	0.169	0.9967
rs143706998	2	44001372	C	A	67.181	-0.086	0.010	3.30E-17	0.042	0.181	0.8167
rs145248155	12	133295422	T	C	33.659	0.044	0.008	4.00E-08	-0.216	0.143	0.1309
rs1458038	4	81164723	T	C	56.748	-0.017	0.002	7.30E-13	-0.048	0.027	0.0736801
rs147211387	19	45261755	A	G	47.858	0.059	0.009	6.60E-14	0.070	0.152	0.645601
rs1501908	5	156398169	C	G	352.670	0.040	0.002	1.80E-80	-0.054	0.027	0.04348
rs1521516	12	51055708	T	C	86.348	-0.020	0.002	5.80E-18	-0.002	0.027	0.934
rs1524776	7	17872093	A	C	33.014	-0.012	0.002	1.10E-08	-0.023	0.026	0.3651
rs1537234	1	110279821	A	C	39.371	-0.013	0.002	3.50E-11	-0.003	0.026	0.9048
rs1564348	6	160578860	C	T	150.807	0.034	0.003	2.90E-38	-0.009	0.036	0.8028
rs1678299	4	8228627	C	G	33.149	0.014	0.002	1.00E-08	0.033	0.027	0.2241
rs16926246	10	71093392	T	C	62.913	-0.024	0.003	2.90E-15	0.053	0.049	0.2813
rs16979595	19	45477381	A	G	105.455	0.029	0.003	8.60E-25	-0.084	0.032	0.00809096
rs17050272	2	121306440	A	G	76.368	-0.018	0.002	3.90E-18	0.060	0.025	0.0184701
rs17248748	19	11206040	T	C	42.592	-0.053	0.008	2.90E-11	0.028	0.101	0.783201
rs17347726	19	44934842	C	G	159.591	-0.041	0.003	3.70E-37	0.077	0.049	0.114
rs1736176	19	2790394	A	G	47.555	0.016	0.002	4.90E-12	-0.011	0.027	0.6781
rs17369400	4	154224048	G	A	38.424	-0.021	0.003	1.40E-10	0.033	0.037	0.3766
rs174550	11	61571478	C	T	470.213	-0.047	0.002	1.50E-112	-0.003	0.026	0.9126
rs1748195	1	63049593	G	C	694.511	-0.056	0.002	6.90E-160	-0.015	0.029	0.595701
rs17512204	2	118732831	A	G	84.587	-0.034	0.004	7.20E-20	-0.001	0.048	0.9838
rs17580	14	94847262	A	T	44.964	0.033	0.005	2.60E-11	0.195	0.133	0.1428
rs17657174	22	30731387	G	C	34.848	-0.015	0.002	2.40E-10	0.016	0.034	0.637801

rs17666927	16	72143414	A	G	80.469	-0.063	0.007	1.90E-19	-0.035	0.146	0.8128
rs17789218	6	100600097	C	T	47.081	-0.016	0.002	2.40E-12	0.035	0.031	0.257
rs1795240	1	171091875	G	A	43.614	-0.014	0.002	8.60E-11	0.006	0.025	0.8276
rs1800562	6	26093141	A	G	136.385	-0.049	0.004	7.20E-32	-0.050	0.069	0.467
rs1800588	15	58723675	T	C	638.631	0.063	0.002	9.20E-145	0.058	0.029	0.0471596
rs1800961	20	43042364	T	C	261.746	-0.098	0.006	9.90E-63	0.019	0.061	0.761001
rs1801689	17	64210580	C	A	69.181	0.050	0.006	1.20E-17	-0.041	0.131	0.757
rs18742906<br>4	19	19380513	G	A	247.973	-0.147	0.009	4.60E-58	-0.034	0.059	0.5665
rs1883025	9	107664301	T	C	448.792	-0.050	0.002	5.30E-103	-0.005	0.032	0.8842
rs1902023	4	69536084	C	A	46.611	0.014	0.002	6.00E-13	0.066	0.025	0.00919009
rs198846	6	26107463	G	A	49.927	0.021	0.003	5.00E-14	-0.090	0.040	0.0243501
rs2000999	16	72108093	A	G	315.994	0.050	0.003	1.60E-75	-0.013	0.032	0.6883
rs2062251	19	58715682	T	G	37.164	-0.014	0.002	1.70E-10	-0.021	0.032	0.5106
rs2068888	10	94839642	A	G	63.670	-0.016	0.002	1.50E-16	-0.002	0.025	0.9318
rs207154	1	55781281	C	T	36.487	-0.022	0.004	4.60E-11	-0.067	0.043	0.1212
rs2071920	6	35216296	T	C	39.996	-0.017	0.003	4.20E-10	0.005	0.036	0.8911
rs2073547	7	44582331	G	A	156.811	0.033	0.003	1.40E-38	0.019	0.027	0.4797
rs2075650	19	45395619	G	A	2038.635	0.132	0.003	1.00E-200	-0.101	0.034	0.00270402
rs2081687	8	59388565	C	T	224.020	-0.032	0.002	3.90E-53	-0.026	0.026	0.3161
rs2109505	7	87079406	A	T	31.928	-0.015	0.003	7.80E-09	-0.009	0.032	0.769501
rs2143877	20	39092895	G	A	62.526	-0.017	0.002	8.80E-16	0.002	0.031	0.9528
rs2207132	20	39142516	A	G	271.031	0.094	0.006	1.20E-65	-0.104	0.051	0.0431698
rs2235147	22	35664327	C	T	28.485	-0.012	0.002	2.80E-08	0.021	0.026	0.4227
rs2236510	20	62368804	T	C	48.102	0.017	0.002	1.50E-13	0.001	0.032	0.9778
rs2241340	2	169828325	G	A	103.334	-0.022	0.002	2.00E-28	0.004	0.025	0.8821
rs2255437	16	83988886	G	A	48.975	-0.014	0.002	3.50E-15	0.049	0.027	0.0731796
rs2276133	11	65562257	A	G	36.329	0.014	0.002	3.50E-08	-0.047	0.029	0.1121
rs2277862	20	34152782	T	C	86.731	-0.028	0.003	3.90E-22	0.009	0.044	0.8385
rs2278426	19	11350488	T	C	191.192	-0.078	0.006	4.30E-45	0.013	0.051	0.8053
rs2278921	4	69362301	G	T	65.356	0.018	0.002	5.70E-17	0.016	0.025	0.522001

rs2288153	7	6461534	T	C	72.041	0.021	0.002	1.50E-18	-0.086	0.029	0.00263797
rs2290771	17	73844748	G	A	28.503	-0.012	0.002	5.20E-09	0.129	0.030	2.11E-05
rs2295033	20	47610896	T	C	34.441	0.013	0.002	2.40E-10	0.023	0.028	0.4089
rs2298624	18	47429022	T	C	38.024	0.019	0.003	4.60E-10	-0.068	0.033	0.04341
rs2302209	19	18324329	T	C	40.281	0.014	0.002	1.90E-10	-0.023	0.029	0.4308
rs2305407	3	160130110	A	G	38.078	-0.013	0.002	7.70E-09	-0.020	0.026	0.4283
rs2465520	2	204304214	A	G	39.976	-0.021	0.003	1.30E-10	-0.013	0.043	0.7669
rs2526385	3	50181135	G	T	40.460	0.016	0.003	7.80E-12	-0.028	0.029	0.3492
rs2539980	2	63208460	C	T	68.720	0.018	0.002	6.40E-17	-0.005	0.026	0.8607
rs2618566	20	17844684	T	G	116.060	-0.023	0.002	9.10E-30	-0.002	0.027	0.954
rs2642438	1	220970028	G	A	196.817	0.032	0.002	4.80E-47	-0.041	0.028	0.1395
rs2705455	4	156502857	G	A	37.142	-0.013	0.002	7.10E-11	0.037	0.026	0.1498
rs2706379	5	131805735	T	C	36.247	-0.016	0.003	5.20E-10	-0.015	0.031	0.6218
rs2737229	8	116648565	C	A	79.899	-0.020	0.002	3.40E-21	-0.029	0.026	0.276
rs2738447	19	11227480	C	A	312.133	0.037	0.002	1.30E-72	0.005	0.026	0.8622
rs2777799	9	107559059	A	G	72.805	0.029	0.003	4.30E-18	0.014	0.044	0.7487
rs2792751	10	113940329	C	T	116.630	-0.025	0.002	1.60E-30	0.032	0.027	0.2337
rs2802954	1	235015126	C	A	31.505	-0.013	0.002	6.20E-09	-0.020	0.026	0.4394
rs28403550	3	122251838	A	C	46.177	0.019	0.003	3.40E-11	-0.022	0.037	0.5555
rs2854300	19	46274392	C	G	102.374	-0.053	0.005	7.80E-24	-0.071	0.056	0.2057
rs2862954	10	101912064	C	T	83.363	0.019	0.002	2.80E-21	-0.009	0.026	0.717799
rs2875973	8	61476709	T	C	44.891	0.014	0.002	1.30E-11	0.023	0.026	0.363
rs2905880	17	29546175	C	T	55.532	0.017	0.002	1.10E-13	-0.034	0.027	0.2064
rs2927437	19	45241638	G	A	251.511	-0.041	0.003	7.00E-58	-0.003	0.030	0.92
rs2928619	8	6569927	T	C	39.859	0.014	0.002	1.90E-11	0.040	0.027	0.1367
rs3010271	5	71947654	G	A	53.623	-0.018	0.003	1.50E-13	0.036	0.031	0.2476
rs3021523	9	100616583	C	T	27.144	0.012	0.002	1.60E-08	-0.045	0.032	0.1579
rs33918808	9	107579632	G	C	31.788	0.035	0.006	1.40E-08	-0.069	0.084	0.4125
rs34752362	14	75232306	A	G	46.734	-0.014	0.002	3.70E-13	-0.021	0.026	0.4355
rs34862454	15	75101530	T	C	51.530	0.016	0.002	1.10E-13	-0.085	0.025	0.000867601
rs35242582	6	32600057	G	A	55.200	0.022	0.003	1.10E-15	-0.032	0.031	0.3033

rs35853021	15	58680643	T	G	612.697	0.053	0.002	1.50E-139	-0.024	0.026	0.3445
rs35882350	12	623129	G	A	32.436	0.013	0.002	3.20E-08	0.049	0.028	0.0881191
rs35939242	3	12301893	C	A	87.886	-0.020	0.002	1.20E-23	0.013	0.028	0.6483
rs36098083	2	137073291	T	C	27.133	-0.011	0.002	4.10E-08	-0.007	0.025	0.777401
rs364585	20	12962718	G	A	39.041	0.013	0.002	1.40E-10	-0.049	0.027	0.0631495
rs3732359	3	119536429	A	G	71.639	-0.021	0.002	3.50E-18	0.001	0.027	0.9851
rs3735400	7	36438709	G	C	25.723	-0.016	0.003	3.80E-08	-0.097	0.040	0.0167402
rs3737088	6	160553165	T	C	29.596	-0.028	0.005	1.70E-08	0.029	0.075	0.6962
rs3738621	1	235110784	G	A	56.605	-0.019	0.002	3.70E-15	0.006	0.034	0.8702
rs3745245	19	10676423	A	G	42.141	0.015	0.002	1.30E-11	-0.012	0.030	0.6857
rs3748176	9	117124731	A	G	28.309	0.011	0.002	2.70E-08	-0.024	0.025	0.3432
rs3756772	6	116325142	T	C	117.760	0.023	0.002	2.50E-28	0.032	0.025	0.2104
rs3764261	16	56993324	A	C	399.645	0.044	0.002	7.70E-92	0.017	0.028	0.5394
rs3764613	19	46896217	G	A	34.869	-0.012	0.002	2.40E-08	0.027	0.025	0.2815
rs3771240	2	20412686	C	T	40.805	-0.013	0.002	8.10E-10	0.018	0.028	0.5278
rs3800461	6	34616322	C	G	119.220	-0.035	0.003	4.00E-29	-0.004	0.033	0.9104
rs3808348	7	1028448	T	C	45.750	-0.017	0.003	2.10E-12	-0.045	0.028	0.1087
rs3826909	19	45721596	A	G	68.645	0.030	0.004	2.70E-17	-0.010	0.042	0.82
rs40245	7	21470536	T	A	75.145	0.019	0.002	7.90E-20	-0.002	0.026	0.9557
rs41267805	6	160921969	A	G	55.727	-0.040	0.005	3.60E-14	0.018	0.047	0.7004
rs4129767	17	76403984	A	G	141.534	0.024	0.002	2.70E-34	0.051	0.026	0.0507902
rs41313290	1	110155275	A	G	28.462	-0.051	0.010	9.20E-09	-0.321	0.249	0.1974
rs4149311	9	107588777	T	C	139.519	0.037	0.003	1.90E-33	0.052	0.044	0.232
rs4245791	2	44074431	T	C	605.362	-0.054	0.002	4.20E-140	-0.042	0.031	0.166
rs440677	19	11285390	A	G	54.920	-0.016	0.002	4.50E-15	0.007	0.025	0.7893
rs4506565	10	114756041	T	A	31.053	-0.012	0.002	1.30E-08	-0.001	0.030	0.9821
rs4530754	5	122855416	A	G	68.404	0.017	0.002	2.80E-18	-0.037	0.025	0.1404
rs4565995	12	100661828	C	T	35.194	0.021	0.004	3.00E-09	-0.030	0.035	0.3958
rs4661712	1	16493225	A	G	37.796	0.013	0.002	3.40E-11	0.032	0.026	0.224
rs4712999	6	26466234	G	A	32.509	-0.018	0.003	3.50E-09	-0.011	0.035	0.7616
rs471705	1	55521242	G	T	344.751	0.040	0.002	2.90E-79	-0.034	0.026	0.1865

rs4722551	7	25991826	C	T	48.968	0.020	0.003	6.70E-13	-0.051	0.031	0.0981499
rs4752805	11	48018355	G	A	77.641	0.021	0.002	1.10E-20	0.027	0.030	0.3747
rs4841132	8	9183596	G	A	460.299	0.081	0.004	4.60E-108	0.035	0.038	0.354
rs4850047	2	3634753	C	T	52.976	-0.022	0.003	8.20E-14	0.059	0.028	0.0344302
rs4921914	8	18272438	T	C	97.998	-0.024	0.002	8.90E-25	-0.040	0.029	0.1726
rs4939883	18	47167214	C	T	295.333	0.046	0.003	1.50E-68	-0.050	0.033	0.1332
rs505151	1	55529187	A	G	135.713	-0.068	0.006	3.70E-34	0.023	0.057	0.6842
rs507666	9	136149399	A	G	441.759	0.056	0.003	2.90E-103	-0.045	0.031	0.1551
rs526936	1	234852204	A	G	332.387	0.037	0.002	8.40E-75	-0.040	0.025	0.1141
rs532315709	19	10531314	A	G	60.744	-0.054	0.007	1.60E-15	0.308	0.109	0.004495
rs55714927	17	7080316	T	C	89.936	-0.025	0.003	6.60E-23	-0.009	0.029	0.7485
rs55784804	17	7536460	T	G	28.925	0.019	0.004	6.50E-10	0.005	0.048	0.9211
rs55791371	19	11188153	C	A	2567.825	-0.160	0.003	1.00E-200	0.086	0.042	0.0427199
rs55865386	2	20984945	T	C	73.945	0.044	0.005	1.30E-18	-0.005	0.075	0.9453
rs55885610	17	27579011	T	C	37.083	-0.034	0.006	3.20E-10	-0.054	0.076	0.4804
rs562338	2	21288321	G	A	1316.047	0.096	0.003	1.00E-200	-0.003	0.032	0.9163
rs56383182	2	135608211	G	A	62.711	0.017	0.002	2.90E-17	0.013	0.026	0.6291
rs5754217	22	21939675	T	G	87.428	-0.024	0.003	1.40E-21	0.044	0.027	0.1036
rs581080	9	15305378	C	G	88.755	0.025	0.003	1.10E-20	-0.025	0.036	0.4895
rs58148580	4	124758773	T	C	36.113	0.020	0.003	2.40E-09	0.035	0.040	0.3807
rs58542926	19	19379549	T	C	1002.197	-0.125	0.004	1.00E-200	-0.020	0.052	0.7052
rs5880	16	57015091	C	G	147.683	-0.056	0.005	1.10E-34	0.019	0.081	0.8139
rs5888	12	125284748	G	A	79.518	-0.018	0.002	2.40E-18	0.027	0.026	0.2979
rs593818	19	15807884	G	A	39.560	0.013	0.002	3.00E-10	-0.011	0.026	0.6761
rs6072355	20	40008553	C	G	52.352	0.017	0.002	1.20E-13	-0.014	0.026	0.5953
rs608511	1	109776170	T	C	43.400	-0.015	0.002	1.00E-12	-0.050	0.028	0.0746707
rs6115094	20	25210827	G	A	38.665	0.013	0.002	3.10E-10	0.054	0.025	0.0337699
rs6127015	20	52540606	C	T	31.010	0.011	0.002	5.00E-10	-0.027	0.026	0.2924
rs6129752	20	39710496	G	A	76.952	0.022	0.002	1.20E-19	0.021	0.031	0.5034
rs61745773	19	11485392	T	C	62.755	-0.072	0.009	3.60E-16	-0.087	0.125	0.487

rs61884271	11	46654404	C	T	33.599	-0.016	0.003	3.30E-10	-0.008	0.038	0.8454
rs62117160	19	45232161	A	G	2727.393	-0.255	0.005	1.00E-200	-0.027	0.077	0.7274
rs62119263	19	45125197	C	T	58.149	-0.020	0.003	1.10E-14	-0.006	0.032	0.8477
rs62120572	19	45213752	T	C	33.210	0.028	0.005	7.00E-09	-0.022	0.058	0.710201
rs62292953	3	132202449	A	T	32.541	-0.017	0.003	3.10E-09	0.008	0.034	0.8208
rs6416553	15	91135411	T	C	31.347	-0.016	0.003	1.90E-08	-0.087	0.035	0.0131401
rs6474359	8	41549194	C	T	33.617	-0.031	0.005	1.50E-09	-0.019	0.098	0.8461
rs6475606	9	22081850	T	C	96.206	-0.020	0.002	4.30E-25	-0.014	0.026	0.580301
rs6482423	10	18725053	C	T	32.806	0.012	0.002	1.80E-08	0.046	0.028	0.1015
rs6499163	16	68243486	T	G	29.632	0.015	0.003	1.90E-08	-0.046	0.033	0.1574
rs653178	12	112007756	T	C	268.535	0.034	0.002	1.20E-64	-0.082	0.026	0.001327
rs6603979	1	92979354	G	A	66.580	0.021	0.003	2.00E-17	-0.023	0.030	0.443
rs66476925	7	21611399	C	G	172.030	0.033	0.003	1.10E-40	0.010	0.031	0.7565
rs6734238	2	113841030	G	A	41.645	-0.013	0.002	1.30E-11	-0.043	0.028	0.1179
rs6756943	2	64926764	A	G	47.833	0.015	0.002	3.70E-13	-0.004	0.030	0.8947
rs676388	19	49211969	C	T	225.273	0.031	0.002	3.30E-52	0.058	0.026	0.0245901
rs6785233	3	170756985	G	T	34.246	0.022	0.004	1.20E-08	0.110	0.067	0.1005
rs679899	2	21250914	A	G	376.203	-0.040	0.002	1.80E-88	0.027	0.026	0.2983
rs6831256	4	3473139	G	A	68.119	0.017	0.002	1.70E-18	-0.026	0.026	0.3247
rs6987702	8	126504726	C	T	224.180	0.035	0.002	8.00E-55	0.033	0.027	0.2127
rs7015	7	97920623	G	A	33.427	0.015	0.003	2.40E-09	0.105	0.035	0.00265901
rs7124487	11	2988323	T	C	42.641	-0.017	0.003	3.80E-11	0.014	0.039	0.7134
rs7157785	14	64235556	T	G	40.763	0.018	0.003	4.00E-11	-0.023	0.037	0.5454
rs7186852	16	30635659	G	A	31.397	0.012	0.002	1.30E-08	-0.012	0.027	0.6417
rs7249753	19	11192226	C	T	108.815	0.045	0.004	1.40E-26	0.089	0.061	0.1489
rs7251440	19	11259754	A	T	25.285	0.045	0.009	3.90E-08	-0.007	0.085	0.9352
rs7254892	19	45389596	A	G	3046.829	-0.321	0.006	1.00E-200	-0.106	0.085	0.2134
rs72631343	17	67191270	G	C	81.593	-0.028	0.003	1.00E-20	0.051	0.036	0.1561
rs72660536	1	55486751	A	G	91.841	0.028	0.003	2.80E-22	-0.002	0.044	0.9561
rs72669514	1	63194030	T	C	43.313	0.032	0.005	6.50E-12	-0.037	0.061	0.546
rs72836561	17	41926126	T	C	65.451	-0.048	0.006	9.10E-17	-0.085	0.072	0.2398

rs72905574	1	55433530	T	C	38.137	-0.022	0.004	9.70E-11	-0.021	0.048	0.654501
rs7298565	12	109937534	A	G	54.890	0.015	0.002	1.70E-14	0.045	0.025	0.0769999
rs73078112	20	5547384	T	C	31.184	0.036	0.006	1.50E-08	0.132	0.134	0.3218
rs738409	22	44324727	G	C	31.634	-0.015	0.003	2.10E-09	-0.159	0.030	1.58E-07
rs74607435	19	45235700	C	T	113.324	-0.049	0.005	3.90E-26	-0.136	0.081	0.0929501
rs74617384	6	160997118	T	A	415.538	0.078	0.004	1.50E-94	-0.001	0.061	0.9859
rs7522956	1	224585958	C	A	47.306	-0.017	0.003	4.60E-12	0.016	0.030	0.595
rs7528419	1	109817192	G	A	1734.879	-0.102	0.002	1.00E-200	-0.012	0.031	0.6927
rs7543281	1	234743237	A	G	51.283	0.021	0.003	7.80E-14	0.013	0.036	0.725301
rs75482042	3	12572184	T	G	43.800	-0.023	0.003	1.10E-12	0.006	0.050	0.9092
rs75562913	16	69176856	C	A	45.014	-0.031	0.005	3.70E-12	-0.027	0.037	0.4656
rs75689126	9	107366346	G	T	29.417	-0.032	0.006	4.00E-08	-0.016	0.105	0.8779
rs76222503	8	9195074	A	C	52.800	0.038	0.005	3.50E-13	-0.057	0.079	0.4748
rs7640978	3	32533010	T	C	79.892	-0.032	0.004	3.00E-18	-0.126	0.046	0.00628102
rs76786359	10	52380642	G	A	37.452	0.042	0.007	3.70E-09	0.058	0.116	0.6163
rs77370158	2	21023265	G	A	43.305	0.024	0.004	1.80E-10	0.016	0.061	0.7951
rs7748291	6	37039564	T	C	48.814	-0.014	0.002	4.80E-13	-0.008	0.026	0.753701
rs77542162	17	67081278	G	A	241.567	0.109	0.007	5.40E-59	-0.217	0.163	0.182
rs77704739	5	52080909	C	T	47.041	-0.036	0.005	9.70E-12	0.033	0.073	0.6513
rs77960347	18	47109955	G	A	374.749	0.176	0.009	6.90E-88	-0.067	0.151	0.656299
rs78073763	19	45649838	G	T	43.584	-0.024	0.004	1.60E-11	0.041	0.053	0.4378
rs78620885	19	45591084	T	C	40.286	0.038	0.006	5.90E-11	0.034	0.058	0.5611
rs78635447	2	43561134	A	G	32.685	0.028	0.005	5.10E-09	0.218	0.115	0.0580297
rs78946096	3	132188163	G	A	86.531	-0.041	0.004	4.30E-21	0.076	0.052	0.1428
rs7920112	10	17259863	C	T	67.825	0.017	0.002	2.40E-17	-0.012	0.026	0.6384
rs7921838	10	5247701	A	G	45.308	-0.019	0.003	4.50E-11	-0.018	0.036	0.6148
rs7941030	11	122522375	C	T	97.876	0.021	0.002	1.20E-24	-0.031	0.026	0.2372
rs79514711	1	63323814	T	C	33.902	-0.029	0.005	3.50E-09	-0.026	0.071	0.719701
rs79783247	5	74316685	G	A	49.470	0.057	0.008	1.30E-11	-0.077	0.247	0.755
rs79860022	10	70968048	T	C	27.354	0.030	0.006	1.10E-08	-0.043	0.134	0.747699
rs799157	7	73020301	C	T	31.027	-0.028	0.005	4.60E-09	-0.162	0.085	0.0552599

rs80101730	8	9856358	C	T	30.302	-0.035	0.006	8.40E-09	-0.026	0.059	0.654501
rs8017377	14	24883887	A	G	64.201	0.017	0.002	1.30E-15	-0.018	0.026	0.4956
rs826682	2	109232388	C	A	39.687	0.018	0.003	3.50E-10	0.022	0.032	0.498101
rs8302	2	44036913	C	T	34.516	0.016	0.003	2.10E-10	0.012	0.033	0.7219
rs871841	17	8216468	C	T	31.173	0.012	0.002	3.10E-09	0.009	0.025	0.7199
rs887829	2	234668570	T	C	52.855	-0.016	0.002	1.60E-13	0.070	0.026	0.00679297
rs903503	17	37829571	A	C	36.674	0.013	0.002	1.50E-10	-0.068	0.027	0.0123501
rs9352675	6	79612800	A	G	25.863	-0.011	0.002	2.60E-08	0.018	0.025	0.465
rs9370867	6	16145325	G	A	89.678	-0.020	0.002	1.30E-23	0.030	0.025	0.2408
rs9375459	6	127147704	T	C	43.088	-0.014	0.002	7.00E-12	-0.023	0.025	0.3603
rs9376090	6	135411228	C	T	67.618	-0.019	0.002	1.80E-18	0.048	0.027	0.0696899
rs9534262	13	32936646	C	T	51.107	-0.015	0.002	8.70E-13	0.016	0.025	0.5229
rs9577924	13	114545317	G	A	115.958	0.025	0.002	7.80E-29	0.041	0.029	0.1628
rs964184	11	116648917	C	G	567.652	-0.072	0.003	3.10E-136	-0.032	0.036	0.3714
rs9668810	12	26426420	C	T	29.807	-0.013	0.002	9.90E-09	0.024	0.028	0.3974
rs970548	10	46013277	C	A	57.820	0.018	0.002	6.10E-16	0.024	0.028	0.3905
rs9825431	3	58406694	C	G	73.805	-0.032	0.004	4.30E-18	0.061	0.046	0.1881
rs9832727	3	142649110	G	C	54.728	-0.016	0.002	1.30E-13	-0.046	0.027	0.0837394
rs9907571	17	40448844	A	G	30.187	0.012	0.002	4.90E-08	0.013	0.030	0.6536
rs9916193	17	18057215	G	C	32.535	0.015	0.003	6.40E-09	-0.034	0.036	0.3513
rs9923575	16	72230112	T	C	55.148	-0.019	0.003	6.00E-15	0.041	0.032	0.2069

SNP	Chr	Position	Effect Allele	Other Allele	F	Association with exposure			Association with Gout		
						Beta	SE	*P*	Beta	SE	*P*
**HDL-C**											
rs10028284	4	89752913	T	A	45.478	0.017	0.003	1.50E-11	-0.102	0.029	0.000472096
rs10031010	4	120035180	A	G	31.355	0.014	0.002	2.10E-08	0.018	0.030	0.5466
rs10095103	8	9226095	C	T	180.490	-0.026	0.002	3.80E-41	0.008	0.027	0.759
rs10118245	9	139979592	T	C	44.395	0.014	0.002	2.70E-11	-0.015	0.027	0.5703

rs1012306	12	101888063	T	C	30.918	0.011	0.002	2.70E-08	-0.038	0.025	0.1345
rs1013339	22	38974680	T	C	68.525	-0.017	0.002	1.30E-16	-0.049	0.027	0.0636693
rs10233430	7	1051664	C	T	113.027	-0.020	0.002	2.10E-26	-0.038	0.025	0.1376
rs10281161	7	1181356	T	C	57.238	0.017	0.002	3.90E-14	0.019	0.031	0.5354
rs1043897	1	230416399	T	G	105.692	0.020	0.002	8.60E-25	-0.007	0.026	0.8033
rs1045241	5	118729286	T	C	57.995	0.016	0.002	2.60E-14	-0.011	0.027	0.679599
rs1047891	2	211540507	A	C	85.593	-0.019	0.002	2.20E-20	-0.048	0.027	0.0761097
rs10504477	8	71338185	C	T	60.877	-0.015	0.002	6.10E-15	-0.004	0.026	0.892
rs10513801	3	185822353	G	T	119.881	-0.030	0.003	6.70E-28	-0.010	0.042	0.8061
rs1055582	4	39700173	T	C	53.935	0.014	0.002	2.10E-13	-0.022	0.026	0.3947
rs10750766	11	65473798	A	C	78.219	-0.019	0.002	9.20E-19	0.069	0.029	0.01595
rs10773128	12	125442924	C	T	33.634	0.012	0.002	6.70E-09	0.001	0.027	0.9689
rs10774439	12	6731818	A	G	68.240	0.021	0.002	1.40E-16	0.011	0.030	0.710699
rs10774579	12	121405210	C	T	72.806	-0.016	0.002	1.40E-17	0.007	0.025	0.7873
rs1077835	15	58723426	G	A	2618.180	0.118	0.002	1.00E-200	0.068	0.029	0.0184999
rs10786114	10	95309022	T	C	68.604	0.024	0.003	1.20E-16	0.015	0.036	0.6853
rs10826337	10	61409469	A	G	33.843	-0.011	0.002	6.00E-09	0.014	0.026	0.5989
rs1083470	2	147871114	A	G	34.933	0.012	0.002	3.40E-09	0.026	0.026	0.303
rs1086056	3	154088411	G	T	39.264	-0.017	0.003	3.70E-10	0.027	0.033	0.4107
rs10883451	10	101924418	C	T	59.908	0.015	0.002	9.90E-15	-0.009	0.026	0.7199
rs10947506	6	34464881	A	G	46.885	-0.013	0.002	7.50E-12	0.000	0.026	0.9873
rs10950390	7	12224708	T	C	40.992	-0.015	0.002	1.50E-10	0.019	0.031	0.5364
rs10955991	8	121867780	C	T	94.214	-0.020	0.002	2.80E-22	0.015	0.027	0.5733
rs10980049	9	112563770	T	C	30.563	0.012	0.002	3.20E-08	-0.047	0.032	0.1401
rs11000468	10	74711376	T	C	31.859	0.013	0.002	1.70E-08	0.010	0.026	0.695
rs11009262	10	33448764	T	G	32.495	-0.023	0.004	1.20E-08	-0.030	0.053	0.574101
rs11021232	11	95320808	C	T	45.302	-0.017	0.002	1.70E-11	0.025	0.034	0.4643
rs11038864	11	46349103	A	G	54.586	-0.018	0.002	1.50E-13	-0.005	0.034	0.8833
rs11045171	12	20470199	G	A	139.046	0.028	0.002	4.30E-32	0.072	0.031	0.0193602
rs11057452	12	124589746	T	C	107.873	0.021	0.002	2.90E-25	-0.045	0.026	0.0821297
rs11082813	18	47831170	G	A	30.518	0.011	0.002	3.30E-08	0.017	0.027	0.537499

rs111606268	15	58788234	C	T	38.388	0.034	0.006	5.80E-10	0.234	0.094	0.0132501
rs111619038	15	57870164	T	A	33.504	0.023	0.004	7.10E-09	-0.023	0.047	0.6227
rs11170516	12	53752692	A	G	49.810	-0.019	0.003	1.70E-12	0.014	0.037	0.7171
rs111843362	16	57139069	T	C	74.979	0.038	0.004	4.80E-18	-0.004	0.045	0.9254
rs112001035	17	66823805	A	G	130.423	-0.047	0.004	3.30E-30	-0.094	0.046	0.0428598
rs11216020	11	116452961	C	G	75.262	0.017	0.002	4.10E-18	-0.035	0.030	0.2385
rs112259268	17	41874745	A	C	856.343	-0.169	0.006	3.00E-188	-0.089	0.073	0.2218
rs11239536	10	45978598	A	T	166.022	0.029	0.002	5.50E-38	0.031	0.028	0.2615
rs112519623	4	103184239	A	G	34.130	-0.045	0.008	5.20E-09	0.070	0.087	0.4194
rs11254464	10	17265447	C	T	43.661	0.013	0.002	3.90E-11	-0.006	0.026	0.8143
rs112771035	11	126225876	G	C	73.609	-0.032	0.004	9.50E-18	0.055	0.046	0.2354
rs1132274	20	17596155	A	C	68.079	-0.022	0.003	1.60E-16	-0.041	0.037	0.2643
rs113232777	9	107686078	C	T	37.747	0.040	0.007	8.10E-10	0.064	0.085	0.4472
rs113271699	11	116837481	A	C	76.801	0.070	0.008	1.90E-18	-0.142	0.090	0.1129
rs113629348	15	58545668	T	C	109.191	0.062	0.006	1.50E-25	0.029	0.123	0.8134
rs113740515	12	123199410	A	G	260.714	0.038	0.002	1.20E-58	-0.019	0.033	0.562
rs1138429	16	56942921	T	A	1131.522	-0.100	0.003	1.00E-200	0.048	0.051	0.3418
rs114165349	1	27021913	C	G	162.120	-0.081	0.006	3.90E-37	0.249	0.067	0.000189601
rs11474146	6	32596835	T	C	62.926	-0.039	0.005	2.10E-15	0.056	0.173	0.7465

0											
rs11546878	3	183976103	T	C	31.244	0.014	0.003	2.30E-08	-0.059	0.031	0.0588004
rs11550348	19	46387344	A	G	151.574	-0.036	0.003	7.90E-35	-0.023	0.045	0.614899
rs1155347	6	39146230	C	T	33.250	0.013	0.002	8.10E-09	-0.037	0.029	0.2061
rs115912456	5	82815158	G	A	34.398	0.028	0.005	4.50E-09	0.109	0.094	0.2463
rs116000952	6	32550803	G	T	39.573	0.025	0.004	3.20E-10	-0.083	0.139	0.5496
rs116006942	5	53405314	A	G	54.803	-0.030	0.004	1.30E-13	0.053	0.064	0.4089
rs116008080	16	67254841	A	G	33.673	0.036	0.006	6.50E-09	-0.089	0.077	0.25
rs11601507	11	5701074	A	C	46.528	-0.025	0.004	9.00E-12	-0.029	0.047	0.5319
rs11605837	11	68597886	T	G	73.131	-0.018	0.002	1.20E-17	-0.042	0.026	0.1035
rs11622947	14	98396293	T	C	30.974	-0.011	0.002	2.60E-08	0.003	0.025	0.9011
rs11640494	16	88029685	A	G	65.282	-0.015	0.002	6.50E-16	0.019	0.027	0.4934
rs11640954	16	56908884	A	G	797.388	0.067	0.002	2.00E-175	0.002	0.035	0.9664
rs11653260	17	66464683	G	A	33.462	-0.013	0.002	7.30E-09	-0.011	0.028	0.6897
rs11658786	17	37815899	A	G	227.343	0.031	0.002	2.30E-51	-0.070	0.027	0.0102101
rs11658872	17	17425069	T	C	63.720	-0.033	0.004	1.40E-15	0.011	0.044	0.7985
rs11664369	18	57739072	T	C	113.309	-0.023	0.002	1.80E-26	0.081	0.032	0.0107199
rs1168114	1	63156043	G	A	60.455	0.016	0.002	7.50E-15	-0.009	0.029	0.7533
rs116843064	19	8429323	A	G	888.329	0.206	0.007	3.40E-195	-0.129	0.078	0.0970689
rs11684509	2	227202252	A	G	66.549	0.019	0.002	3.40E-16	-0.011	0.033	0.7383
rs116862333	12	123346801	C	T	56.171	-0.048	0.006	6.60E-14	-0.012	0.104	0.9049
rs11687520	2	173930266	C	T	44.694	0.017	0.003	2.30E-11	0.003	0.036	0.9333
rs11688682	2	121347612	C	G	44.333	0.015	0.002	2.80E-11	-0.059	0.030	0.04735
rs116974927	8	19951598	C	G	312.912	0.159	0.009	5.10E-70	0.070	0.103	0.4963

rs117001569	8	19574920	G	C	57.591	0.080	0.010	3.20E-14	-0.027	0.092	0.7703
rs11709077	3	12336507	A	G	35.002	0.017	0.003	3.30E-09	-0.018	0.034	0.597301
rs117198528	18	47220558	G	T	49.812	0.054	0.008	1.70E-12	-0.082	0.096	0.3951
rs117230571	13	41689067	G	A	53.834	-0.027	0.004	2.20E-13	0.075	0.069	0.2768
rs117291242	11	380315	T	C	38.872	-0.032	0.005	4.50E-10	0.064	0.057	0.2585
rs11736681	4	103069256	A	G	32.550	0.015	0.003	1.20E-08	0.013	0.040	0.739301
rs117478682	16	56684157	T	C	31.106	-0.065	0.012	2.40E-08	-0.112	0.069	0.1038
rs117520516	15	59207747	C	T	45.642	-0.031	0.005	1.40E-11	0.187	0.112	0.0963696
rs117956669	8	19847645	G	T	44.813	-0.060	0.009	2.20E-11	-0.109	0.107	0.3066
rs118027010	8	121857366	T	C	50.729	0.055	0.008	1.10E-12	-0.027	0.084	0.7501
rs11883967	2	66673862	C	A	39.270	0.013	0.002	3.70E-10	-0.048	0.026	0.0695392
rs11938781	4	17924734	C	T	33.854	-0.015	0.003	5.90E-09	0.041	0.041	0.3111
rs11985475	8	25464656	T	C	42.572	0.013	0.002	6.80E-11	0.023	0.026	0.3907
rs12046972	1	219673143	C	T	59.839	-0.015	0.002	1.00E-14	0.023	0.026	0.3762
rs12049914	11	62369436	G	C	56.960	-0.015	0.002	4.40E-14	-0.077	0.029	0.00816996
rs12129540	1	224625334	T	C	34.995	-0.028	0.005	3.30E-09	-0.057	0.097	0.5593
rs12140153	1	62579891	T	G	35.160	0.020	0.003	3.00E-09	0.082	0.047	0.0803804
rs12146566	11	103871404	C	A	32.385	-0.014	0.002	1.30E-08	0.044	0.030	0.1403
rs12150914	19	47563418	C	T	58.512	-0.015	0.002	2.00E-14	-0.035	0.026	0.1725
rs1225053	3	131642852	C	T	48.162	-0.015	0.002	3.90E-12	0.030	0.029	0.3045
rs1229973	4	100287178	T	C	51.770	-0.014	0.002	6.20E-13	0.001	0.025	0.9626
rs12351518	9	19357812	C	G	47.240	0.021	0.003	6.30E-12	0.029	0.042	0.4936
rs1240820	6	16825137	A	G	39.395	0.013	0.002	3.50E-10	0.042	0.028	0.1313

rs12411732	10	113978850	A	G	124.437	-0.031	0.003	6.80E-29	-0.001	0.038	0.9822
rs1243337	11	117270818	C	A	69.471	-0.017	0.002	7.80E-17	0.036	0.027	0.1822
rs12461203	19	41764568	T	G	36.333	0.018	0.003	1.70E-09	0.034	0.036	0.3465
rs12485478	3	12351223	G	A	77.659	-0.052	0.006	1.20E-18	0.171	0.077	0.0257602
rs12516070	5	59391636	C	T	41.463	0.012	0.002	1.20E-10	-0.020	0.025	0.4366
rs1252424	12	67670041	G	A	37.592	0.012	0.002	8.70E-10	-0.004	0.026	0.8782
rs12575456	11	47326073	A	G	480.789	0.045	0.002	1.40E-106	0.012	0.027	0.658801
rs12592616	15	64165974	G	C	60.376	-0.016	0.002	7.80E-15	-0.009	0.026	0.728201
rs12628784	22	29912430	G	A	42.320	-0.015	0.002	7.80E-11	0.026	0.031	0.4063
rs12650112	4	99788480	T	C	59.484	0.016	0.002	1.20E-14	-0.006	0.026	0.8187
rs12686780	9	95382297	T	C	41.485	-0.016	0.003	1.20E-10	-0.014	0.031	0.6655
rs12692596	2	161265910	T	C	32.610	-0.011	0.002	1.10E-08	-0.036	0.027	0.179
rs1270076	15	83543605	G	A	39.365	0.014	0.002	3.50E-10	0.027	0.031	0.3941
rs12705595	7	109103912	A	G	31.080	0.011	0.002	2.50E-08	-0.006	0.026	0.8206
rs12713007	2	48484467	T	C	31.688	-0.011	0.002	1.80E-08	0.012	0.026	0.6565
rs1273584	16	57351403	A	T	93.702	-0.038	0.004	3.70E-22	0.065	0.058	0.2616
rs12740374	1	109817590	T	G	158.711	0.029	0.002	2.20E-36	-0.014	0.031	0.6607
rs12740811	1	171416256	G	A	31.950	-0.018	0.003	1.60E-08	-0.063	0.046	0.1727
rs12803463	11	58376120	A	G	46.112	0.024	0.003	1.10E-11	0.006	0.040	0.8715
rs12814794	12	26440698	A	G	45.044	0.015	0.002	1.90E-11	-0.019	0.028	0.4997
rs12821774	12	57964445	C	G	34.102	-0.025	0.004	5.20E-09	0.020	0.042	0.640801
rs12908761	15	58715647	G	A	54.776	0.024	0.003	1.40E-13	-0.020	0.043	0.6434
rs12909187	15	75457873	C	T	36.674	0.012	0.002	1.40E-09	-0.043	0.026	0.0979603
rs12926854	16	85951258	G	A	32.947	0.012	0.002	9.50E-09	0.031	0.030	0.3089
rs12928099	16	15150505	A	C	107.361	0.021	0.002	3.70E-25	-0.063	0.028	0.02512
rs12930744	16	71986545	A	G	50.140	0.015	0.002	1.40E-12	0.027	0.031	0.3895
rs12975319	19	3414088	A	G	42.537	-0.014	0.002	6.90E-11	0.011	0.028	0.6832
rs12986742	2	58975143	C	T	30.699	-0.011	0.002	3.00E-08	0.016	0.026	0.5298
rs12998038	2	42602387	T	C	36.461	0.013	0.002	1.60E-09	0.030	0.028	0.2811
rs13066793	3	87037543	G	A	43.983	0.022	0.003	3.30E-11	0.058	0.047	0.2129
rs13087167	3	185878140	C	G	71.380	0.017	0.002	2.90E-17	-0.010	0.026	0.702399

rs13097947	3	15847941	C	T	61.824	0.016	0.002	3.80E-15	-0.010	0.028	0.7341
rs13107325	4	103188709	T	C	491.410	-0.080	0.004	7.00E-109	0.046	0.108	0.668399
rs13111599	4	83917037	G	A	34.859	0.013	0.002	3.50E-09	0.007	0.029	0.8142
rs13137144	4	967445	A	G	72.698	0.016	0.002	1.50E-17	-0.001	0.026	0.9729
rs13144151	4	146403165	G	A	43.692	0.018	0.003	3.80E-11	0.032	0.035	0.357
rs1321602	6	153588258	T	C	33.033	-0.014	0.002	9.10E-09	0.008	0.030	0.7951
rs13235365	7	6456091	T	C	145.039	0.026	0.002	2.10E-33	-0.067	0.030	0.02243
rs13263073	8	105982216	C	A	45.629	-0.017	0.002	1.40E-11	-0.013	0.030	0.6718
rs13269725	8	72459889	G	A	54.135	-0.026	0.004	1.90E-13	0.019	0.067	0.782
rs13336936	16	56722572	T	C	114.344	0.059	0.006	1.10E-26	-0.025	0.064	0.690601
rs13379043	14	74250126	C	T	84.118	0.020	0.002	4.70E-20	-0.009	0.032	0.7678
rs13389219	2	165528876	T	C	203.074	0.028	0.002	4.50E-46	-0.029	0.027	0.2808
rs13402475	2	3640142	G	C	99.536	-0.025	0.002	1.90E-23	0.060	0.027	0.0266502
rs13796729 0	9	107666614	A	G	118.295	0.076	0.007	1.50E-27	0.142	0.108	0.191
rs13844429 0	11	56420281	A	G	36.760	-0.033	0.005	1.30E-09	0.014	0.067	0.8305
rs13903773 5	19	33805258	T	C	33.467	-0.026	0.004	7.20E-09	0.027	0.058	0.643
rs1395221	4	24626903	T	G	32.802	-0.011	0.002	1.00E-08	-0.022	0.026	0.3914
rs13956532 3	12	113671175	G	A	35.284	-0.030	0.005	2.90E-09	0.029	0.047	0.5409
rs13982805 3	3	195298892	C	T	32.231	-0.032	0.006	1.40E-08	-0.014	0.064	0.8252
rs14040578 3	17	41974768	C	G	69.398	-0.024	0.003	8.00E-17	-0.070	0.035	0.0436204
rs14106219 6	17	495327	A	G	61.166	-0.019	0.002	5.20E-15	0.029	0.037	0.421
rs1411432	9	16728532	C	A	32.389	-0.014	0.002	1.30E-08	0.041	0.033	0.2231
rs1412234	9	28410683	C	T	34.680	-0.012	0.002	3.90E-09	-0.024	0.026	0.3586
rs14124861	2	208494175	T	C	36.219	0.023	0.004	1.80E-09	-0.094	0.057	0.1034

0											
rs141469619	11	116714293	G	A	399.078	-0.203	0.010	8.70E-89	-0.343	0.203	0.0909997
rs141705406	16	68916181	T	C	30.463	0.027	0.005	3.40E-08	0.064	0.077	0.4067
rs141724276	16	56915407	T	C	80.803	0.071	0.008	2.50E-19	0.012	0.054	0.8217
rs1420192	7	36239398	T	C	37.524	-0.012	0.002	9.00E-10	-0.024	0.026	0.3551
rs142558755	16	56722274	A	C	262.141	0.067	0.004	5.90E-59	0.058	0.051	0.2472
rs142611813	16	57097274	T	C	43.400	-0.050	0.008	4.50E-11	-0.312	0.238	0.1905
rs1431659	8	73439070	G	A	35.696	0.013	0.002	2.30E-09	-0.041	0.029	0.1591
rs143395086	9	107654388	G	T	41.093	0.074	0.012	1.50E-10	-0.135	0.092	0.1423
rs143474489	11	50628094	A	T	53.869	0.048	0.007	2.10E-13	-0.198	0.222	0.3707
rs143786003	12	124695007	A	C	52.248	-0.038	0.005	4.90E-13	-0.192	0.070	0.00614695
rs143895802	19	45444082	A	G	52.244	0.055	0.008	4.90E-13	0.240	0.251	0.3394
rs144032256	16	57040141	T	C	55.337	-0.057	0.008	1.00E-13	0.027	0.094	0.772499
rs144033177	20	571467	C	A	50.254	-0.056	0.008	1.40E-12	-0.091	0.099	0.3567
rs144133183	11	49188396	C	G	32.935	-0.052	0.009	9.50E-09	-0.113	0.084	0.179
rs144420579	18	47568714	G	T	56.816	-0.043	0.006	4.80E-14	0.068	0.098	0.4859
rs1446585	2	136407479	G	A	59.734	0.017	0.002	1.10E-14	0.063	0.027	0.01704
rs14486613	15	58678179	A	G	36.444	-0.056	0.009	1.60E-09	0.059	0.139	0.6705

0											
rs145328206	19	7995934	C	T	41.889	0.068	0.010	9.70E-11	0.017	0.077	0.8295
rs145943094	3	47881942	T	C	54.143	-0.055	0.007	1.90E-13	0.117	0.046	0.0109701
rs145990683	11	116721465	A	G	32.421	0.064	0.011	1.20E-08	0.275	0.155	0.0762307
rs146203232	6	160543148	T	C	34.188	-0.021	0.004	5.00E-09	-0.031	0.054	0.5606
rs146591430	6	31507160	G	A	54.863	-0.058	0.008	1.30E-13	-0.026	0.077	0.736
rs146696797	6	34262438	C	G	43.141	-0.030	0.005	5.10E-11	-0.009	0.049	0.8521
rs1468642	19	52342703	A	T	74.119	-0.017	0.002	7.40E-18	0.007	0.027	0.778999
rs1469100	5	52721494	C	T	48.074	-0.014	0.002	4.10E-12	0.033	0.026	0.2092
rs146968757	16	56666918	A	G	37.330	0.049	0.008	1.00E-09	-0.008	0.072	0.9082
rs147146149	15	58743495	G	C	41.420	-0.055	0.009	1.20E-10	-0.069	0.121	0.5714
rs1472267	6	166185025	G	A	29.798	-0.011	0.002	4.80E-08	-0.010	0.026	0.7082
rs147294109	14	105172807	T	C	34.008	-0.036	0.006	5.50E-09	0.039	0.066	0.5536
rs147481829	16	56537705	T	C	47.964	-0.067	0.010	4.30E-12	0.076	0.073	0.2974
rs147525635	15	43468698	A	G	48.720	-0.013	0.002	3.00E-12	0.036	0.026	0.165
rs1476161	17	8090908	G	A	47.193	-0.013	0.002	6.40E-12	-0.040	0.028	0.1492
rs148048657	8	19774005	A	G	29.768	-0.035	0.006	4.90E-08	-0.044	0.085	0.6102
rs150300171	7	74102895	C	A	41.565	-0.029	0.005	1.10E-10	0.127	0.088	0.1494

rs150844304	15	43726625	C	A	228.320	-0.091	0.006	1.40E-51	-0.231	0.181	0.2033
rs150861794	13	109003805	T	C	34.612	-0.045	0.008	4.00E-09	-0.345	0.148	0.0199701
rs1518165	18	40702224	T	C	42.313	0.013	0.002	7.80E-11	0.010	0.025	0.692901
rs1534696	7	26397239	A	C	75.954	0.017	0.002	2.90E-18	-0.010	0.026	0.686099
rs1545718	8	9113463	T	G	41.921	-0.013	0.002	9.50E-11	-0.011	0.030	0.699
rs1552781	8	19624132	G	C	221.686	0.034	0.002	3.90E-50	-0.016	0.028	0.5514
rs157935	7	130585553	G	T	31.466	0.012	0.002	2.00E-08	-0.026	0.026	0.3228
rs158482	16	57076115	G	T	237.083	0.113	0.007	1.70E-53	0.063	0.190	0.7415
rs1601935	15	58671765	T	G	2302.767	-0.097	0.002	1.00E-200	0.019	0.026	0.4656
rs1666658	11	121801129	C	T	66.920	-0.016	0.002	2.80E-16	0.019	0.026	0.4643
rs16928809	11	2936952	A	G	63.342	-0.026	0.003	1.70E-15	0.085	0.048	0.0770194
rs16940262	15	58717428	C	T	76.561	0.019	0.002	2.10E-18	-0.004	0.026	0.8646
rs16965150	16	57059484	T	C	89.841	0.054	0.006	2.60E-21	0.069	0.132	0.5993
rs16969990	17	46230891	T	C	44.994	0.025	0.004	2.00E-11	-0.057	0.045	0.2016
rs16975758	17	68419330	A	G	33.815	0.012	0.002	6.10E-09	0.011	0.028	0.6978
rs17041868	2	111894720	C	T	40.108	-0.025	0.004	2.40E-10	0.066	0.062	0.2864
rs17066842	18	58040624	A	G	36.593	0.030	0.005	1.50E-09	-0.070	0.082	0.3911
rs17124112	14	88605509	A	C	34.725	-0.021	0.004	3.80E-09	-0.035	0.053	0.5043
rs17138358	7	17920253	C	G	195.335	-0.027	0.002	2.20E-44	0.012	0.028	0.6615
rs17150845	8	9817353	A	G	44.300	0.017	0.003	2.80E-11	-0.024	0.034	0.4903
rs17185038	1	28219658	G	C	30.876	0.021	0.004	2.80E-08	-0.053	0.064	0.4111
rs17269439	15	59063144	A	C	34.174	-0.030	0.005	5.00E-09	-0.123	0.077	0.1122
rs17309930	11	27748493	A	C	86.363	-0.022	0.002	1.50E-20	0.079	0.030	0.00737802
rs17319046	8	126350794	G	T	41.194	0.023	0.004	1.40E-10	0.004	0.052	0.9451
rs17326656	2	48962291	T	G	100.034	-0.022	0.002	1.50E-23	0.032	0.034	0.3431
rs17406806	12	27064785	C	A	33.834	0.025	0.004	6.00E-09	0.040	0.045	0.3758
rs174566	11	61592362	G	A	793.848	-0.056	0.002	1.20E-174	-0.009	0.026	0.7405
rs17566828	11	111811842	A	G	38.457	0.030	0.005	5.60E-10	-0.069	0.101	0.490601
rs17600636	2	128600609	C	T	42.577	-0.013	0.002	6.80E-11	0.044	0.026	0.087579

rs17608459	1	110435328	A	G	47.753	-0.022	0.003	4.80E-12	-0.033	0.042	0.4268
rs1760940	14	20938251	C	A	30.214	0.012	0.002	3.90E-08	-0.025	0.031	0.4166
rs17641263	1	109666352	T	A	35.629	0.016	0.003	2.40E-09	-0.006	0.036	0.8591
rs17705031	6	121803816	G	A	30.161	-0.013	0.002	4.00E-08	0.028	0.028	0.318
rs1771582	1	161614490	G	T	42.822	0.013	0.002	6.00E-11	-0.027	0.027	0.3153
rs17740942	8	116891360	A	T	42.601	0.020	0.003	6.70E-11	-0.049	0.046	0.2834
rs1800961	20	43042364	T	C	637.026	-0.139	0.006	1.50E-140	0.019	0.061	0.761001
rs1818917	15	23941678	T	C	35.444	0.011	0.002	2.60E-09	-0.032	0.026	0.2084
rs1823291	4	111586963	A	G	29.924	0.011	0.002	4.50E-08	-0.019	0.026	0.455
rs183078	13	49513352	G	A	40.561	0.012	0.002	1.90E-10	-0.033	0.027	0.2249
rs183906992	7	95011948	C	T	38.493	0.029	0.005	5.50E-10	0.015	0.064	0.8118
rs1862205	5	108656635	A	G	33.906	0.011	0.002	5.80E-09	-0.042	0.026	0.1016
rs1869143	15	58792483	G	T	30.375	-0.015	0.003	3.60E-08	0.003	0.038	0.9394
rs187471874	12	125339796	A	C	79.636	0.083	0.009	4.50E-19	-0.278	0.156	0.0758508
rs187544997	8	19890641	G	C	59.666	-0.059	0.008	1.10E-14	0.037	0.261	0.8881
rs1878051	1	235094643	A	T	42.485	0.013	0.002	7.10E-11	-0.039	0.028	0.1609
rs187929675	11	117111368	T	C	322.769	0.151	0.008	3.60E-72	-0.240	0.120	0.0462498
rs1884589	20	21885619	C	A	44.830	-0.013	0.002	2.10E-11	-0.015	0.026	0.5615
rs190712692	19	45425178	A	G	242.535	0.068	0.004	1.10E-54	0.000	0.071	0.9962
rs1955512	14	33175822	A	G	31.169	0.011	0.002	2.40E-08	0.015	0.026	0.569
rs1970811	10	126696496	C	T	36.664	-0.012	0.002	1.40E-09	-0.026	0.026	0.3272
rs2011614	17	28781792	A	G	51.854	-0.014	0.002	6.00E-13	-0.006	0.027	0.8331
rs201441	7	101737327	G	T	32.151	-0.011	0.002	1.40E-08	-0.014	0.025	0.5938
rs204474	19	45487178	T	C	414.089	-0.041	0.002	4.70E-92	0.013	0.026	0.615601
rs2052963	8	10675623	T	G	38.525	-0.014	0.002	5.40E-10	0.053	0.030	0.0765191
rs2068888	10	94839642	A	G	100.296	0.019	0.002	1.30E-23	-0.002	0.025	0.9318

rs2071379	17	26695832	G	A	48.522	-0.014	0.002	3.30E-12	0.018	0.025	0.4857
rs2098918	4	37151533	T	C	38.381	0.012	0.002	5.80E-10	0.015	0.026	0.5692
rs2115107	19	7968168	A	G	53.681	-0.014	0.002	2.40E-13	0.031	0.026	0.2323
rs2137537	12	71113087	C	T	32.474	-0.011	0.002	1.20E-08	-0.042	0.026	0.114
rs2148252	6	43794144	A	G	34.837	-0.012	0.002	3.60E-09	-0.007	0.026	0.782099
rs2156585	11	109960423	G	A	56.097	-0.016	0.002	6.90E-14	0.046	0.026	0.0733398
rs2159607	3	52501451	T	G	96.933	-0.024	0.002	7.20E-23	0.069	0.043	0.1104
rs2165557	8	19835050	T	A	541.681	0.057	0.002	8.09E-120	-0.041	0.034	0.227
rs2175766	4	76572191	C	A	31.791	0.011	0.002	1.70E-08	0.031	0.025	0.2238
rs2218793	11	10380828	A	C	50.182	-0.015	0.002	1.40E-12	0.011	0.028	0.7036
rs223487	4	103683546	C	A	62.449	0.019	0.002	2.70E-15	-0.024	0.029	0.4101
rs2237035	4	55526251	T	G	50.509	0.014	0.002	1.20E-12	0.019	0.027	0.4693
rs2245477	15	61948435	A	C	33.466	-0.011	0.002	7.30E-09	-0.006	0.026	0.813
rs2247355	8	103876780	T	C	70.290	0.021	0.002	5.10E-17	0.002	0.029	0.9522
rs2256609	22	21925017	G	A	181.590	-0.033	0.002	2.20E-41	0.037	0.028	0.1848
rs2263329	6	34595543	C	T	188.339	-0.027	0.002	7.30E-43	0.030	0.027	0.2533
rs2268840	3	185931174	C	T	58.330	0.017	0.002	2.20E-14	-0.037	0.030	0.2139
rs2281279	20	46290250	C	T	47.378	0.014	0.002	5.90E-12	0.049	0.032	0.1202
rs2281525	1	93595306	C	G	37.468	-0.013	0.002	9.30E-10	0.036	0.029	0.2086
rs2289863	19	4028783	T	C	65.299	-0.018	0.002	6.40E-16	0.040	0.028	0.1548
rs2292749	17	40818584	T	C	61.014	-0.016	0.002	5.70E-15	-0.001	0.028	0.9717
rs2297402	9	107579880	T	C	110.577	-0.068	0.007	7.30E-26	0.034	0.125	0.7855
rs2298214	1	935222	A	C	40.879	-0.012	0.002	1.60E-10	-0.021	0.026	0.4172
rs2298632	1	23710475	T	C	55.435	0.014	0.002	9.70E-14	0.002	0.026	0.9253
rs2302367	12	6861043	C	T	30.549	-0.011	0.002	3.30E-08	-0.021	0.026	0.4166
rs2306828	17	43213772	C	A	32.795	0.015	0.003	1.00E-08	-0.069	0.035	0.0503605
rs2307111	5	75003678	C	T	94.786	0.019	0.002	2.10E-22	0.032	0.026	0.2088
rs2339234	5	170612546	A	G	33.460	-0.012	0.002	7.30E-09	-0.002	0.027	0.9401
rs235314	21	46271452	T	C	85.400	-0.018	0.002	2.40E-20	0.002	0.025	0.9521
rs2362541	2	30478453	G	T	32.598	-0.011	0.002	1.10E-08	-0.022	0.026	0.3965
rs2364723	2	178126546	C	G	34.936	0.012	0.002	3.40E-09	0.005	0.027	0.8508

rs236987	4	88009779	T	C	103.706	0.020	0.002	2.30E-24	0.005	0.025	0.8488
rs2370998	2	211430333	A	G	48.959	0.013	0.002	2.60E-12	-0.007	0.026	0.7913
rs2414178	15	53001538	C	T	31.121	0.013	0.002	2.40E-08	0.068	0.028	0.0167201
rs2417125	9	131562232	G	A	38.614	-0.013	0.002	5.20E-10	-0.010	0.031	0.7327
rs2435307	18	21127910	T	C	72.677	0.016	0.002	1.50E-17	0.018	0.025	0.4828
rs2447589	11	67407782	G	A	36.468	0.014	0.002	1.60E-09	-0.080	0.035	0.0233002
rs248653	5	130656028	A	T	39.509	-0.032	0.005	3.30E-10	0.007	0.051	0.8834
rs2498786	14	105262368	G	C	167.507	-0.025	0.002	2.60E-38	0.023	0.026	0.383
rs2516331	1	212416989	A	C	41.596	0.013	0.002	1.10E-10	-0.014	0.028	0.635501
rs2534596	7	38277792	G	A	33.264	0.011	0.002	8.00E-09	-0.003	0.027	0.9092
rs254024	5	103944020	T	G	29.771	-0.010	0.002	4.90E-08	0.032	0.026	0.2109
rs254562	5	134441457	G	A	34.919	-0.011	0.002	3.40E-09	-0.001	0.026	0.9557
rs2581822	3	53021749	A	G	32.113	-0.012	0.002	1.50E-08	0.102	0.027	0.000154501
rs2586116	17	38505824	G	C	56.038	-0.016	0.002	7.10E-14	0.050	0.030	0.090261
rs260970	1	39551242	A	G	33.427	-0.014	0.002	7.40E-09	-0.032	0.031	0.308
rs2642438	1	220970028	G	A	177.039	0.028	0.002	2.10E-40	-0.041	0.028	0.1395
rs2645979	12	84017043	A	G	33.110	0.011	0.002	8.70E-09	0.027	0.025	0.287
rs2663924	15	81392903	G	T	30.571	0.012	0.002	3.20E-08	-0.042	0.028	0.1384
rs267738	1	150940625	G	T	87.184	0.021	0.002	9.90E-21	0.050	0.032	0.1219
rs268	8	19813529	G	A	1364.644	-0.262	0.007	1.00E-200	-0.049	0.085	0.566601
rs2704190	15	58371251	C	T	36.118	-0.018	0.003	1.90E-09	0.006	0.038	0.871
rs2723065	2	65279414	G	A	58.133	0.015	0.002	2.40E-14	-0.038	0.027	0.1523
rs2740488	9	107661742	C	A	1008.475	-0.069	0.002	1.00E-200	-0.007	0.032	0.8352
rs2750411	6	139226630	G	T	32.243	-0.011	0.002	1.40E-08	-0.035	0.025	0.1704
rs2777803	9	107571208	A	G	250.951	0.050	0.003	1.60E-56	0.017	0.043	0.6989
rs2781668	6	131897278	T	C	42.178	-0.017	0.003	8.30E-11	0.011	0.030	0.7258
rs2792751	10	113940329	C	T	286.275	-0.036	0.002	3.20E-64	0.032	0.027	0.2337
rs2800710	6	127452581	C	T	113.330	-0.020	0.002	1.80E-26	0.019	0.025	0.4584
rs2804894	10	33647091	A	G	62.786	0.017	0.002	2.30E-15	0.001	0.031	0.9639
rs28362901	15	74712937	A	C	50.461	-0.024	0.003	1.20E-12	0.087	0.040	0.02848
rs28510484	15	31637569	C	G	37.005	-0.015	0.003	1.20E-09	0.028	0.033	0.4034

rs28546518	8	19934043	G	A	114.064	0.031	0.003	1.30E-26	0.039	0.032	0.2281
rs28690720	15	58573833	G	T	676.122	-0.072	0.003	4.70E-149	0.103	0.041	0.0113
rs289754	16	57065556	T	C	333.859	-0.038	0.002	1.40E-74	0.029	0.028	0.307
rs2910949	5	39522481	G	T	44.936	0.013	0.002	2.00E-11	-0.024	0.030	0.4246
rs2914003	19	48093841	G	T	33.735	0.017	0.003	6.30E-09	0.042	0.042	0.3174
rs2925979	16	81534790	C	T	328.927	0.037	0.002	1.60E-73	0.006	0.027	0.8261
rs2943645	2	227099180	T	C	477.985	-0.043	0.002	5.90E-106	0.052	0.026	0.04917
rs2963468	5	158003020	G	A	75.544	-0.020	0.002	3.60E-18	0.016	0.037	0.6721
rs2964012	5	153201369	G	A	47.286	0.014	0.002	6.10E-12	-0.020	0.028	0.4645
rs2972166	3	12316339	A	G	69.152	0.018	0.002	9.10E-17	-0.045	0.027	0.0917403
rs2972559	19	45357291	G	C	110.968	-0.023	0.002	6.00E-26	-0.055	0.030	0.0648605
rs2972561	19	45340021	T	C	36.320	-0.046	0.008	1.70E-09	-0.154	0.191	0.4222
rs3001283	1	182263489	T	C	34.590	0.027	0.005	4.10E-09	-0.002	0.083	0.9771
rs308	8	19817476	G	T	357.274	0.127	0.007	1.10E-79	0.082	0.074	0.271
rs3135507	11	116661488	T	C	39.807	-0.039	0.006	2.80E-10	-0.085	0.061	0.1631
rs3184504	12	111884608	C	T	194.173	0.027	0.002	3.90E-44	-0.078	0.026	0.00228102
rs32578	5	149211868	A	G	41.624	0.013	0.002	1.10E-10	-0.017	0.026	0.5226
rs3289	8	19823192	C	T	825.878	-0.167	0.006	1.30E-181	-0.092	0.092	0.3148
rs333947	1	110470764	A	G	140.046	-0.032	0.003	2.60E-32	-0.021	0.034	0.5362
rs33918808	9	107579632	G	C	81.653	0.050	0.006	1.60E-19	-0.069	0.084	0.4125
rs34046876	11	539058	T	C	32.902	0.016	0.003	9.70E-09	0.004	0.044	0.9344
rs34106738	3	150181750	A	G	39.773	0.015	0.002	2.90E-10	0.049	0.037	0.1819
rs34149579	12	123345509	T	G	112.741	-0.046	0.004	2.50E-26	-0.138	0.055	0.0111599
rs343	8	19810787	A	C	1491.286	0.134	0.003	1.00E-200	-0.012	0.052	0.8243
rs34559316	19	38863464	A	C	31.852	-0.014	0.002	1.70E-08	0.020	0.030	0.5133
rs34830321	16	58616997	T	C	33.447	-0.048	0.008	7.30E-09	-0.010	0.228	0.9645
rs35137994	19	8429066	T	C	125.804	0.046	0.004	3.40E-29	-0.192	0.069	0.00569994
rs35169799	11	64031241	T	C	128.203	-0.044	0.004	1.00E-29	0.059	0.043	0.1752
rs35257100	7	26135404	T	C	33.297	0.013	0.002	7.90E-09	0.049	0.031	0.1198
rs35473170	4	69361441	A	G	61.409	0.016	0.002	4.60E-15	0.016	0.025	0.5239
rs35493868	7	73039406	G	C	245.118	0.037	0.002	3.00E-55	-0.074	0.033	0.02426

rs35892692	12	122317659	A	C	43.055	0.013	0.002	5.30E-11	0.027	0.027	0.3249
rs35897671	5	127349745	T	C	55.752	0.015	0.002	8.20E-14	0.034	0.029	0.2367
rs36092527	19	56181408	C	T	32.110	-0.017	0.003	1.50E-08	0.044	0.040	0.2784
rs367070	19	54800500	G	A	333.949	0.042	0.002	1.30E-74	0.048	0.028	0.0806901
rs367677	14	69273905	G	A	54.998	0.017	0.002	1.20E-13	0.027	0.028	0.3396
rs3732356	3	119529113	T	G	59.850	-0.030	0.004	1.00E-14	-0.031	0.050	0.5376
rs3733890	5	78421959	A	G	43.609	-0.014	0.002	4.00E-11	0.006	0.030	0.8321
rs3736802	20	33604042	C	T	77.503	0.017	0.002	1.30E-18	-0.085	0.026	0.000975192
rs3740688	11	47380340	T	G	162.374	0.024	0.002	3.40E-37	-0.007	0.029	0.8111
rs3746915	21	43718792	G	A	31.428	0.011	0.002	2.10E-08	-0.032	0.026	0.2247
rs3747973	1	205677148	G	A	53.433	0.014	0.002	2.70E-13	0.001	0.026	0.9566
rs375054	16	56578386	C	T	220.579	-0.053	0.004	6.80E-50	-0.017	0.048	0.7279
rs3759335	12	6493094	T	C	31.103	0.015	0.003	2.40E-08	-0.078	0.039	0.0430299
rs3768321	1	40035928	T	G	356.697	-0.045	0.002	1.50E-79	-0.026	0.034	0.4496
rs3794752	17	53382829	C	T	36.000	0.013	0.002	2.00E-09	-0.020	0.028	0.4842
rs3806410	1	156337134	T	C	29.852	0.013	0.002	4.70E-08	0.013	0.030	0.6748
rs3811476	1	230469692	A	G	30.353	0.018	0.003	3.60E-08	0.042	0.046	0.3554
rs3814883	16	29994922	T	C	65.458	-0.015	0.002	5.90E-16	0.062	0.026	0.0149101
rs38166	7	15889360	C	T	32.042	0.013	0.002	1.50E-08	-0.027	0.030	0.3717
rs3825036	11	61516476	A	G	40.511	0.018	0.003	2.00E-10	0.029	0.034	0.3828
rs3825669	14	89804276	G	A	34.945	0.014	0.002	3.40E-09	0.082	0.036	0.02216
rs3859113	16	56699967	T	C	289.167	-0.065	0.004	7.60E-65	0.049	0.059	0.4039
rs3936511	5	55860781	G	A	158.952	-0.031	0.002	1.90E-36	0.057	0.037	0.125
rs40270	5	55804552	C	A	141.712	-0.027	0.002	1.10E-32	-0.005	0.028	0.852
rs407238	6	29806901	G	C	66.934	0.016	0.002	2.80E-16	-0.002	0.026	0.9412
rs41266485	3	127390525	T	C	44.306	-0.016	0.002	2.80E-11	-0.040	0.029	0.1597
rs41292412	18	56118358	T	C	55.547	-0.066	0.009	9.10E-14	-0.284	0.134	0.0336798
rs4149265	9	107672498	A	G	65.874	-0.018	0.002	4.80E-16	-0.031	0.028	0.2725
rs4149307	9	107589744	T	C	584.955	0.064	0.003	3.10E-129	0.032	0.040	0.4297
rs4239651	20	46341313	C	T	121.113	0.026	0.002	3.60E-28	0.026	0.031	0.3961
rs4245443	11	76183924	G	A	31.291	-0.011	0.002	2.20E-08	-0.034	0.026	0.1928

rs429358	19	45411941	C	T	822.047	-0.076	0.003	8.71E-181	-0.066	0.033	0.0480496
rs4391855	11	116350751	A	G	45.802	-0.033	0.005	1.30E-11	0.000	0.077	0.996
rs4397962	13	28687084	C	T	36.341	-0.014	0.002	1.70E-09	0.034	0.028	0.2273
rs4410790	7	17284577	C	T	31.135	-0.011	0.002	2.40E-08	-0.055	0.027	0.0390904
rs4450131	10	126383363	C	T	33.517	0.011	0.002	7.10E-09	0.039	0.025	0.1234
rs445841	5	122331222	T	G	30.178	-0.011	0.002	3.90E-08	-0.019	0.029	0.5021
rs4500815	18	46332908	G	A	30.130	-0.012	0.002	4.00E-08	0.009	0.030	0.7612
rs454968	5	112188456	C	T	30.598	0.011	0.002	3.20E-08	0.030	0.026	0.2522
rs4550673	2	112941372	G	A	31.348	-0.019	0.003	2.20E-08	-0.006	0.064	0.9252
rs4599108	2	85549547	T	C	50.429	0.014	0.002	1.20E-12	0.027	0.026	0.2873
rs4622308	12	56470625	T	C	33.238	-0.011	0.002	8.20E-09	0.004	0.026	0.8616
rs4643686	3	153177649	T	C	33.601	-0.013	0.002	6.80E-09	0.001	0.031	0.982
rs4650994	1	178515312	A	G	90.252	-0.018	0.002	2.10E-21	0.040	0.025	0.1121
rs4654395	1	29567412	T	C	39.772	-0.012	0.002	2.90E-10	-0.005	0.026	0.8347
rs4656984	1	161078909	C	T	31.057	0.011	0.002	2.50E-08	0.006	0.026	0.8284
rs4691379	4	157706904	T	C	33.758	0.012	0.002	6.20E-09	0.060	0.029	0.0421697
rs4731701	7	130430930	T	C	241.287	0.030	0.002	2.10E-54	-0.006	0.025	0.807
rs4742937	9	107780966	C	T	48.509	-0.015	0.002	3.30E-12	-0.022	0.032	0.4899
rs4755720	11	43628749	T	C	32.660	0.011	0.002	1.10E-08	-0.049	0.026	0.0611406
rs4760	19	44153100	G	A	53.302	-0.019	0.003	2.90E-13	0.029	0.034	0.3978
rs4765636	12	125359899	G	C	48.647	0.033	0.005	3.10E-12	-0.092	0.112	0.4097
rs4775038	15	58658794	T	C	285.496	-0.053	0.003	4.80E-64	0.056	0.049	0.2542
rs4775075	15	58837764	G	A	31.147	0.019	0.003	2.40E-08	0.004	0.047	0.9291
rs4783961	16	56994894	A	G	2308.131	0.091	0.002	1.00E-200	0.005	0.025	0.8536
rs4784705	16	56696327	G	T	78.766	-0.066	0.007	7.00E-19	-0.054	0.097	0.578801
rs4784779	16	57183725	C	T	61.918	0.024	0.003	3.60E-15	0.034	0.045	0.4476
rs4802269	19	46167469	G	A	34.051	0.012	0.002	5.40E-09	-0.050	0.027	0.0616098
rs4804312	19	8640107	C	T	40.272	0.012	0.002	2.20E-10	0.033	0.025	0.1918
rs4841584	8	11601510	C	A	33.931	0.022	0.004	5.70E-09	0.010	0.048	0.8395
rs4855582	3	108867705	T	C	33.687	0.011	0.002	6.50E-09	0.001	0.025	0.9782
rs4865796	5	53272664	A	G	39.794	-0.013	0.002	2.80E-10	0.015	0.027	0.5815

rs4871603	8	126480367	T	C	326.626	0.036	0.002	5.20E-73	0.002	0.027	0.9436
rs4875043	8	144496772	C	A	41.357	-0.015	0.002	1.30E-10	0.041	0.030	0.1753
rs4919044	10	94771895	C	T	40.163	0.022	0.003	2.30E-10	-0.103	0.046	0.0236102
rs4930352	11	66066993	T	G	69.587	0.016	0.002	7.30E-17	-0.016	0.025	0.5426
rs4939921	18	47462328	C	T	31.263	0.018	0.003	2.30E-08	-0.041	0.036	0.2656
rs4947121	6	111834954	C	T	38.567	-0.014	0.002	5.30E-10	0.040	0.034	0.2476
rs4969141	17	76391653	T	C	240.392	0.030	0.002	3.20E-54	0.051	0.026	0.0515798
rs4976033	5	67714246	G	A	37.165	-0.012	0.002	1.10E-09	0.005	0.026	0.8528
rs4986970	16	67976320	T	A	213.280	-0.076	0.005	2.60E-48	-0.085	0.075	0.2581
rs500647	1	71413498	A	G	29.818	0.011	0.002	4.70E-08	0.036	0.026	0.1667
rs532436	9	136149830	A	G	89.054	0.023	0.002	3.80E-21	-0.045	0.031	0.1505
rs536003	1	40154898	A	G	31.652	0.013	0.002	1.80E-08	0.049	0.032	0.1323
rs546436456	4	27979911	G	T	30.356	-0.032	0.006	3.60E-08	0.169	0.196	0.3895
rs549058	13	51201045	T	G	33.938	0.017	0.003	5.70E-09	-0.028	0.042	0.513
rs55700285	7	150210148	T	G	67.065	-0.019	0.002	2.60E-16	0.022	0.027	0.4171
rs55781197	16	68032598	G	A	396.027	0.059	0.003	4.00E-88	-0.019	0.035	0.5869
rs557933	1	234853268	C	A	63.871	0.015	0.002	1.30E-15	-0.038	0.026	0.1402
rs55935382	7	50289669	A	C	74.902	0.018	0.002	4.90E-18	-0.011	0.027	0.695401
rs559355	11	75451281	T	A	178.772	-0.035	0.003	9.00E-41	0.013	0.030	0.676799
rs55960209	16	56106553	G	T	46.644	0.043	0.006	8.50E-12	-0.176	0.169	0.298
rs56090699	8	19998949	C	T	79.462	0.019	0.002	4.90E-19	0.011	0.028	0.690799
rs56131490	2	135263081	A	G	31.677	0.015	0.003	1.80E-08	0.009	0.047	0.844
rs561770008	19	33864417	T	G	30.990	0.037	0.007	2.60E-08	0.171	0.119	0.1532
rs56221808	18	47089971	G	A	58.885	0.046	0.006	1.70E-14	0.058	0.108	0.5883
rs567056	11	30435051	T	G	33.744	-0.011	0.002	6.30E-09	0.085	0.026	0.000913293
rs571848809	6	161008646	A	G	335.854	-0.057	0.003	5.10E-75	0.015	0.042	0.728501
rs5758910	22	43145853	C	G	31.871	0.012	0.002	1.60E-08	0.062	0.028	0.02683
rs577525	10	99769388	C	T	53.969	-0.014	0.002	2.00E-13	0.056	0.025	0.0273401

rs58298943	12	57391292	T	C	34.729	0.020	0.003	3.80E-09	-0.030	0.042	0.4714
rs58473820	11	122514403	T	C	169.959	0.026	0.002	7.60E-39	-0.036	0.026	0.1742
rs58770498	6	32631029	C	T	52.797	-0.037	0.005	3.70E-13	-0.107	0.068	0.1161
rs5891	12	125299542	T	C	52.992	0.066	0.009	3.30E-13	0.380	0.322	0.2378
rs59104589	2	242237902	T	C	57.418	0.015	0.002	3.50E-14	-0.020	0.026	0.4463
rs595767	17	46957987	G	A	56.666	-0.014	0.002	5.20E-14	-0.035	0.025	0.1627
rs59737437	19	2671100	T	C	29.885	0.012	0.002	4.60E-08	-0.038	0.027	0.1636
rs59781045	11	117095283	T	C	382.033	0.074	0.004	4.50E-85	0.097	0.046	0.0349599
rs6017734	20	44724837	C	T	52.136	0.014	0.002	5.20E-13	-0.009	0.025	0.7341
rs6021914	20	51031169	C	T	37.312	0.012	0.002	1.00E-09	-0.016	0.028	0.5643
rs60305172	9	107248779	C	T	34.449	0.048	0.008	4.40E-09	-0.109	0.093	0.2391
rs60536263	9	15280187	C	T	32.389	0.017	0.003	1.30E-08	0.005	0.039	0.8936
rs60570301	19	18597196	A	G	29.928	0.016	0.003	4.50E-08	0.051	0.035	0.1425
rs6059958	20	30143278	T	C	32.891	0.015	0.003	9.70E-09	-0.021	0.031	0.499401
rs6063153	20	46454945	T	C	35.142	-0.012	0.002	3.10E-09	-0.021	0.027	0.44
rs6066148	20	45602037	C	G	37.249	0.013	0.002	1.00E-09	-0.048	0.032	0.1291
rs607335	6	153465230	A	C	70.696	0.016	0.002	4.20E-17	0.020	0.025	0.4257
rs6073958	20	44551855	C	T	648.803	-0.061	0.002	4.10E-143	-0.002	0.033	0.9622
rs6123685	20	55836040	A	G	53.179	0.016	0.002	3.00E-13	0.017	0.029	0.5574
rs61352607	12	57839173	T	G	189.376	0.031	0.002	4.40E-43	-0.099	0.030	0.000871004
rs61596977	8	95997165	T	C	35.136	-0.016	0.003	3.10E-09	-0.015	0.035	0.6707
rs61766062	18	47093444	T	C	111.945	-0.030	0.003	3.70E-26	-0.034	0.047	0.477
rs61781290	1	40393160	G	A	55.880	-0.016	0.002	7.70E-14	-0.035	0.029	0.2202
rs61805075	1	182136401	A	G	161.246	-0.026	0.002	6.00E-37	0.046	0.028	0.0997401
rs61884030	11	14444543	C	T	41.349	0.018	0.003	1.30E-10	0.038	0.029	0.1891
rs61926301	12	51157863	T	G	39.796	0.012	0.002	2.80E-10	0.001	0.027	0.9639
rs61941677	12	125325010	G	C	334.144	-0.053	0.003	1.20E-74	0.005	0.036	0.8914
rs61993685	14	100765823	C	T	30.771	0.020	0.004	2.90E-08	-0.054	0.047	0.2538
rs62005949	15	67266003	A	G	29.833	0.011	0.002	4.70E-08	0.022	0.028	0.4303
rs62037364	16	28868695	A	G	30.711	-0.011	0.002	3.00E-08	0.018	0.026	0.4926
rs62102718	19	33891013	T	A	125.815	-0.024	0.002	3.40E-29	0.068	0.028	0.0143001

rs62114548	2	274672	G	C	51.826	0.014	0.002	6.10E-13	-0.018	0.026	0.4868
rs62117206	19	45255679	C	G	102.179	0.038	0.004	5.10E-24	-0.061	0.052	0.2444
rs62271373	3	150066540	A	T	99.021	-0.041	0.004	2.50E-23	-0.011	0.062	0.8569
rs62428831	6	143247161	C	T	41.804	0.018	0.003	1.00E-10	-0.054	0.032	0.0944996
rs62486442	8	12623463	A	G	48.816	-0.014	0.002	2.80E-12	0.010	0.026	0.7118
rs62565259	9	102162570	T	C	38.433	0.016	0.003	5.70E-10	0.029	0.037	0.437
rs635769	6	139831981	C	T	100.110	0.020	0.002	1.40E-23	0.020	0.026	0.4275
rs642895	12	123158291	T	C	47.059	0.020	0.003	6.90E-12	-0.021	0.038	0.568
rs6469605	8	116601894	T	C	271.330	0.032	0.002	5.80E-61	-0.078	0.028	0.00464997
rs6479631	9	136882821	T	C	48.828	-0.015	0.002	2.80E-12	0.007	0.026	0.7758
rs6503468	17	41772813	C	T	40.767	-0.013	0.002	1.70E-10	0.017	0.026	0.5085
rs6541284	1	230254434	G	T	51.292	0.022	0.003	8.00E-13	0.005	0.038	0.89
rs6664374	1	66073952	T	C	49.155	0.014	0.002	2.40E-12	0.030	0.025	0.2442
rs66763009	7	36193142	G	T	69.000	-0.016	0.002	9.80E-17	0.028	0.026	0.2695
rs66781087	18	47212159	A	G	183.006	0.026	0.002	1.10E-41	-0.010	0.025	0.693
rs67344323	8	64653461	C	T	44.520	-0.015	0.002	2.50E-11	-0.054	0.032	0.0892401
rs6738438	2	230020220	T	C	32.725	-0.011	0.002	1.10E-08	0.006	0.027	0.8253
rs674424	11	119030752	C	T	35.891	-0.013	0.002	2.10E-09	0.026	0.031	0.3976
rs67516712	12	124201524	A	G	52.346	-0.014	0.002	4.70E-13	0.035	0.027	0.205
rs676210	2	21231524	A	G	634.819	0.059	0.002	4.50E-140	0.019	0.029	0.519
rs6765484	3	50041313	T	C	139.225	0.023	0.002	3.90E-32	-0.002	0.025	0.924
rs680321	18	29797958	C	T	32.058	0.011	0.002	1.50E-08	0.010	0.025	0.683701
rs6806529	3	123049938	C	A	33.730	0.011	0.002	6.30E-09	0.010	0.026	0.692901
rs68160490	1	228078132	A	G	36.400	0.012	0.002	1.60E-09	0.010	0.026	0.6848
rs6824451	4	89723065	A	G	109.941	-0.020	0.002	1.00E-25	-0.033	0.025	0.1915
rs686030	9	15304782	A	C	330.775	0.050	0.003	6.50E-74	-0.030	0.039	0.4436
rs6874833	5	75696662	T	C	32.835	0.011	0.002	1.00E-08	0.006	0.026	0.8244
rs6893139	5	124066859	A	G	31.253	-0.011	0.002	2.30E-08	-0.010	0.029	0.718199
rs690	15	58834741	T	G	59.386	-0.016	0.002	1.30E-14	0.031	0.026	0.2245
rs6902116	6	31300286	G	A	175.857	-0.027	0.002	3.90E-40	-0.008	0.029	0.7896
rs6924387	6	137082948	G	A	49.575	-0.014	0.002	1.90E-12	0.039	0.026	0.1305

rs6934962	6	116322349	T	C	69.578	0.016	0.002	7.30E-17	0.032	0.025	0.2047
rs7036107	9	92177897	G	A	39.733	-0.012	0.002	2.90E-10	0.025	0.025	0.3214
rs7039168	9	13719203	G	A	31.913	0.012	0.002	1.60E-08	0.010	0.028	0.7302
rs703966	10	80954251	A	G	65.820	0.016	0.002	4.90E-16	-0.002	0.026	0.9296
rs7114162	11	45916781	G	A	30.094	-0.012	0.002	4.10E-08	-0.028	0.027	0.3006
rs7133378	12	124409502	A	G	338.270	0.038	0.002	1.50E-75	-0.032	0.028	0.2562
rs7134150	12	20591332	A	G	57.226	-0.029	0.004	3.90E-14	0.132	0.076	0.0797701
rs71478653	15	58500953	G	C	102.001	0.059	0.006	5.60E-24	-0.058	0.071	0.4186
rs7158166	14	103241799	C	T	52.180	0.014	0.002	5.10E-13	-0.047	0.026	0.0703996
rs7170463	15	41888918	G	A	84.348	0.019	0.002	4.20E-20	-0.010	0.026	0.7188
rs7177692	15	63379733	T	C	84.961	0.018	0.002	3.00E-20	0.040	0.025	0.1122
rs7218647	17	76769605	A	G	32.147	0.011	0.002	1.40E-08	-0.007	0.026	0.792301
rs72555385	7	73123473	G	A	37.181	-0.027	0.004	1.10E-09	0.115	0.086	0.185
rs72647336	8	126445055	A	G	98.946	-0.044	0.004	2.60E-23	-0.042	0.066	0.5272
rs72654647	1	25022314	A	G	39.136	-0.014	0.002	4.00E-10	0.010	0.028	0.7284
rs72729582	14	69149372	G	A	44.946	0.025	0.004	2.00E-11	-0.022	0.059	0.704601
rs72801474	5	132444128	A	G	41.755	0.021	0.003	1.00E-10	0.077	0.055	0.1614
rs72805692	10	71099109	G	A	37.308	-0.018	0.003	1.00E-09	0.032	0.052	0.5302
rs7281183	21	16411667	A	G	33.747	-0.013	0.002	6.30E-09	0.005	0.028	0.8539
rs72823014	10	115786236	A	G	106.683	0.030	0.003	5.20E-25	-0.011	0.043	0.7975
rs72921699	18	46662805	C	T	34.110	-0.028	0.005	5.20E-09	0.031	0.070	0.655
rs72926946	2	203477868	A	C	98.586	-0.021	0.002	3.10E-23	0.005	0.028	0.8516
rs7298751	12	125380232	A	G	240.869	-0.045	0.003	2.50E-54	0.044	0.035	0.2121
rs7308864	12	109871179	G	A	155.238	0.024	0.002	1.20E-35	0.034	0.025	0.1735
rs7312955	12	123751726	C	A	137.170	-0.037	0.003	1.10E-31	-0.034	0.040	0.3932
rs73151974	7	134669523	T	C	35.072	-0.016	0.003	3.20E-09	0.066	0.037	0.07305
rs73243877	4	26047616	G	A	99.048	-0.025	0.003	2.50E-23	-0.015	0.036	0.6762
rs73546877	16	57089786	T	C	34.272	-0.027	0.005	4.80E-09	-0.029	0.091	0.749
rs73667430	8	19644400	T	C	127.912	-0.085	0.008	1.20E-29	-0.124	0.163	0.4465
rs737338	19	11347657	T	C	296.142	-0.089	0.005	2.30E-66	0.009	0.051	0.8676
rs738409	22	44324727	G	C	38.668	-0.014	0.002	5.00E-10	-0.159	0.030	1.58E-07

rs7387969	8	144270473	G	A	32.142	0.011	0.002	1.40E-08	0.024	0.029	0.404
rs74179970	19	11287001	A	G	36.537	-0.021	0.003	1.50E-09	-0.016	0.057	0.775001
rs74328314	1	61670759	G	A	37.047	0.023	0.004	1.20E-09	0.074	0.049	0.1336
rs74486301	16	57125350	A	G	116.129	0.086	0.008	4.50E-27	-0.269	0.211	0.2021
rs74500135	9	106603816	C	T	44.732	0.067	0.010	2.30E-11	-0.327	0.244	0.1789
rs74518630	16	56796333	G	A	76.336	-0.065	0.007	2.40E-18	-0.153	0.078	0.0490196
rs74771917	11	61627960	T	C	62.016	-0.051	0.007	3.40E-15	-0.046	0.061	0.4477
rs7477945	10	33962855	G	A	48.484	-0.016	0.002	3.30E-12	-0.039	0.029	0.1835
rs74819531	7	150610935	C	G	50.350	0.015	0.002	1.30E-12	0.069	0.029	0.01859
rs75054794	1	230823363	G	A	35.030	-0.026	0.004	3.20E-09	-0.005	0.046	0.9067
rs75104038	6	34190104	A	G	114.711	-0.043	0.004	9.10E-27	0.161	0.071	0.0229103
rs7514499	1	205080963	C	T	29.887	-0.013	0.002	4.60E-08	0.019	0.032	0.5497
rs75152587	16	56579961	T	G	123.432	-0.094	0.008	1.10E-28	0.012	0.118	0.9211
rs75246752	1	145630111	C	G	34.055	0.049	0.008	5.40E-09	-0.466	0.215	0.0296702
rs75257814	16	57118381	C	T	191.612	-0.056	0.004	1.40E-43	0.040	0.073	0.5802
rs75479205	6	7255610	G	A	32.539	0.014	0.002	1.20E-08	-0.070	0.030	0.0201999
rs7583067	2	100796850	T	C	42.628	0.015	0.002	6.60E-11	-0.059	0.030	0.0443803
rs76213248	19	11269893	T	C	46.796	0.013	0.002	7.90E-12	-0.015	0.026	0.5563
rs7622114	3	36960660	A	C	35.561	0.012	0.002	2.50E-09	0.045	0.025	0.0745796
rs76246107	19	50121274	A	G	33.514	-0.021	0.004	7.10E-09	-0.015	0.051	0.7753
rs7627555	3	160034574	G	A	35.804	-0.011	0.002	2.20E-09	-0.020	0.025	0.4323
rs76428106	13	28604007	C	T	50.428	-0.062	0.009	1.20E-12	0.054	0.119	0.6496
rs7650845	3	114484372	T	C	33.371	-0.014	0.002	7.60E-09	0.010	0.039	0.793201
rs76530346	9	94010298	A	G	34.598	-0.019	0.003	4.10E-09	0.033	0.051	0.5156
rs7658082	4	91245506	C	T	34.678	-0.011	0.002	3.90E-09	0.020	0.025	0.4384
rs76602912	20	57459868	C	T	48.115	-0.043	0.006	4.00E-12	0.158	0.086	0.0649397
rs76836072	11	116653762	T	C	325.413	0.075	0.004	9.60E-73	0.087	0.054	0.1078
rs76936604	11	116834091	C	A	30.317	-0.064	0.012	3.70E-08	-0.219	0.192	0.2553
rs76970180	15	58554914	T	G	63.832	0.051	0.006	1.40E-15	-0.094	0.134	0.48
rs77129696	16	56457582	T	C	65.386	-0.034	0.004	6.20E-16	0.030	0.050	0.5454
rs771481	1	93846653	A	T	137.560	0.029	0.002	9.10E-32	-0.018	0.033	0.5918

rs7725218	5	1282414	A	G	36.965	-0.012	0.002	1.20E-09	0.018	0.026	0.4948
rs77271869	1	221097630	C	T	36.675	-0.026	0.004	1.40E-09	-0.004	0.049	0.9325
rs7750688	6	41991740	T	C	93.135	0.021	0.002	4.90E-22	0.006	0.029	0.8234
rs7757193	6	109510972	A	G	92.316	-0.019	0.002	7.40E-22	0.025	0.027	0.3471
rs77605964	7	106962948	A	G	55.942	0.017	0.002	7.50E-14	-0.027	0.033	0.4141
rs77675968	8	19959983	C	T	40.386	-0.034	0.005	2.10E-10	0.073	0.060	0.2182
rs77767539	20	44801556	A	G	30.368	0.045	0.008	3.60E-08	-0.041	0.170	0.8082
rs77916918	11	56628086	A	G	37.813	0.032	0.005	7.80E-10	0.090	0.077	0.2464
rs7794796	7	150540196	T	C	68.613	-0.017	0.002	1.20E-16	0.023	0.026	0.3926
rs77960347	18	47109955	G	A	1228.377	0.291	0.008	1.00E-200	-0.067	0.151	0.656299
rs78025076	4	110569620	T	C	53.145	-0.049	0.007	3.10E-13	-0.219	0.138	0.1112
rs78058190	2	219699999	A	G	255.344	-0.078	0.005	1.80E-57	0.082	0.047	0.0766408
rs7817574	8	144302570	C	T	185.210	0.033	0.002	3.50E-42	0.025	0.029	0.3921
rs7826177	8	34406540	C	T	32.252	0.011	0.002	1.40E-08	-0.029	0.027	0.2708
rs7826687	8	126645689	G	C	98.944	-0.021	0.002	2.60E-23	0.020	0.028	0.4772
rs7830404	8	19357346	T	A	37.388	0.012	0.002	9.70E-10	0.001	0.026	0.9587
rs78369186	6	109160436	A	G	30.299	0.026	0.005	3.70E-08	-0.086	0.076	0.257
rs78424224	15	58692777	C	G	30.327	-0.065	0.012	3.70E-08	-0.023	0.325	0.9438
rs784256	18	53398626	A	G	30.067	-0.013	0.002	4.20E-08	0.011	0.034	0.741401
rs78456138	2	163132346	T	C	30.783	0.035	0.006	2.90E-08	-0.104	0.090	0.2489
rs7853377	9	86552205	G	A	43.297	0.015	0.002	4.70E-11	-0.008	0.031	0.7896
rs78548599	9	107592356	T	C	54.733	-0.030	0.004	1.40E-13	0.026	0.069	0.704301
rs78880876	11	57133008	G	A	38.430	-0.017	0.003	5.70E-10	0.014	0.042	0.7279
rs78994850	11	45600598	C	T	43.266	0.033	0.005	4.80E-11	-0.009	0.049	0.8556
rs7903537	10	76847490	T	C	34.068	-0.011	0.002	5.30E-09	-0.020	0.025	0.4223
rs79060184	18	47339822	A	G	32.574	0.038	0.007	1.10E-08	0.042	0.089	0.6383
rs79119207	15	58740443	A	C	78.117	-0.057	0.006	9.70E-19	-0.158	0.107	0.1401
rs79126702	15	58771092	G	T	60.070	-0.034	0.004	9.20E-15	0.111	0.069	0.1071
rs7924036	10	65191645	T	G	52.855	0.014	0.002	3.60E-13	-0.041	0.025	0.1005
rs7925100	11	118941596	A	G	89.773	-0.018	0.002	2.70E-21	0.002	0.028	0.9358
rs79311290	16	85150163	G	A	33.016	-0.018	0.003	9.10E-09	0.024	0.056	0.668599

rs79546662	16	57096098	T	C	215.536	-0.068	0.005	8.50E-49	-0.035	0.064	0.582
rs79634051	11	14561945	C	G	46.532	0.039	0.006	9.00E-12	-0.162	0.070	0.0202302
rs7966846	12	57653582	C	A	79.691	-0.019	0.002	4.40E-19	0.003	0.026	0.9172
rs79968526	16	56911069	C	T	153.985	-0.079	0.006	2.30E-35	0.078	0.068	0.2569
rs80101730	8	9856358	C	T	33.534	-0.033	0.006	7.00E-09	-0.026	0.059	0.654501
rs80120242	3	132235344	T	A	29.772	-0.023	0.004	4.90E-08	0.087	0.053	0.0984804
rs8014289	14	75377352	G	A	61.435	0.015	0.002	4.60E-15	0.026	0.027	0.3401
rs8057340	16	57358500	A	T	34.647	-0.023	0.004	4.00E-09	-0.062	0.044	0.1543
rs8081548	17	7438834	A	T	83.345	0.018	0.002	6.90E-20	-0.028	0.028	0.3123
rs8084730	18	46381865	A	C	30.235	-0.013	0.002	3.80E-08	-0.004	0.032	0.9019
rs8086351	18	47171888	G	C	1121.740	0.084	0.003	1.00E-200	-0.059	0.034	0.0775408
rs8102873	19	57488423	T	C	32.014	-0.011	0.002	1.50E-08	-0.013	0.025	0.6126
rs8126001	20	62712053	T	C	36.511	0.012	0.002	1.50E-09	-0.026	0.025	0.303
rs830620	3	71679148	T	C	59.980	0.015	0.002	9.60E-15	-0.041	0.026	0.1066
rs837500	12	125077567	T	C	188.414	0.027	0.002	7.10E-43	0.038	0.025	0.1383
rs840873	2	129067080	C	T	30.328	-0.027	0.005	3.60E-08	0.014	0.047	0.760901
rs867930	8	19498087	T	A	68.333	-0.018	0.002	1.40E-16	0.060	0.030	0.0430695
rs880674	4	2250109	C	T	29.953	0.015	0.003	4.40E-08	-0.061	0.036	0.0954091
rs890849	19	8478250	C	G	104.655	-0.026	0.003	1.50E-24	0.023	0.036	0.5208
rs893058	9	107663893	A	C	39.732	-0.045	0.007	2.90E-10	-0.012	0.072	0.8625
rs907866	2	20371380	A	G	91.112	-0.018	0.002	1.40E-21	0.016	0.025	0.5299
rs921919	12	125265201	A	G	407.279	-0.042	0.002	1.40E-90	0.043	0.026	0.0923103
rs929166	1	110255174	G	T	34.506	0.012	0.002	4.20E-09	0.015	0.027	0.584
rs9347737	6	163740322	G	A	47.916	-0.013	0.002	4.40E-12	-0.012	0.025	0.640301
rs9355287	6	160847869	C	T	105.887	-0.052	0.005	7.80E-25	0.037	0.046	0.4192
rs9368851	6	35142899	G	A	56.202	0.019	0.003	6.50E-14	-0.005	0.037	0.8902
rs9465693	6	20313120	A	C	33.768	-0.012	0.002	6.20E-09	0.020	0.027	0.4495
rs9471972	6	42915021	A	G	113.007	0.020	0.002	2.20E-26	0.061	0.026	0.0167402
rs947799	11	64851305	T	C	44.670	0.013	0.002	2.30E-11	-0.004	0.026	0.882
rs9534021	13	45589902	A	G	35.941	0.033	0.006	2.00E-09	-0.205	0.092	0.02666
rs9596270	13	50842440	C	T	30.918	0.021	0.004	2.70E-08	-0.117	0.052	0.0245098

rs9604045	13	113927208	T	G	58.544	0.018	0.002	2.00E-14	-0.035	0.034	0.3112
rs9608972	22	30931307	C	T	59.264	-0.017	0.002	1.40E-14	0.005	0.031	0.8802
rs9610329	22	36042986	T	C	31.609	-0.011	0.002	1.90E-08	0.002	0.026	0.9427
rs964184	11	116648917	C	G	1423.587	0.105	0.003	1.00E-200	-0.032	0.036	0.3714
rs9646167	14	81635888	T	C	53.335	-0.014	0.002	2.80E-13	0.027	0.025	0.2917
rs9647335	3	135880410	T	A	131.070	0.028	0.002	2.40E-30	-0.024	0.039	0.5329
rs9650602	8	20441920	T	C	39.424	-0.014	0.002	3.40E-10	-0.005	0.028	0.8511
rs968050	6	98574560	T	C	51.253	0.014	0.002	8.10E-13	-0.023	0.025	0.3608
rs971349	9	106269666	A	C	30.036	0.014	0.003	4.20E-08	-0.021	0.030	0.4811
rs9783411	12	109106537	C	A	32.317	-0.011	0.002	1.30E-08	-0.019	0.026	0.4512
rs9877304	3	196073072	A	G	39.479	-0.014	0.002	3.30E-10	0.001	0.030	0.9624
rs9884482	4	106081636	C	T	40.289	-0.013	0.002	2.20E-10	0.002	0.026	0.9511
rs9913626	17	37797726	A	C	30.072	-0.045	0.008	4.20E-08	0.039	0.048	0.414
rs9953528	18	19664098	A	G	47.625	-0.015	0.002	5.20E-12	-0.001	0.029	0.9763
rs9958734	18	47118398	C	T	212.707	0.070	0.005	3.50E-48	0.004	0.044	0.9365
rs9979895	21	46906488	G	C	46.032	-0.014	0.002	1.20E-11	-0.006	0.030	0.8529
rs998584	6	43757896	A	C	320.515	-0.034	0.002	1.10E-71	0.015	0.025	0.550301
rs9987289	8	9183358	G	A	702.382	0.087	0.003	9.10E-155	0.036	0.038	0.3528
rs9989419	16	56985139	G	A	5569.869	0.144	0.002	1.00E-200	0.055	0.026	0.0333196

SNP	Chr	Position	Effect Allele	Other Allele	F	Association with exposure			Association with Gout		
						**Beta**	**SE**	***P***	**Beta**	**SE**	***P***
**LDL-C**											
rs10029034	4	15096680	T	C	31.031	0.012	0.002	2.50E-08	-0.014	0.029	0.628901
rs1009360	2	65276049	C	T	84.741	-0.018	0.002	3.40E-20	-0.049	0.026	0.0550402
rs10152471	15	101890913	A	G	43.596	-0.014	0.002	4.00E-11	-0.014	0.026	0.580801
rs10172544	2	85788270	A	C	31.040	-0.011	0.002	2.50E-08	0.018	0.026	0.4825
rs10180284	2	50716016	T	C	34.361	-0.012	0.002	4.60E-09	0.011	0.025	0.674599
rs10210970	2	28646847	T	C	63.089	0.023	0.003	2.00E-15	-0.035	0.033	0.2789

rs10219951	13	114522295	T	C	37.875	0.019	0.003	7.50E-10	0.024	0.033	0.4628
rs10242866	7	17920613	T	C	60.599	0.016	0.002	7.00E-15	0.010	0.028	0.7061
rs10261878	7	25950545	C	A	31.648	-0.025	0.004	1.80E-08	-0.122	0.075	0.1056
rs1030472	5	157994544	G	A	86.743	0.023	0.002	1.20E-20	0.021	0.036	0.5667
rs1037117	15	102068658	A	G	56.478	0.017	0.002	5.70E-14	0.006	0.028	0.8407
rs1043764	12	122630915	T	C	43.411	-0.017	0.003	4.40E-11	-0.090	0.028	0.00132501
rs1045241	5	118729286	T	C	84.857	-0.021	0.002	3.20E-20	-0.011	0.027	0.679599
rs10455872	6	161010118	G	A	145.056	-0.044	0.004	2.10E-33	-0.001	0.061	0.9832
rs1057868	7	75615006	T	C	31.852	0.012	0.002	1.70E-08	-0.002	0.026	0.9307
rs1066391	12	67716703	G	A	32.783	-0.012	0.002	1.00E-08	-0.005	0.026	0.8479
rs10750766	11	65473798	A	C	78.540	0.019	0.002	7.80E-19	0.069	0.029	0.01595
rs10773049	12	124506631	C	T	203.256	-0.029	0.002	4.10E-46	-0.058	0.026	0.02374
rs10775406	17	46197755	G	A	78.886	0.021	0.002	6.60E-19	0.067	0.029	0.0184599
rs1077835	15	58723426	G	A	388.053	0.047	0.002	2.20E-86	0.068	0.029	0.0184999
rs10797119	9	92202495	C	T	61.425	0.016	0.002	4.60E-15	0.024	0.025	0.3384
rs10811662	9	22134253	A	G	34.354	-0.015	0.003	4.60E-09	0.084	0.036	0.0196598
rs10863828	1	210592392	G	T	31.102	-0.013	0.002	2.40E-08	-0.003	0.029	0.9248
rs10883451	10	101924418	C	T	29.756	0.011	0.002	4.90E-08	-0.009	0.026	0.7199
rs10899490	11	78105879	T	C	39.874	-0.017	0.003	2.70E-10	0.007	0.032	0.8314
rs11023160	11	14264225	A	T	30.434	0.012	0.002	3.50E-08	0.009	0.026	0.734
rs11030107	11	27694835	G	A	50.521	0.016	0.002	1.20E-12	0.075	0.029	0.00962698
rs11045171	12	20470199	G	A	99.322	-0.025	0.003	2.10E-23	0.072	0.031	0.0193602
rs11057397	12	124419728	T	C	168.279	-0.027	0.002	1.80E-38	-0.010	0.028	0.7309
rs11078597	17	1618363	C	T	56.462	0.019	0.003	5.70E-14	-0.017	0.032	0.6084
rs11097128	4	88068837	A	G	40.028	-0.021	0.003	2.50E-10	-0.053	0.032	0.1026
rs11100083	4	157682598	C	T	45.493	-0.016	0.002	1.50E-11	0.053	0.032	0.1012
rs11118310	1	219637671	T	A	91.446	0.019	0.002	1.10E-21	0.035	0.026	0.1753
rs11122450	1	230301811	G	T	559.145	-0.048	0.002	1.30E-123	-0.014	0.026	0.591701
rs11185542	3	196187927	C	G	31.883	-0.013	0.002	1.60E-08	-0.002	0.028	0.9364
rs111914893	7	74002323	T	C	36.563	0.028	0.005	1.50E-09	0.120	0.090	0.1821

rs11195952	10	114173554	A	G	57.023	-0.023	0.003	4.30E-14	-0.043	0.049	0.3775
rs11206374	1	40048009	A	G	110.937	0.025	0.002	6.10E-26	-0.025	0.031	0.4104
rs11216122	11	116609540	T	G	89.898	-0.043	0.004	2.50E-21	0.055	0.057	0.3324
rs112162280	17	43194413	T	C	33.160	-0.013	0.002	8.50E-09	-0.043	0.027	0.1134
rs112259268	17	41874745	A	C	540.802	0.140	0.006	1.30E-119	-0.089	0.073	0.2218
rs11228377	11	68603346	C	T	58.226	-0.015	0.002	2.30E-14	0.004	0.026	0.876
rs11231161	11	62378221	G	A	65.951	0.017	0.002	4.60E-16	-0.085	0.030	0.00403097
rs112403212	12	125308682	T	C	37.206	0.018	0.003	1.10E-09	0.037	0.033	0.2515
rs11240358	1	205070573	A	G	44.674	0.014	0.002	2.30E-11	0.031	0.026	0.2453
rs113439801	12	62838230	T	C	42.833	-0.017	0.003	6.00E-11	-0.010	0.039	0.7983
rs114473447	6	32518488	A	G	49.037	0.057	0.008	2.50E-12	0.154	0.286	0.589599
rs114741460	6	32596835	T	C	156.896	0.063	0.005	5.40E-36	0.056	0.173	0.7465
rs114796738	3	52757046	T	C	30.641	0.025	0.004	3.10E-08	-0.049	0.070	0.4813
rs114940462	2	27861618	T	G	159.620	-0.061	0.005	1.40E-36	-0.043	0.065	0.5046
rs1149470	8	64608119	A	T	35.589	-0.014	0.002	2.40E-09	0.049	0.031	0.1201
rs115661421	2	26945012	G	A	32.933	0.062	0.011	9.50E-09	0.146	0.090	0.1034
rs11598250	10	94416521	C	G	30.254	0.013	0.002	3.80E-08	-0.066	0.031	0.0331497
rs11620989	14	69746345	G	A	30.776	0.011	0.002	2.90E-08	-0.008	0.026	0.749701
rs11629629	15	43403415	A	G	35.763	0.012	0.002	2.20E-09	0.050	0.027	0.0686894
rs11635675	15	63793238	G	T	132.053	0.024	0.002	1.50E-30	0.017	0.026	0.5208
rs11643980	16	85275894	G	T	30.694	-0.014	0.003	3.00E-08	-0.010	0.031	0.7545
rs11657201	17	599924	G	A	31.258	0.013	0.002	2.30E-08	0.023	0.034	0.501

rs11688682	2	121347612	C	G	41.552	-0.015	0.002	1.10E-10	-0.059	0.030	0.04735
rs116919951	8	19933689	C	T	31.917	0.047	0.008	1.60E-08	-0.030	0.086	0.723801
rs116995028	11	116999072	T	C	400.245	0.177	0.009	4.90E-89	-0.039	0.113	0.733199
rs116998829	8	19520669	G	A	33.653	-0.029	0.005	6.60E-09	-0.034	0.093	0.715801
rs117001569	8	19574920	G	C	75.575	-0.094	0.011	3.50E-18	-0.027	0.092	0.7703
rs11709868	3	142648844	T	G	42.818	-0.014	0.002	6.00E-11	-0.058	0.029	0.0456405
rs1171617	10	61467182	T	G	45.073	0.016	0.002	1.90E-11	0.116	0.035	0.000865705
rs117233107	12	4328521	A	G	72.305	-0.073	0.009	1.80E-17	-0.204	0.108	0.0583795
rs117291242	11	380315	T	C	31.795	0.030	0.005	1.70E-08	0.064	0.057	0.2585
rs117350303	8	126466408	G	A	46.939	-0.073	0.011	7.30E-12	0.142	0.095	0.1374
rs117619191	11	117248283	C	T	138.971	0.082	0.007	4.50E-32	-0.102	0.129	0.4286
rs117664196	8	126673909	C	T	39.445	-0.038	0.006	3.40E-10	-0.055	0.120	0.6498
rs11769620	7	72995749	G	A	33.764	0.023	0.004	6.20E-09	-0.008	0.054	0.8769
rs117781948	8	126351023	A	G	43.997	0.057	0.009	3.30E-11	0.038	0.159	0.809
rs1178982	7	72851167	C	T	131.139	-0.043	0.004	2.30E-30	0.009	0.057	0.882
rs117956669	8	19847645	G	T	34.585	0.054	0.009	4.10E-09	-0.109	0.107	0.3066
rs118188894	11	57142624	C	T	35.442	0.019	0.003	2.60E-09	0.001	0.053	0.9821
rs11903847	2	25592918	C	T	39.468	-0.013	0.002	3.30E-10	0.004	0.031	0.8872
rs11925442	3	170692496	C	T	62.994	0.024	0.003	2.10E-15	0.041	0.037	0.2668

rs11980456	7	29319249	A	G	31.366	0.012	0.002	2.10E-08	0.011	0.028	0.701799
rs11992444	8	25464690	T	G	49.869	-0.014	0.002	1.60E-12	0.065	0.026	0.0124099
rs12140153	1	62579891	T	G	40.163	-0.022	0.003	2.30E-10	0.082	0.047	0.0803804
rs12185242	17	47407071	C	A	76.873	0.018	0.002	1.80E-18	0.091	0.025	0.000316403
rs12306007	12	47184042	T	C	30.131	0.016	0.003	4.00E-08	0.002	0.036	0.9585
rs12369443	12	20582651	G	A	30.355	-0.014	0.003	3.60E-08	-0.012	0.029	0.6853
rs12415159	10	113978850	G	A	55.819	0.021	0.003	7.90E-14	-0.001	0.038	0.9822
rs12422600	12	54429385	A	G	36.668	-0.013	0.002	1.40E-09	-0.038	0.026	0.1431
rs12424054	12	107186747	A	G	66.011	0.019	0.002	4.50E-16	-0.003	0.033	0.925
rs12440800	15	61960302	T	A	49.302	0.016	0.002	2.20E-12	0.010	0.031	0.7491
rs12446515	16	56987015	T	C	244.835	-0.033	0.002	3.50E-55	0.018	0.028	0.5251
rs12449427	17	7173279	C	G	35.877	-0.014	0.002	2.10E-09	0.004	0.029	0.9013
rs12470242	2	28546023	A	G	78.325	0.023	0.003	8.70E-19	0.033	0.028	0.2355
rs12472667	2	171629063	G	C	44.517	0.014	0.002	2.50E-11	0.035	0.027	0.192
rs12479355	2	227226952	G	A	61.864	-0.019	0.002	3.70E-15	-0.012	0.033	0.708101
rs12504746	4	39646631	T	C	36.662	-0.015	0.003	1.40E-09	-0.051	0.035	0.1485
rs12530679	7	106632113	G	A	36.173	-0.012	0.002	1.80E-09	-0.018	0.025	0.4702
rs12574790	11	116575687	T	C	318.819	0.184	0.010	2.60E-71	0.142	0.169	0.4014
rs12591786	15	60902512	T	C	40.580	-0.018	0.003	1.90E-10	0.009	0.042	0.8253
rs12669911	7	32262377	C	A	33.303	-0.012	0.002	7.90E-09	-0.023	0.028	0.4122
rs12791103	11	116673315	T	G	465.377	-0.072	0.003	3.30E-103	-0.065	0.039	0.0999793
rs12824533	12	11791685	G	A	39.724	0.013	0.002	2.90E-10	-0.007	0.026	0.7758
rs12880341	14	64236191	C	T	58.722	0.021	0.003	1.80E-14	-0.025	0.037	0.5113
rs12902047	15	41915522	C	A	36.299	-0.013	0.002	1.70E-09	-0.005	0.027	0.8387
rs12926107	16	88004092	G	A	39.864	0.013	0.002	2.70E-10	0.025	0.027	0.3621
rs12928099	16	15150505	A	C	167.268	-0.028	0.002	2.90E-38	-0.063	0.028	0.02512
rs12944954	17	7485131	G	A	37.185	0.040	0.007	1.10E-09	-0.003	0.084	0.9694
rs13064956	3	12170251	C	T	40.096	-0.020	0.003	2.40E-10	-0.029	0.038	0.4452
rs13066793	3	87037543	G	A	41.821	-0.022	0.003	1.00E-10	0.058	0.047	0.2129
rs13101828	4	965720	G	A	35.000	-0.012	0.002	3.30E-09	-0.019	0.025	0.4637
rs13107325	4	103188709	T	C	63.389	0.030	0.004	1.70E-15	0.046	0.108	0.668399

rs13108218	4	3443931	G	A	219.859	-0.031	0.002	9.70E-50	0.037	0.027	0.1752
rs13251367	8	72001931	C	T	30.227	-0.011	0.002	3.80E-08	0.024	0.026	0.3666
rs13269725	8	72459889	G	A	90.374	0.035	0.004	2.00E-21	0.019	0.067	0.782
rs13270882	8	19960861	T	C	39.109	0.030	0.005	4.00E-10	0.032	0.062	0.6031
rs13389219	2	165528876	T	C	344.516	-0.038	0.002	6.60E-77	-0.029	0.027	0.2808
rs1340819	13	29145323	C	A	33.729	-0.012	0.002	6.30E-09	0.023	0.027	0.4005
rs1347188	4	124743259	G	A	36.099	0.014	0.002	1.90E-09	0.064	0.029	0.0257199
rs1351394	12	66351826	C	T	45.647	0.013	0.002	1.40E-11	-0.029	0.025	0.2604
rs138191773	6	72522321	A	G	35.485	-0.047	0.008	2.60E-09	-0.040	0.208	0.8458
rs138751626	15	57154952	T	A	57.569	0.028	0.004	3.30E-14	0.083	0.064	0.1982
rs139076463	1	63110217	C	T	35.326	0.040	0.007	2.80E-09	-0.028	0.077	0.7175
rs139098834	2	28180473	A	G	43.808	-0.044	0.007	3.60E-11	-0.139	0.076	0.0665595
rs139453187	7	72155834	C	T	35.704	0.023	0.004	2.30E-09	-0.046	0.049	0.3481
rs139974673	15	44027885	C	T	525.934	0.143	0.006	2.20E-116	-0.229	0.177	0.1968
rs140107293	10	5267191	G	A	67.993	-0.023	0.003	1.60E-16	-0.019	0.037	0.611299
rs141415727	2	28221157	A	G	32.861	0.046	0.008	9.90E-09	0.013	0.156	0.9339
rs141469619	11	116714293	G	A	980.133	0.332	0.011	1.00E-200	-0.343	0.203	0.0909997
rs142848030	11	116871959	T	C	41.589	-0.047	0.007	1.10E-10	-0.107	0.088	0.2266
rs143076454	16	921179	A	G	30.183	0.040	0.007	3.90E-08	0.257	0.108	0.01736
rs14417164	1	63310797	A	T	41.246	-0.051	0.008	1.30E-10	0.013	0.121	0.9148

5											
rs144246507	2	28297334	T	A	30.157	-0.042	0.008	4.00E-08	0.078	0.148	0.5967
rs144423449	2	27289677	A	G	42.841	-0.054	0.008	5.90E-11	-0.277	0.197	0.1595
rs144469617	8	19903238	A	G	43.461	0.052	0.008	4.30E-11	-0.110	0.159	0.490399
rs144470864	2	21183334	C	A	51.010	0.031	0.004	9.20E-13	0.042	0.050	0.4082
rs145692474	2	27712638	A	G	38.816	-0.079	0.013	4.70E-10	0.025	0.200	0.8995
rs145775785	12	65902265	T	C	34.528	-0.050	0.009	4.20E-09	-0.110	0.068	0.1046
rs146203232	6	160543148	T	C	115.375	0.041	0.004	6.50E-27	-0.031	0.054	0.5606
rs148048657	8	19774005	A	G	32.097	0.038	0.007	1.50E-08	-0.044	0.085	0.6102
rs148482297	2	28415598	T	C	90.163	0.060	0.006	2.20E-21	0.054	0.125	0.6648
rs149214447	2	27102554	G	C	57.849	0.061	0.008	2.80E-14	0.070	0.252	0.780001
rs1495741	8	18272881	A	G	249.062	-0.038	0.002	4.20E-56	-0.039	0.029	0.1824
rs149719228	8	126567164	A	G	39.218	0.057	0.009	3.80E-10	0.159	0.154	0.3
rs149778057	13	31007977	C	A	50.110	-0.016	0.002	1.50E-12	-0.030	0.025	0.2385
rs1508103	11	116379978	G	A	101.690	0.042	0.004	6.50E-24	-0.033	0.065	0.604901
rs1534696	7	26397239	A	C	98.535	-0.020	0.002	3.20E-23	-0.010	0.026	0.686099
rs1552781	8	19624132	G	C	191.749	-0.033	0.002	1.30E-43	-0.016	0.028	0.5514
rs1556124	13	110991189	A	G	32.646	0.014	0.002	1.10E-08	0.012	0.034	0.7345
rs1561928	8	129568061	G	A	35.420	0.018	0.003	2.70E-09	-0.014	0.038	0.7185

rs1567353	9	1033773	G	C	46.920	0.015	0.002	7.40E-12	0.017	0.027	0.5252
rs157935	7	130585553	G	T	43.309	-0.014	0.002	4.70E-11	-0.026	0.026	0.3228
rs17146641	4	69555968	A	G	31.623	0.016	0.003	1.90E-08	-0.030	0.040	0.450001
rs1728407	16	86422112	G	A	33.987	0.012	0.002	5.50E-09	0.017	0.026	0.500299
rs17326656	2	48962291	T	G	55.846	0.017	0.002	7.80E-14	0.032	0.034	0.3431
rs174566	11	61592362	G	A	543.834	0.049	0.002	2.80E-120	-0.009	0.026	0.7405
rs17585887	6	139835498	C	T	200.933	-0.029	0.002	1.30E-45	0.010	0.025	0.6977
rs1760801	1	154259650	A	G	86.345	-0.020	0.002	1.50E-20	0.005	0.026	0.8354
rs1799831	7	44199142	T	C	80.019	0.025	0.003	3.70E-19	0.000	0.032	0.9923
rs1801689	17	64210580	C	A	131.027	-0.066	0.006	2.40E-30	-0.041	0.131	0.757
rs1823227	3	172240215	G	T	34.062	-0.013	0.002	5.30E-09	0.009	0.031	0.7703
rs1835346	6	161162290	G	A	35.832	-0.039	0.007	2.20E-09	0.062	0.104	0.550201
rs1840339	10	56634942	T	G	29.959	-0.012	0.002	4.40E-08	0.012	0.027	0.6693
rs184694823	1	150642998	G	A	31.946	0.021	0.004	1.60E-08	0.018	0.056	0.7524
rs185139895	6	34185863	A	G	79.765	0.043	0.005	4.20E-19	0.224	0.078	0.00386002
rs186696265	6	161111700	T	C	158.530	-0.104	0.008	2.40E-36	0.001	0.099	0.9939
rs1872992	12	26457190	G	A	40.154	0.015	0.002	2.30E-10	0.018	0.028	0.525
rs187544997	8	19890641	G	C	44.288	0.053	0.008	2.80E-11	0.037	0.261	0.8881
rs187929675	11	117111368	T	C	382.682	-0.171	0.009	3.20E-85	-0.240	0.120	0.0462498
rs193735	5	130672958	A	G	38.734	0.033	0.005	4.90E-10	0.007	0.050	0.8959
rs1938566	1	98478981	T	C	63.095	-0.021	0.003	2.00E-15	0.005	0.030	0.8759
rs2017500	15	99196112	A	G	33.960	0.012	0.002	5.60E-09	-0.018	0.025	0.492
rs2035816	4	100508556	G	A	60.941	-0.028	0.004	5.90E-15	0.058	0.074	0.4286
rs2043085	15	58680954	C	T	227.642	-0.031	0.002	1.90E-51	0.038	0.026	0.1385
rs2068888	10	94839642	A	G	254.197	-0.032	0.002	3.20E-57	-0.002	0.025	0.9318
rs2081687	8	59388565	C	T	156.216	-0.026	0.002	7.60E-36	-0.026	0.026	0.3161

rs2131311	1	40435999	G	A	31.079	-0.012	0.002	2.50E-08	0.023	0.028	0.4082
rs2131919	9	95283887	G	A	41.793	0.017	0.003	1.00E-10	0.007	0.033	0.8196
rs213209	6	33176958	T	C	30.921	0.012	0.002	2.70E-08	-0.022	0.028	0.4403
rs213494	1	54877103	T	C	56.556	0.016	0.002	5.50E-14	0.016	0.028	0.559899
rs2137557	3	188439236	C	T	31.978	0.012	0.002	1.60E-08	0.051	0.027	0.0557699
rs2165557	8	19835050	T	A	336.025	-0.047	0.003	4.70E-75	-0.041	0.034	0.227
rs2237029	4	55535336	A	G	46.685	-0.014	0.002	8.30E-12	0.032	0.026	0.222
rs2240466	7	72856269	A	G	1654.249	-0.123	0.003	1.00E-200	-0.091	0.038	0.01661
rs2240533	14	71541026	C	T	36.423	-0.013	0.002	1.60E-09	0.043	0.028	0.1226
rs2244278	9	107661129	A	C	77.736	-0.027	0.003	1.20E-18	0.013	0.044	0.763199
rs2276853	3	47282303	A	G	30.243	0.011	0.002	3.80E-08	0.043	0.026	0.0963097
rs2277083	6	133827354	G	A	58.723	-0.015	0.002	1.80E-14	-0.017	0.025	0.490399
rs2288004	16	31054040	C	G	54.031	-0.015	0.002	2.00E-13	0.020	0.026	0.4411
rs2302104	5	140047600	T	C	36.148	-0.014	0.002	1.80E-09	0.032	0.029	0.264
rs2302364	11	62190700	C	T	34.641	0.016	0.003	4.00E-09	0.041	0.037	0.2704
rs2382825	2	219184275	T	C	43.256	-0.013	0.002	4.80E-11	-0.055	0.029	0.0556596
rs2407278	8	37399290	G	A	34.022	-0.034	0.006	5.40E-09	0.006	0.057	0.9106
rs241771	17	26592946	C	T	32.136	0.011	0.002	1.40E-08	-0.015	0.025	0.5449
rs2420477	10	120242187	C	T	31.339	-0.011	0.002	2.20E-08	0.020	0.025	0.4297
rs2439364	15	66876675	G	A	45.176	-0.013	0.002	1.80E-11	0.002	0.025	0.9279
rs245051	5	149345975	G	A	36.018	-0.012	0.002	2.00E-09	-0.051	0.026	0.0534404
rs2455821	3	15681940	A	C	36.283	0.013	0.002	1.70E-09	0.020	0.029	0.4977
rs2487294	10	113937941	T	G	68.041	0.018	0.002	1.60E-16	0.032	0.027	0.2393
rs2517887	6	29807393	C	G	36.368	0.015	0.002	1.60E-09	-0.058	0.031	0.0623993
rs2519093	9	136141870	T	C	68.207	-0.021	0.003	1.50E-16	-0.045	0.031	0.1554
rs2528787	13	112188236	T	C	30.241	-0.011	0.002	3.80E-08	0.008	0.027	0.754899
rs268	8	19813529	G	A	941.512	0.227	0.007	1.00E-200	-0.049	0.085	0.566601
rs275184	15	39444679	G	T	39.723	-0.017	0.003	2.90E-10	0.017	0.032	0.582599
rs2761294	10	95067043	A	G	82.739	0.019	0.002	9.40E-20	-0.003	0.026	0.8974
rs2773469	10	115798895	G	A	70.083	-0.019	0.002	5.70E-17	0.004	0.029	0.8845
rs278981	4	40428010	C	T	30.197	0.013	0.002	3.90E-08	-0.029	0.030	0.3373

rs2800713	6	127383980	A	G	50.010	0.031	0.004	1.50E-12	-0.039	0.046	0.3988
rs2812208	13	50707087	C	G	48.366	-0.048	0.007	3.50E-12	-0.125	0.087	0.1496
rs28383314	6	32587213	C	T	342.908	0.038	0.002	1.50E-76	-0.031	0.030	0.2936
rs28481932	6	31242900	G	A	54.018	0.022	0.003	2.00E-13	0.050	0.039	0.2041
rs28546518	8	19934043	G	A	69.423	-0.025	0.003	7.90E-17	0.039	0.032	0.2281
rs28577186	16	4488191	A	G	59.611	-0.016	0.002	1.20E-14	-0.034	0.026	0.1941
rs28624578	15	31637666	C	T	32.022	0.015	0.003	1.50E-08	0.028	0.033	0.4035
rs28732146	6	31561353	A	T	110.229	0.026	0.002	8.70E-26	0.030	0.033	0.3695
rs28873097	4	88408172	C	T	31.137	0.011	0.002	2.40E-08	-0.026	0.025	0.3027
rs2917677	16	69750849	T	C	87.876	-0.019	0.002	7.00E-21	-0.066	0.026	0.00911906
rs2925979	16	81534790	C	T	220.120	-0.032	0.002	8.50E-50	0.006	0.027	0.8261
rs2943645	2	227099180	T	C	378.320	0.040	0.002	2.90E-84	0.052	0.026	0.04917
rs2954011	8	126424379	T	C	54.882	-0.015	0.002	1.30E-13	-0.002	0.026	0.9532
rs2983896	6	97029871	A	G	32.429	0.014	0.002	1.20E-08	-0.014	0.032	0.658901
rs308	8	19817476	G	T	520.939	-0.159	0.007	2.60E-115	0.082	0.074	0.271
rs3097858	5	155571886	T	A	34.523	0.020	0.003	4.20E-09	-0.009	0.041	0.8276
rs3103310	3	12473045	G	A	73.255	0.020	0.002	1.10E-17	-0.019	0.034	0.5673
rs320369	1	118143517	G	A	34.116	-0.013	0.002	5.20E-09	-0.031	0.026	0.2309
rs325485	5	103995368	G	A	33.125	-0.012	0.002	8.60E-09	-0.032	0.027	0.2331
rs326222	11	47259668	C	T	136.713	0.025	0.002	1.40E-31	-0.009	0.027	0.7274
rs3289	8	19823192	C	T	645.385	0.154	0.006	2.30E-142	-0.092	0.092	0.3148
rs34144542	11	116774447	G	A	104.507	-0.039	0.004	1.60E-24	0.026	0.053	0.6265
rs34196215	5	55965964	C	T	39.385	-0.018	0.003	3.50E-10	-0.022	0.039	0.5799
rs34234296	2	175166636	A	G	30.954	-0.011	0.002	2.60E-08	-0.011	0.028	0.702301
rs343	8	19810787	A	C	1535.785	-0.141	0.004	1.00E-200	-0.012	0.052	0.8243
rs34580448	5	82810884	C	T	46.234	-0.034	0.005	1.00E-11	0.111	0.094	0.2381
rs34893217	8	16279180	T	G	31.820	-0.018	0.003	1.70E-08	-0.057	0.042	0.17
rs35104374	12	6739497	C	T	48.157	-0.016	0.002	3.90E-12	0.024	0.030	0.4246
rs35169799	11	64031241	T	C	187.866	0.056	0.004	9.30E-43	0.059	0.043	0.1752
rs35200015	11	117383215	A	G	47.029	0.017	0.003	7.00E-12	-0.051	0.028	0.0704904
rs35477346	15	40400195	C	T	38.944	0.014	0.002	4.40E-10	0.000	0.027	0.9967

rs35763453	12	46215895	C	T	42.748	0.028	0.004	6.20E-11	0.070	0.067	0.2979
rs35859536	8	118191475	T	C	44.891	-0.014	0.002	2.10E-11	0.005	0.026	0.8602
rs36043408	1	203511492	A	G	41.606	-0.013	0.002	1.10E-10	0.013	0.026	0.621901
rs36061954	8	38329650	T	C	33.709	0.012	0.002	6.40E-09	-0.010	0.026	0.695
rs3731696	2	203431804	G	A	52.150	0.022	0.003	5.10E-13	0.126	0.038	0.00107701
rs3738182	1	221057662	A	G	36.565	0.015	0.003	1.50E-09	0.008	0.030	0.7931
rs3758413	10	17268839	C	T	30.392	0.011	0.002	3.50E-08	-0.010	0.026	0.7082
rs3775228	4	87985166	T	C	275.915	0.034	0.002	5.80E-62	-0.034	0.026	0.1844
rs37774222<br>8	1	149844763	G	A	46.919	-0.025	0.004	7.40E-12	0.059	0.055	0.2827
rs3784310	15	72103427	C	T	34.321	-0.013	0.002	4.70E-09	-0.034	0.027	0.2142
rs3794695	16	72097827	T	C	120.748	0.028	0.003	4.30E-28	-0.014	0.032	0.657201
rs3808477	8	116670347	T	C	36.904	-0.013	0.002	1.20E-09	-0.044	0.027	0.1072
rs3814883	16	29994922	T	C	55.638	0.015	0.002	8.70E-14	0.062	0.026	0.0149101
rs38189	7	15903403	T	A	55.126	-0.015	0.002	1.10E-13	-0.013	0.027	0.617999
rs3820897	2	3642361	C	T	57.432	0.020	0.003	3.50E-14	0.059	0.027	0.0284702
rs3826043	15	73618238	T	C	36.335	-0.012	0.002	1.70E-09	-0.077	0.026	0.00249201
rs3829126	10	74714177	T	G	45.295	0.023	0.003	1.70E-11	-0.042	0.052	0.4211
rs3936511	5	55860781	G	A	334.623	0.046	0.003	9.50E-75	0.057	0.037	0.125
rs3974807	11	49161967	T	C	39.843	0.016	0.003	2.80E-10	0.050	0.037	0.1787
rs40270	5	55804552	C	A	227.216	0.036	0.002	2.40E-51	-0.005	0.028	0.852
rs4128205	2	165467068	C	A	33.244	0.012	0.002	8.10E-09	0.005	0.026	0.833
rs4134963	6	20486798	T	C	55.584	-0.019	0.003	9.00E-14	-0.019	0.032	0.5566
rs41785	7	116486020	A	C	55.749	-0.015	0.002	8.20E-14	-0.052	0.026	0.0441205
rs4382584	9	139386138	A	G	33.525	0.013	0.002	7.00E-09	-0.028	0.029	0.3347
rs4387830	2	21423281	A	G	68.531	0.027	0.003	1.20E-16	0.039	0.047	0.4014
rs4391855	11	116350751	A	G	133.262	0.059	0.005	7.90E-31	0.000	0.077	0.996
rs4445477	1	219759481	A	G	46.287	-0.016	0.002	1.00E-11	-0.021	0.031	0.4928
rs4471666	16	85198965	G	T	32.058	-0.022	0.004	1.50E-08	-0.028	0.045	0.539299
rs4559942	17	73309269	A	G	32.654	0.014	0.002	1.10E-08	0.069	0.033	0.0364502
rs4564007	9	134854280	C	T	33.336	-0.012	0.002	7.80E-09	0.004	0.028	0.8725

rs4662414	2	145818432	G	A	36.645	-0.012	0.002	1.40E-09	-0.058	0.026	0.0234801
rs4665972	2	27598097	C	T	2424.998	-0.100	0.002	1.00E-200	-0.128	0.026	9.03E-07
rs4675812	2	242395674	A	G	50.037	-0.014	0.002	1.50E-12	-0.023	0.026	0.379
rs4699983	5	55992718	G	A	43.754	-0.013	0.002	3.70E-11	-0.010	0.025	0.6822
rs4709746	6	164133001	T	C	42.508	-0.019	0.003	7.00E-11	-0.003	0.047	0.9497
rs4714001	6	36638175	A	G	35.810	0.012	0.002	2.20E-09	-0.005	0.027	0.8578
rs4731701	7	130430930	T	C	268.256	-0.033	0.002	2.70E-60	-0.006	0.025	0.807
rs4760254	12	57766392	C	G	146.518	-0.028	0.002	1.00E-33	-0.097	0.030	0.00115199
rs4761234	12	69732105	C	T	49.474	-0.014	0.002	2.00E-12	-0.024	0.026	0.3421
rs478420	8	17792853	A	G	38.886	0.013	0.002	4.50E-10	-0.007	0.026	0.793299
rs4793090	17	40686342	A	G	47.342	-0.014	0.002	6.00E-12	0.009	0.026	0.7229
rs480823	11	116525730	C	T	1752.523	0.156	0.004	1.00E-200	0.004	0.035	0.9002
rs4846857	1	230436175	G	A	63.507	-0.023	0.003	1.60E-15	-0.040	0.029	0.1754
rs4909945	11	10673739	C	T	39.199	0.013	0.002	3.80E-10	0.003	0.027	0.9093
rs4938277	11	116397532	T	C	67.313	0.018	0.002	2.30E-16	0.011	0.027	0.6822
rs4969179	17	76391454	G	T	75.993	-0.018	0.002	2.80E-18	0.083	0.028	0.00264198
rs4976033	5	67714246	G	A	75.186	0.018	0.002	4.30E-18	0.005	0.026	0.8528
rs498475	7	28256240	A	G	32.100	-0.012	0.002	1.50E-08	-0.036	0.027	0.1781
rs499293	11	30504660	A	G	31.859	-0.012	0.002	1.70E-08	-0.080	0.026	0.00217901
rs534417	1	23784965	G	A	42.692	0.020	0.003	6.40E-11	-0.033	0.040	0.4011
rs535241194	7	150299483	G	A	73.368	0.021	0.002	1.10E-17	0.027	0.027	0.3183
rs536338	13	51103461	G	C	39.888	0.014	0.002	2.70E-10	-0.046	0.030	0.1248
rs540658636	7	72792387	C	T	38.976	0.033	0.005	4.30E-10	0.088	0.048	0.0658506
rs55646464	5	173324971	T	G	31.559	0.012	0.002	1.90E-08	-0.045	0.026	0.0759994
rs55692143	8	19990450	C	T	30.319	0.017	0.003	3.70E-08	0.020	0.038	0.5949
rs55697600	6	32604166	G	A	174.425	0.068	0.005	8.00E-40	-0.108	0.068	0.1124
rs55802560	10	94558127	C	T	40.129	-0.021	0.003	2.40E-10	-0.041	0.041	0.3235
rs55931203	17	65857714	T	C	85.002	0.024	0.003	3.00E-20	0.008	0.030	0.7798
rs55951611	7	73126343	A	G	64.525	-0.039	0.005	9.50E-16	0.014	0.071	0.8398

rs56057635	11	117349401	G	A	32.952	0.017	0.003	9.40E-09	-0.026	0.043	0.5509
rs56311339	11	116566161	T	C	137.234	-0.037	0.003	1.10E-31	0.052	0.041	0.2034
rs563296	10	99772404	A	G	67.708	0.016	0.002	1.90E-16	0.055	0.025	0.0300497
rs56902258	14	23733114	A	T	36.316	-0.015	0.003	1.70E-09	0.045	0.032	0.1599
rs56959712	12	123188475	T	G	79.740	-0.022	0.002	4.30E-19	-0.025	0.032	0.436
rs5743840	3	52260212	A	T	34.540	0.037	0.006	4.20E-09	0.104	0.157	0.5094
rs575623	11	116278812	T	C	46.165	0.022	0.003	1.10E-11	0.066	0.048	0.176
rs58038553	15	58574324	G	A	47.096	-0.020	0.003	6.80E-12	0.101	0.042	0.0174699
rs581080	9	15305378	C	G	46.078	0.018	0.003	1.10E-11	-0.025	0.036	0.4895
rs5880	16	57015091	C	G	86.511	0.040	0.004	1.40E-20	0.019	0.081	0.8139
rs58839393	2	43490619	T	A	41.662	0.017	0.003	1.10E-10	-0.012	0.034	0.725
rs595767	17	46957987	G	A	46.686	0.014	0.002	8.30E-12	-0.035	0.025	0.1627
rs612874	2	219492924	C	T	80.286	0.018	0.002	3.20E-19	-0.029	0.026	0.2568
rs61780049	1	39363294	G	A	30.097	0.015	0.003	4.10E-08	-0.023	0.039	0.5576
rs61785481	1	110497808	A	G	30.233	0.011	0.002	3.80E-08	-0.018	0.026	0.480799
rs61821643	1	219268532	C	T	32.322	0.037	0.007	1.30E-08	-0.132	0.142	0.3538
rs61830291	1	221001142	C	A	72.380	0.029	0.003	1.80E-17	-0.013	0.040	0.750699
rs61885960	11	14819828	A	T	50.224	-0.031	0.004	1.40E-12	0.017	0.045	0.7094
rs61904855	11	116476668	A	C	83.507	0.054	0.006	6.40E-20	-0.003	0.101	0.9748
rs61907563	11	116733565	A	G	149.805	-0.068	0.006	1.90E-34	0.066	0.052	0.2044
rs61975915	14	58665283	T	C	29.964	-0.012	0.002	4.40E-08	-0.015	0.029	0.6167
rs61993685	14	100765823	C	T	39.176	-0.023	0.004	3.90E-10	-0.054	0.047	0.2538
rs62064941	16	58834402	C	A	30.849	-0.029	0.005	2.80E-08	-0.131	0.084	0.1196
rs62271373	3	150066540	A	T	96.769	0.042	0.004	7.80E-23	-0.011	0.062	0.8569
rs62274099	3	156810578	T	C	35.661	0.012	0.002	2.30E-09	0.003	0.026	0.9236
rs62397245	5	176750688	G	C	39.151	0.015	0.002	3.90E-10	0.027	0.029	0.3574
rs62427982	6	107437166	T	C	39.007	-0.013	0.002	4.20E-10	-0.012	0.026	0.6436
rs62463387	7	72349865	A	G	40.933	0.034	0.005	1.60E-10	-0.018	0.055	0.742699
rs62492368	7	150537635	A	G	42.662	0.014	0.002	6.50E-11	0.038	0.027	0.1509
rs62565259	9	102162570	T	C	41.281	-0.017	0.003	1.30E-10	0.029	0.037	0.437
rs6458869	6	52630269	A	C	73.714	-0.018	0.002	9.00E-18	0.002	0.026	0.929

rs6465120	7	76036364	G	A	40.670	-0.013	0.002	1.80E-10	0.025	0.026	0.3265
rs647071	11	111661455	G	C	55.721	-0.015	0.002	8.40E-14	-0.029	0.026	0.262
rs6494391	15	63406785	C	T	60.990	-0.019	0.002	5.70E-15	0.038	0.028	0.1793
rs6532798	4	100054827	T	C	40.638	0.014	0.002	1.80E-10	-0.015	0.028	0.5921
rs6562773	13	74188935	G	A	35.943	-0.012	0.002	2.00E-09	-0.030	0.025	0.2421
rs6572807	14	52480621	G	A	30.773	0.012	0.002	2.90E-08	0.017	0.028	0.5568
rs66884613	2	28046719	G	A	42.187	-0.045	0.007	8.30E-11	-0.194	0.176	0.2705
rs6690181	1	228056868	C	T	46.111	-0.014	0.002	1.10E-11	0.011	0.026	0.6576
rs6700266	1	178508930	A	G	35.378	-0.012	0.002	2.70E-09	-0.056	0.026	0.0289501
rs6708682	2	64976815	G	A	36.922	0.013	0.002	1.20E-09	0.010	0.028	0.727601
rs6708784	2	111927379	G	A	42.311	-0.013	0.002	7.80E-11	-0.049	0.025	0.0517297
rs676210	2	21231524	A	G	900.649	-0.074	0.002	7.10E-198	0.019	0.029	0.519
rs6792725	3	24520283	G	A	47.263	-0.015	0.002	6.20E-12	-0.008	0.026	0.7605
rs67981690	12	21343886	G	A	101.194	0.030	0.003	8.30E-24	-0.072	0.032	0.0259699
rs6800707	3	52516293	G	C	138.614	0.030	0.003	5.30E-32	0.061	0.043	0.1573
rs6805924	3	69879670	T	G	29.905	0.011	0.002	4.50E-08	-0.003	0.026	0.905
rs684773	3	135956305	C	A	153.847	0.029	0.002	2.50E-35	0.020	0.035	0.5752
rs6865870	5	144498018	G	A	31.762	0.012	0.002	1.70E-08	0.026	0.028	0.3424
rs6882076	5	156390297	C	T	257.891	0.033	0.002	4.90E-58	-0.054	0.027	0.04561
rs6913325	6	106378009	T	G	30.337	-0.011	0.002	3.60E-08	-0.031	0.026	0.2457
rs6924805	6	18747705	T	G	30.502	-0.011	0.002	3.30E-08	-0.006	0.026	0.8342
rs696825	9	86583076	T	C	78.619	-0.020	0.002	7.50E-19	-0.005	0.027	0.8571
rs6968865	7	17287269	T	A	54.190	0.015	0.002	1.80E-13	-0.048	0.027	0.0717596
rs6977665	7	116976830	G	A	45.256	0.014	0.002	1.70E-11	-0.017	0.025	0.506001
rs698927	1	93836218	C	A	51.013	-0.018	0.003	9.20E-13	-0.018	0.033	0.592
rs6995541	8	10671260	G	A	158.303	0.028	0.002	2.70E-36	0.032	0.029	0.2658
rs6999569	8	126475770	G	A	1889.263	-0.086	0.002	1.00E-200	-0.005	0.025	0.8316
rs7077812	10	81096071	C	T	32.234	0.014	0.003	1.40E-08	0.036	0.032	0.2487
rs7090871	10	63830286	C	T	41.781	-0.013	0.002	1.00E-10	-0.017	0.026	0.516799
rs7096101	10	94362928	A	G	42.837	-0.013	0.002	5.90E-11	0.011	0.026	0.6665
rs7104819	11	118769442	G	A	31.491	0.011	0.002	2.00E-08	0.024	0.026	0.3559

rs7124396	11	47814280	G	T	31.234	0.011	0.002	2.30E-08	-0.045	0.026	0.0861708
rs7140110	13	114544024	C	T	169.021	0.028	0.002	1.20E-38	0.037	0.028	0.1856
rs71473777	10	77217080	G	A	37.004	0.019	0.003	1.20E-09	-0.026	0.039	0.5136
rs71538127	7	1010801	G	C	33.769	0.018	0.003	6.20E-09	-0.018	0.043	0.6742
rs71603401	4	18034463	G	A	82.496	0.026	0.003	1.10E-19	0.034	0.047	0.4767
rs7210745	17	74230635	C	T	36.226	0.022	0.004	1.80E-09	-0.084	0.061	0.1681
rs7215055	17	17458353	G	A	90.099	0.039	0.004	2.30E-21	0.036	0.047	0.436
rs7244	5	153800513	A	G	33.697	0.015	0.003	6.40E-09	-0.018	0.033	0.589599
rs72669514	1	63194030	T	C	65.274	0.038	0.005	6.50E-16	-0.037	0.061	0.546
rs72691637	8	144306970	A	G	36.146	-0.015	0.003	1.80E-09	0.020	0.031	0.5218
rs72801474	5	132444128	A	G	80.061	-0.031	0.003	3.60E-19	0.077	0.055	0.1614
rs72810512	2	28192597	T	C	59.779	-0.028	0.004	1.10E-14	0.049	0.047	0.3024
rs72904737	1	51351846	A	G	59.539	-0.027	0.004	1.20E-14	-0.008	0.055	0.888
rs729761	6	43804571	G	T	64.521	0.018	0.002	9.50E-16	0.062	0.028	0.0261999
rs73190070	8	8700258	A	G	34.375	0.015	0.003	4.50E-09	0.034	0.032	0.2914
rs73210895	8	20021205	G	T	86.151	-0.019	0.002	1.70E-20	-0.006	0.027	0.8133
rs73243877	4	26047616	G	A	117.713	0.029	0.003	2.00E-27	-0.015	0.036	0.6762
rs742036	16	960725	A	G	49.037	-0.014	0.002	2.50E-12	0.052	0.027	0.0527096
rs7424120	2	59313974	T	C	37.025	-0.012	0.002	1.20E-09	-0.030	0.026	0.2418
rs74662600	11	116654959	A	G	103.449	0.083	0.008	2.70E-24	-0.080	0.063	0.1996
rs75033071	8	19652910	G	A	66.372	0.064	0.008	3.70E-16	-0.017	0.166	0.9207
rs75164422	8	126507098	A	G	30.132	-0.032	0.006	4.00E-08	-0.052	0.079	0.5121
rs75268115	11	18301915	G	A	33.577	-0.021	0.004	6.80E-09	0.082	0.043	0.0564196
rs75398587	10	103946480	G	C	41.022	-0.025	0.004	1.50E-10	-0.076	0.066	0.2516
rs75609851	8	19871080	A	G	393.311	-0.199	0.010	1.60E-87	0.004	0.112	0.972
rs75612388	2	28540127	G	A	32.483	-0.034	0.006	1.20E-08	-0.052	0.057	0.3621
rs75679663	17	4667972	A	C	47.826	-0.075	0.011	4.70E-12	0.071	0.077	0.3559
rs7588724	2	25923122	G	A	31.283	-0.012	0.002	2.20E-08	-0.046	0.026	0.0761605
rs75919952	11	116667545	T	C	452.127	-0.097	0.005	2.50E-100	0.098	0.064	0.1273
rs7596814	2	230128204	T	G	34.234	-0.013	0.002	4.90E-09	0.031	0.032	0.3371
rs75999908	1	63406978	A	G	49.653	0.024	0.003	1.80E-12	0.059	0.047	0.2068

rs7600413	2	37067137	G	A	50.028	-0.014	0.002	1.50E-12	-0.049	0.025	0.0515798
rs76172517	2	169514699	C	T	31.837	-0.018	0.003	1.70E-08	-0.044	0.046	0.3427
rs76814754	11	116557959	T	C	42.614	-0.046	0.007	6.70E-11	0.069	0.113	0.5404
rs76862947	16	79746461	C	T	34.072	0.013	0.002	5.30E-09	-0.021	0.028	0.4603
rs76884843	12	57349761	T	C	30.297	-0.048	0.009	3.70E-08	-0.087	0.107	0.4163
rs77009508	6	161004972	G	A	140.807	0.045	0.004	1.80E-32	0.035	0.052	0.5045
rs7704653	5	90255685	G	A	49.203	0.016	0.002	2.30E-12	-0.039	0.029	0.1814
rs77053629	11	61519815	T	C	37.486	-0.028	0.005	9.20E-10	-0.148	0.068	0.0306803
rs7714361	5	112490629	C	A	34.437	0.014	0.002	4.40E-09	-0.011	0.028	0.6951
rs77244849	17	74281391	C	T	44.798	-0.014	0.002	2.20E-11	-0.040	0.029	0.1714
rs7735249	5	53310139	G	C	72.270	0.027	0.003	1.90E-17	0.064	0.039	0.0988394
rs77631110	2	119752450	C	A	42.086	0.052	0.008	8.70E-11	0.010	0.191	0.9566
rs77675968	8	19959983	C	T	92.789	0.054	0.006	5.80E-22	0.073	0.060	0.2182
rs77824033	9	112241136	C	T	32.104	-0.030	0.005	1.50E-08	-0.114	0.074	0.1235
rs7786102	7	25965759	A	G	166.154	-0.028	0.002	5.10E-38	-0.007	0.027	0.7863
rs78025076	4	110569620	T	C	48.136	0.049	0.007	4.00E-12	-0.219	0.138	0.1112
rs78034258	8	19239161	C	T	34.319	-0.023	0.004	4.70E-09	0.008	0.056	0.8796
rs78058190	2	219699999	A	G	255.394	0.082	0.005	1.70E-57	0.082	0.047	0.0766408
rs7826687	8	126645689	G	C	178.645	0.029	0.002	9.60E-41	0.020	0.028	0.4772
rs78297458	11	50611510	C	T	29.787	-0.037	0.007	4.80E-08	-0.198	0.222	0.3707
rs7855395	9	13676484	G	A	33.298	-0.012	0.002	7.90E-09	0.028	0.026	0.2678
rs78588343	6	7249460	A	G	35.550	-0.016	0.003	2.50E-09	-0.067	0.031	0.02712
rs7861679	9	123375589	T	C	31.697	0.012	0.002	1.80E-08	0.003	0.028	0.9045
rs78751065	11	117171413	T	C	58.173	0.058	0.008	2.40E-14	-0.132	0.135	0.3305
rs78933533	6	32604198	A	G	95.276	-0.052	0.005	1.70E-22	0.043	0.063	0.4953
rs79287178	3	172294500	A	G	69.607	0.050	0.006	7.20E-17	0.051	0.063	0.4238
rs79311290	16	85150163	G	A	41.925	0.022	0.003	9.50E-11	0.024	0.056	0.668599
rs7947951	11	13356030	G	A	81.936	0.019	0.002	1.40E-19	0.033	0.025	0.1893
rs7951019	11	118358027	G	T	41.515	-0.037	0.006	1.20E-10	0.098	0.072	0.1698
rs79634051	11	14561945	C	G	50.975	-0.043	0.006	9.40E-13	-0.162	0.070	0.0202302
rs7966846	12	57653582	C	A	39.847	0.014	0.002	2.70E-10	0.003	0.026	0.9172

rs79778375	11	117545083	A	G	33.607	-0.029	0.005	6.70E-09	-0.006	0.053	0.9086
rs79859736	11	116269946	A	G	36.106	0.037	0.006	1.90E-09	0.005	0.063	0.9389
rs79873985	14	24560159	G	A	30.908	-0.045	0.008	2.70E-08	0.187	0.185	0.3113
rs799157	7	73020301	C	T	310.492	-0.086	0.005	1.70E-69	-0.162	0.085	0.0552599
rs79983121	3	127306462	T	C	37.119	0.015	0.002	1.10E-09	-0.039	0.029	0.1719
rs80216311	2	27301057	T	C	96.206	-0.034	0.003	1.00E-22	0.023	0.054	0.671001
rs80276949	10	52373245	A	G	46.778	0.046	0.007	8.00E-12	0.059	0.116	0.612999
rs8046718	16	2462739	T	C	38.600	-0.013	0.002	5.20E-10	-0.039	0.027	0.1423
rs8066985	17	68453345	G	A	40.246	-0.013	0.002	2.20E-10	0.005	0.026	0.8423
rs840974	2	65705581	A	G	35.090	-0.012	0.002	3.10E-09	-0.039	0.027	0.145
rs852388	7	5574239	C	G	40.928	0.016	0.002	1.60E-10	-0.049	0.039	0.2011
rs878409	10	122999550	A	G	31.825	-0.011	0.002	1.70E-08	-0.012	0.025	0.6401
rs878521	7	44255643	A	G	65.157	0.018	0.002	6.90E-16	-0.015	0.031	0.6206
rs879986	8	126569934	G	T	46.722	0.036	0.005	8.20E-12	-0.026	0.118	0.8234
rs880315	1	10796866	C	T	31.276	-0.012	0.002	2.20E-08	0.066	0.026	0.0108901
rs917195	7	30728452	T	C	30.293	-0.013	0.002	3.70E-08	-0.003	0.032	0.9325
rs933574	16	11792700	C	A	38.070	0.012	0.002	6.80E-10	-0.013	0.025	0.608501
rs935168	2	26914787	A	G	46.360	0.014	0.002	9.80E-12	-0.001	0.026	0.9741
rs9375694	6	130356608	A	G	67.355	0.018	0.002	2.30E-16	0.025	0.029	0.3893
rs9376511	6	140611419	G	A	39.532	-0.015	0.002	3.20E-10	0.013	0.031	0.687999
rs9405064	6	31302841	C	A	131.525	0.029	0.003	1.90E-30	0.009	0.052	0.8674
rs9425589	1	172355276	A	G	47.261	-0.014	0.002	6.20E-12	0.001	0.025	0.9776
rs9436661	1	62904575	G	T	1399.394	-0.078	0.002	1.00E-200	-0.017	0.029	0.558
rs947799	11	64851305	T	C	30.895	-0.012	0.002	2.70E-08	-0.004	0.026	0.882
rs9480889	6	109189021	G	C	45.779	0.016	0.002	1.30E-11	0.054	0.037	0.1444
rs9496567	6	100602753	A	G	35.343	-0.014	0.002	2.80E-09	0.030	0.031	0.3341
rs954244	2	121309231	G	C	45.396	0.015	0.002	1.60E-11	-0.017	0.032	0.597901
rs9553567	13	25820101	C	T	30.504	0.015	0.003	3.30E-08	0.082	0.036	0.02274
rs9561643	13	95253131	C	A	60.734	0.017	0.002	6.50E-15	0.001	0.026	0.9725
rs9584870	13	99245866	C	T	34.496	-0.012	0.002	4.30E-09	0.025	0.027	0.3568
rs966541	12	29491528	G	A	32.930	-0.013	0.002	9.60E-09	0.030	0.028	0.2846

rs970069	5	140888248	T	C	44.455	0.016	0.002	2.60E-11	0.005	0.032	0.8808
rs9788220	12	109699616	C	T	38.373	0.016	0.003	5.80E-10	0.028	0.036	0.4395
rs9831084	3	37025661	C	T	35.236	-0.012	0.002	2.90E-09	0.039	0.026	0.1364
rs9890200	17	48624523	C	A	40.155	-0.013	0.002	2.30E-10	-0.006	0.027	0.833
rs9894946	17	7571080	G	A	43.632	-0.018	0.003	4.00E-11	0.029	0.036	0.4078
rs9906319	17	64190994	T	C	36.268	-0.024	0.004	1.70E-09	0.015	0.042	0.7267
rs9923575	16	72230112	T	C	38.055	-0.015	0.003	6.90E-10	0.041	0.032	0.2069
rs9943778	12	22765864	G	A	42.649	-0.015	0.002	6.50E-11	0.006	0.029	0.8306
rs9944241	15	51900651	C	T	36.988	-0.013	0.002	1.20E-09	0.010	0.025	0.692201
rs9970140	1	61684288	G	A	46.395	-0.026	0.004	9.70E-12	0.051	0.047	0.2764
rs998584	6	43757896	A	C	406.426	0.040	0.002	2.20E-90	0.015	0.025	0.550301
rs9991328	4	89713121	T	C	56.691	0.015	0.002	5.10E-14	-0.032	0.025	0.2061

TG: Triglycerides; TC: Total cholesterol; HDL-C: High-Density Lipoprotein Cholesterol; LDL-C: Low-Density Lipoprotein Cholesterol; SNP: single nucleotide polymorphism; SE: standard error

Supplementary materials Table 2 Evaluating Pleiotropy in Hypertension,TG, TC, LDL-C, HDL-C and Gout Link via Mendelian Randomization

exposure	outcome	egger_intercept	se	*p*
**Hypertension**	**gout**	0.006	0.005	0.293
**TG**	**gout**	0.004	0.002	0.151
**TC**	**gout**	-0.004	0.003	0.294
**LDL-C,**	**gout**	0.004	0.003	0.121
**HDL-C**	**gout**	-0.006	0.002	0.010

Supplementary materials Table 3. Characteristics of GWAS participants included in the MR study.

Items	GWAS ID	Consortium	Sample size	Enrolled SNPs (n）	Population
Hypertension	ukb-b-14057	MRC-IEU	462,933	297	Europeans
TC	ebi-a-GCST90025953	NA	437,878	303	Europeans
TG	ieu-b-111	UK Biobank	441,016	464	Europeans
LDL-C	ieu-b-5089	UK Biobank	201,678	460	Europeans
HDL-C	ieu-b-109	UK Biobank	403,943	632	Europeans
Gout	finn-b-M13_GOUT	FinnGen	150,797	NA	Europeans

Supplementary materials Table 4. Summary of each Mendelian randomization method.

MR method	Strengths and weaknesses
Inverse-variance weighted	The inverse-variance weighted method calculates a weighted mean of the effects of individual genetic variants on the outcome. This approach yields an estimate akin to that obtained from Mendelian Randomization (MR) analysis with individual-level data, under the assumption that the genetic variants are independent. It is statistically the most powerful method provided all variants are valid instruments. However, its estimates may be biased in the presence of directional pleiotropy, which occurs when the mean of the pleiotropy distribution deviates from zero[1, 2].
MR-Egger	This method quantifies directional pleiotropy and compensates for it, offering an unbiased estimate regardless of the presence of pleiotropic effects in all SNPs. It operates under the InSIDE assumption, which posits that the magnitude of pleiotropic effects is unrelated to the influence of the variants on the exposure. However, this assumption cannot be empirically verified. The approach is sensitive to outliers and generally less efficient, leading to broader confidence intervals[3, 4].
Weighted median	This method is resilient to outliers and furnishes an unbiased estimate even when as many as half of the SNPs violate the instrumental variable assumptions. However, it may exhibit reduced efficiency under such circumstances[5].
MR-PRESSO	The method identifies and removes potentially pleiotropic outliers, but may suffer from a high false-positive rate when there are multiple invalid instrumental variables[6].
Multivariable MR	This extended univariable Mendelian randomization (MR) method estimates the effect of multiple exposures on an outcome. It can account for a secondary exposure acting as a confounder, mediator, pleiotropic pathway, or collider. However, it relies on knowledge of the covariance between the SNP's effects on each exposure, which is often unavailable in standard GWAS summary data[7, 8].
Mediation Mendelian Randomization	Mediation Mendelian Randomization (MMR) offers several advantages over traditional observational mediation analysis. It allows for the decomposition of a total exposure effect into direct and indirect (mediated) components, providing more mechanistic insights. By utilizing genetic variants as instrumental variables, MMR can help minimize concerns about confounding. Additionally, MMR can handle multiple mediators simultaneously. However, this approach also has notable limitations. It requires knowledge of the genetic associations with both the exposure and mediator, which is not always available in existing data. MMR also assumes no pleiotropic effects of the genetic instruments beyond the exposure and mediator, and may have reduced statistical power compared to standard Mendelian randomization, especially when the indirect effect is small. Interpretation can also be complex, particularly when the mediator and outcome have bidirectional relationships[8].

1. Burgess S, Butterworth A, Thompson SG. Mendelian randomization analysis with multiple genetic variants using summarized data. Genet Epidemiol. 2013;37(7):658-65. Epub 20130920. doi: 10.1002/gepi.21758. PubMed PMID: 24114802; PubMed Central PMCID: PMCPMC4377079.
2. Slob EAW, Burgess S. A comparison of robust Mendelian randomization methods using summary data. Genet Epidemiol. 2020;44(4):313-29. Epub 20200406. doi: 10.1002/gepi.22295. PubMed PMID: 32249995; PubMed Central PMCID: PMCPMC7317850.
3. Bowden J, Davey Smith G, Burgess S. Mendelian randomization with invalid instruments: effect estimation and bias detection through Egger regression. Int J Epidemiol. 2015;44(2):512-25. Epub 20150606. doi: 10.1093/ije/dyv080. PubMed PMID: 26050253; PubMed Central PMCID: PMCPMC4469799.
4. Burgess S, Thompson SG. Interpreting findings from Mendelian randomization using the MR-Egger method. Eur J Epidemiol. 2017;32(5):377-89. Epub 20170519. doi: 10.1007/s10654-017-0255-x. PubMed PMID: 28527048; PubMed Central PMCID: PMCPMC5506233.
5. Bowden J, Davey Smith G, Haycock PC, Burgess S. Consistent Estimation in Mendelian Randomization with Some Invalid Instruments Using a Weighted Median Estimator. Genet Epidemiol. 2016;40(4):304-14. Epub 20160407. doi: 10.1002/gepi.21965. PubMed PMID: 27061298; PubMed Central PMCID: PMCPMC4849733.
6. Verbanck M, Chen CY, Neale B, Do R. Detection of widespread horizontal pleiotropy in causal relationships inferred from Mendelian randomization between complex traits and diseases. Nat Genet. 2018;50(5):693-8. Epub 20180423. doi: 10.1038/s41588-018-0099-7. PubMed PMID: 29686387; PubMed Central PMCID: PMCPMC6083837.
7. Sanderson E, Davey Smith G, Windmeijer F, Bowden J. An examination of multivariable Mendelian randomization in the single-sample and two-sample summary data settings. Int J Epidemiol. 2019;48(3):713-27. doi: 10.1093/ije/dyy262. PubMed PMID: 30535378; PubMed Central PMCID: PMCPMC6734942.
8. Sanderson E. Multivariable Mendelian Randomization and Mediation. Cold Spring Harb Perspect Med. 2021;11(2). Epub 20210201. doi: 10.1101/cshperspect.a038984. PubMed PMID: 32341063; PubMed Central PMCID: PMCPMC7849347.
